# Comparative Effectiveness of Community-Based vs Clinic-Based Healthy Choices Motivational Intervention to Improve Health Behaviors Among Youth Living With HIV: A Randomized Clinical Trial

**DOI:** 10.1001/jamanetworkopen.2020.14650

**Published:** 2020-08-26

**Authors:** Sylvie Naar, Gabriel Robles, Karen Kolmodin MacDonell, Veronica Dinaj-Koci, Kit N. Simpson, Phebe Lam, Jeffrey T. Parsons, K. Marie Sizemore, Tyrel J. Starks

**Affiliations:** 1 Center for Translational Behavioral Science, Florida State University College of Medicine, Tallahassee; 2 School of Social Work, Rutgers University, New Brunswick, New Jersey; 3 Department of Family Medicine and Public Health Sciences, Wayne State University, Detroit, Michigan; 4 College of Health Professions, Medical University of South Carolina, Charleston; 5 University of Windsor, Faculty of Arts, Humanities and Social Sciences, Windsor, Ontario, Canada; 6 Mindful Designs, Teaneck, New Jersey; 7 Department of Psychology, Hunter College, City University of New York, New York; 8 Health Psychology and Clinical Science Program, The Graduate Center, City University of New York, New York

## Abstract

**Importance:**

Youth living with HIV make up one-quarter of new infections and have high rates of risk behaviors but are significantly understudied. Effectiveness trials in real-world settings are needed to inform program delivery.

**Objective:**

To compare the effectiveness of the Healthy Choices intervention delivered in a home or community setting vs a medical clinic.

**Design, Setting, and Participants:**

This randomized clinical trial was conducted from November 1, 2014, to January 31, 2018, with 52 weeks of follow-up. Participants, recruited from 5 adolescent HIV clinics in the United States, were youths and young adults living with HIV aged 16 to 24 years who were fluent in English, were currently prescribed HIV medication, had a detectable viral load, and had used alcohol in the past 12 weeks. Individuals with an active psychosis that resulted in an inability to complete questionnaires were excluded. Data were analyzed from May to December, 2019.

**Interventions:**

Participants were randomized to receive the Healthy Choices intervention in either a home or clinic setting. Four 30-minute individual sessions based on motivational interviewing to improve (1) medication adherence and (2) drinking behavior were delivered during 10 weeks by trained community health workers. In session 1, participants chose which behavior to discuss first. Using motivational interviewing strategies, the community health worker elicited motivational language, guided the development of an individualized change plan while supporting autonomy, delivered feedback, and addressed knowledge gaps. Session 2 focused on the second target behavior. In subsequent sessions, community health workers reviewed the individualized change plan, monitored progress, guided problem solving, and helped maintain changes made.

**Main Outcomes and Measures:**

Primary outcomes were viral load and alcohol use change trajectories during 52 weeks of follow-up. Alcohol use severity and frequency were measured using the Alcohol, Smoking and Substance Involvement Screening Test (ASSIST) (scores range from 0 to 33, with higher scores indicating greater severity of alcohol-related problems) and number of drinks consumed each day over a 30-day period, with timeline followback.

**Results:**

A total of 183 young people living with HIV (145 male [79.2%]; mean [SD] age, 21.4 [1.9] y) were randomized to the home setting (n = 90) or clinical setting (n = 93). Using growth-curve analysis, both groups showed declines in viral load after the intervention: among participants with available viral load information, in the home group, 12 participants (21%) had an undetectable viral load at 16 weeks, 12 (22%) at 28 weeks, and 10 (20%) at 52 weeks; in the clinic group, 16 participants (24%) had an undetectable viral load at 16 weeks, 20 (39%) at 28 weeks, and 18 (35%) at 52 weeks. However, the clinic group maintained gains, whereas those counseled at home had a significantly different and increasing trajectory during follow-up (unstandardized β = −0.07; 95% CI,−0.14 to −0.01; *P* = .02). A similar pattern was observed in ASSIST scores during follow-up, with reduced ASSIST scores in the clinic group (unstandardized β = −0.44; 95% CI,−0.81 to −0.07; *P* = .02).

**Conclusions and Relevance:**

In this trial, the Healthy Choices intervention resulted in improvements in viral load and alcohol use over 12 months. Unexpectedly, the clinic setting outperformed home-based delivery for viral suppression. Although cross-sectional differences in ASSIST scores were nonsignificant, clinic delivery did improve the trajectory of ASSIST scores during follow-up. Thus, clinics may be the more effective site for interventions aimed at viral load reduction for young people living with HIV.

**Trial Registration:**

ClinicalTrials.gov Identifier: NCT01969461

## Key Points

**Question:**

Is the Healthy Choices intervention program more effective if delivered in an HIV clinic vs remotely?

**Findings:**

In this randomized clinical trial, 183 HIV-positive youths and young adults underwent four 30-minute sessions with community health workers targeting medication adherence and alcohol use; those randomized to the clinic setting had greater improvement in viral load during 1-year follow-up than those randomized to remote (home-based) delivery. Clinic, but not home, delivery was also associated with a trajectory of decreasing alcohol use severity during follow-up.

**Meaning:**

These findings suggest that Healthy Choices sessions provided in the clinic have greater long-term effects on viral load than sessions delivered at home.

## Teaser

This randomized clinical trial assesses the comparative effectiveness of the Healthy Choices intervention when delivered in a clinic- vs home-based setting on medication adherence and alcohol use among youths and young adults living with HIV.

## Introduction

Youth living with HIV make up more than one-fourth of new infections in the United States^1^ and have high rates of risk behaviors, such as alcohol use and nonadherence to medication,^2,3^ but are significantly understudied. Alcohol consumption among persons with HIV exacerbates health problems and accelerates HIV disease progression.^3,4,5,6^ Optimal adherence to antiretroviral treatment (ART) decreases morbidity and mortality,^4,5,7,8,9^ the potential for the development of drug-resistant strains of HIV,^10,11,12^ and HIV infectiousness.^13,14,15,16^ Healthy Choices, a 4-session, 10-week intervention based on motivational interviewing,^17^ is the only intervention (to our knowledge) to demonstrate improvements in viral load and alcohol trajectories in youths living with HIV in a full-scale, multisite randomized trial when delivered by members of the research team.^18,19^ Thus, testing the intervention in a real world clinical setting when delivered by members of the HIV clinical care team, such as community health workers (CHWs) and local supervisors, can provide evidence to inform practitioners on clinically effective and cost-effective treatments that are available prior to dissemination on a wider scale.^20,21,22,23,24^ Such effectiveness trials are the next stage on the translational science spectrum.^25^

In the original Healthy Choices trial, although most youths attended at least 1 session, less than half completed all 4 sessions.^26^ It is possible that short-term viral load improvements may have been sustained if participants received a full exposure of treatment. Many researchers have suggested home-based service delivery to increase access to and engagement in behavioral health services, especially when delivered by CHWs, but this remains untested in youths living with HIV.^27,28^ Thus, this comparative effectiveness trial compared Healthy Choices in a home-based vs clinic-based setting, delivered by CHWs and supervised by members of the clinical care teams, on primary outcomes of viral load and alcohol use. We hypothesized that home-based delivery would result in greater improvements in viral load and alcohol use compared with clinic-based services by decreasing barriers to participation and by delivering treatments in the youth’s natural ecology.

## Methods

### Participants and Procedures

Participants were recruited from 5 adolescent HIV clinics in Chicago, Illinois; Detroit, Michigan; Memphis, Tennessee; Los Angeles, California; and Philadelphia, Pennsylvania. Inclusion criteria included positive HIV status, age of 16 to 24 years, ability to complete questionnaires in English (96.7% of participants spoke English at home), currently prescribed ART, detectable viral load within the last 4 weeks, and alcohol use in the past 12 weeks. Exclusion criteria included having an active psychosis that resulted in an inability to complete questionnaires. Participants were randomized to intervention delivery in the clinic (n = 93) or at home (n = 90).

**Figure 1.**
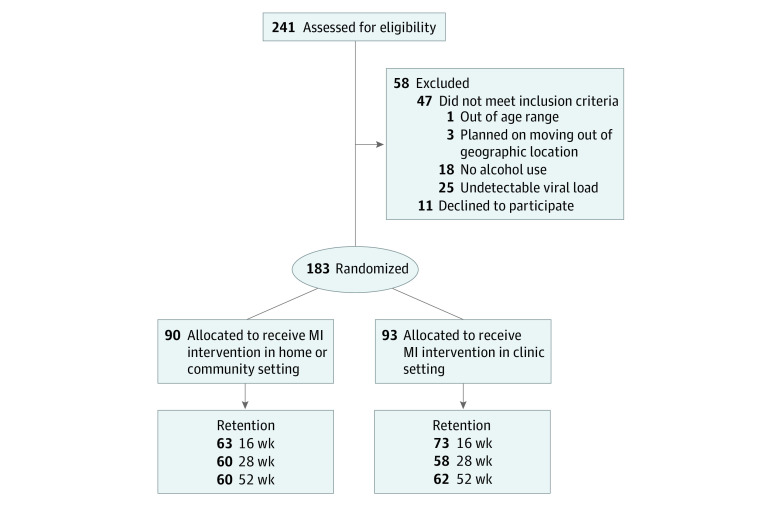
Participant Flow Chart MI indicates motivational interview.

This report follows the Consolidated Standards of Reporting Trials (CONSORT) reporting guideline for randomized clinical trials (Figure 1). The trial protocol (Supplement 1) was approved by each site’s institutional review board, and a certificate of confidentiality was obtained from the National Institutes of Health. Data were collected between November 1, 2014, and January 31, 2018. Clinicians at each site gave a description of the study to potential participants. If the patient was interested, a researcher obtained verbal consent for screening. When eligible, written informed consent was obtained and a waiver of parental consent was permitted for participants younger than 18 years. Using a Qualtrics survey, participants were then randomized (1:1 stratified by site), and intervention sessions were scheduled immediately after the baseline assessment. Retention strategies included reminder calls at different times of the day and collaboration with clinic outreach staff. Bus tickets or parking reimbursement were given to youths randomized to the clinic as consistent with the sites’ standard of care.

### Healthy Choices Intervention

The 4-session intervention was identical for the clinic and home locations and has been previously described.^18,29^ Community health workers were paraprofessional staff with at least a high school degree or equivalent and no more than a bachelor’s degree. Supervisors were clinicians with a master’s degree already employed by the clinic. In the original study, participants could focus on sexual risk or other substance use, but for this study the focus was viral suppression via improved adherence and reduced alcohol use. In session 1, participants chose which of the 2 behaviors to discuss first, and, using standard motivational interviewing strategies, the CHW engaged the youth, collaboratively focused the conversation on the target behavior, evoked motivational statements, and guided the development of an individualized change plan based on readiness to change while utilizing motivational interviewing strategies to support autonomy. The CHW delivered structured personalized feedback on risk behaviors and health status based on the baseline assessment and offered informative handouts to address any knowledge gaps. The second session (week 2) followed the same format but focused on the second target behavior. In the subsequent 2 sessions (weeks 6 and 10), the CHW reviewed the individualized change plan, continued to monitor and encourage progress, and guided the youth to problem-solve barriers and maintain any changes made. Based on randomization, the intervention was delivered in a private room in the clinic or in the home or a community-based location of the youth’s choice. Participants received no monetary incentives for attending the intervention sessions.

### Intervention Fidelity

Community health workers participated in a 2.5-day training by members of the Motivational Interviewing Network of Trainers. Supervisors attended the 2.5-day training and an additional half day of supervision training. The CHWs and supervisors each submitted 4 audio-recorded roleplays for clearance using the “beginning proficiency” threshold on the Motivational Interviewing Treatment Integrity codes.^30^ A trainer then observed 6 coaching sessions and provided feedback to local supervisors on coaching and feedback strategies. During the trial, supervisors provided 30 minutes of weekly coaching to CHWs. Supervisors were required to review at least 1 audio-recorded session conducted by the CHW per month. Supervisors also participated in a monthly group call with trainers, during which supervisors at all active sites discussed issues that emerged in sessions and effective coaching strategies. A review and coding of 25% of session recordings was conducted to assure motivational interviewing was being administered with fidelity. Supervision sessions were also recorded and coded for motivational interviewing fidelity. When either supervision sessions or participant sessions fell below the threshold, a trainer contacted the site supervisor for remediation.

### Data Collection Methods

Baseline assessment data were collected in the clinic. The alcohol use timeline followback questions were recorded on paper by an interviewer, and all other data points were collected using audio-computer-assisted self-interviewing. The same measures were completed at baseline and at 16, 28, and 52 weeks. Participants were paid $50 for each data collection.

### Variables Measured

Age, race/ethnicity, biological sex, gender identity, and sexual identity were assessed using audio-computer-assisted self-interviewing. Blood for quantitative plasma HIV RNA assays was collected within a 2-week window surrounding the data collection time point. Viral load values reported as below the level of detection were set to 20 copies per milliliter. All viral load values were transformed to log10 for the statistical analyses.

We measured 2 dimensions of alcohol use: severity of problems and number of drinks per week. Severity of problems was measured using the Alcohol, Smoking, and Substance Involvement Screening Test (ASSIST), a brief screening tool to assess the individual’s level of alcohol use and problems related to use.^31^ This test yields a summary score for severity of alcohol use ranging from 0 to 33, with higher scores indicating greater severity. Number of drinks per day was measured using the timeline followback procedure.^32^ The interviewer asked the participant to use a calendar to identify the number of standard drinks consumed each day over a 30-day period. The number of drinks consumed in the participants’ heaviest drinking week in the 30-day period was used as the measure of drinks per week as in the original Healthy Choices trial.^18^

### Statistical Analysis

#### Analysis Methods

Data analysis was conducted between May and December 2019. The outcome of interest in this effectiveness trial was whether the response trajectory for the motivational interviewing–based behavioral intervention improved if the intervention sessions were delivered in a home vs clinic setting. The period of interest was 52 weeks from baseline. Response was measured using 2 indicators of alcohol use (ASSIST and timeline followback) and 1 indicator of antiretroviral medication adherence (viral load). Outcomes from behavioral interventions are often characterized by an early large response that attenuates over time. This curvilinear response can be identified for each individual, and the statistical differences in the mean response curves can be assessed for groups using latent growth curve analysis (LGCA) This statistical approach is commonly used in studies of behavioral health interventions^18,33,34,35^ because it considers change over time in terms of an underlying, latent, unobserved process, which is what we aim to affect with behavioral interventions. The LGCA results are often quite similar to findings observed using multilevel modeling with fixed and random effects.^36,37^ However, they have the advantage of being able to capture unique trajectories that may be of interest to the researcher, and they can avoid potential aggregation bias because trajectory changes are captured at the level of the individual. The drawback of using LGCA is that it is based on structural equation models, which are less well known to clinicians, and that LGCA model fitting is complex and requires detailed documentation (eAppendix in Supplement 2). We chose to use LGCA for this study because it allows the reader to more easily compare these effectiveness trial results to the original motivational interviewing study report,^18^ and it assures that we used a flexible model in which the unit of analysis is the individual’s trajectory over time. The threshold for determining significance was *P* < .05. We used 2-sided tests of significance in all cases.

#### Study Power

The planned overall sample size was 500 patients, with 100 patients per site for 5 sites. Assuming that participant observations were independent, if the setting accounted for at least 1% of the unique variance in the alcohol outcome measures, we would have 80% power to detect a significant difference in the alcohol measures. The slow accrual of these very high-risk subjects limited our actual sample size to 183. Post hoc power analysis and details from our original power estimation are provided in the eAppendix in Supplement 2. To assess the effectiveness of our randomization procedure, we used χ^2^ tests and ANOVA to investigate the equivalence of possible confounders across each condition at baseline. Specifically, we examined proportional differences across demographic characteristics (race, education, employment status, sexual identity, and gender identity) and mean differences across age and baseline outcome values (viral load and alcohol severity and frequency).

We conducted analyses to test for the presence of differential attrition between conditions (eTable 1 in Supplement 2). First, we conducted χ^2^ analyses to test for differential attrition by study arm at each follow-up. We also tested for differential attrition at each follow-up across site, as well as several demographic characteristics (race, education, employment status, sexual identity, and gender identity). Finally, we conducted a series of point-biserial correlations to examine attrition at each follow-up point by age and baseline outcome values (viral load and alcohol severity and frequency).

#### Multivariable Modeling of Effect

A piece-wise LGCA approach,^18,33,34,35^ as discussed previously, was used to assess between-condition differences in viral load and frequency across postintervention time points. Piece-wise LGCA is well suited to situations in which a developmental trajectory has a known pivot point (also called a knot). In these instances, slopes can be specified that quantify each component of the trajectory. Due to the need to correct for baseline differences in ASSIST scores when calculating follow-up values, we utilized an alternative growth modeling procedure.^38^ This analysis modeled only postintervention points corrected for baseline ASSIST values.

Models were estimated using Mplus, and examples of the general coding approach is available through their website.^39^ For each outcome, a model was estimated that included 3 latent growth components. These included an intercept (with factor loadings uniformly equal to 1) and 2 slopes. Factor loadings for slopes were scaled in constant 4-week units of time. Slope 1 captured pre- to postintervention change. Loadings were set to zero at baseline and 4 at the 16- week follow-up and then held constant at 4 thereafter. Slope 2 estimated the linear change over the postintervention follow-up period. Factor loadings for this slope were set to zero for baseline and 16-week follow-up, 7 for the 28-week follow-up, and 13 for the 52-week follow-up. The model included condition (home vs clinic) as a variable in the latent growth factors and a fixed effect for site. All models were estimated using full-information maximum likelihood estimation. For model fit criteria and details of sequential model building see the eAppendix in Supplement 2.

## Results

Demographic data for the total sample are presented in Table 1. A total of 183 participants were included, of whom 145 identified as male (79.2%), 25 as female (13.7%), and 13 as transgender or gender nonconforming (7.1%). The population’s mean (SD) age was 21.4 (1.9) years, and 145 participants (82.5%) were Black. Conditions (ie, clinic vs home delivery) were equivalent with respect to demographic viral load and drinking frequency reported at baseline. Participants randomized to clinic delivery reported higher ASSIST scores (mean [SD] score, 13.18 [9.51]) at baseline compared with those randomized to home delivery (mean [SD] score, 10.22 [7.86]; *t*_181_ = −2.29). In subsequent analyses of primary outcomes, demographic covariates were excluded from the growth curve models. Models for ASSIST scores included baseline scores as a covariate in the estimation of latent growth factors to adjust for baseline differences between delivery condition. χ^2^ Analyses did not reveal differential attrition based on condition. When examining demographic characteristics, we did find differential attrition based on gender identity at 52 weeks (χ^2^_4_ = 10.03; *P* = .04). Specifically, transgender women were less likely to be retained in the 52-week follow-up compared with cisgender male and cisgender female participants. *t* Tests provided no evidence of differential attrition by age or baseline outcome values (viral load and alcohol severity and frequency). With respect to intervention dose receipt, 35.5% of the sample completed 0 sessions, 26.8% completed at least 2 sessions, and 25.1% completed all 4 sessions. There were no differences between groups in dose received (χ^2^_4_ = 3.39; *P* = .495).

**Table 1.** Baseline Characteristics and Analysis of Randomization Success

Characteristic	No. (%)		
	Overall	Home condition	Clinic condition
Total	183	90 (49.2)	93 (50.8)
Race/ethnicity			
Black	151 (82.5)	79 (87.8)	72 (77.4)
Latino	16 (8.7)	5 (5.6)	11 (11.8)
White	3 (1.6)	1 (1.1)	2 (2.2)
Other	13 (7.1)	5 (5.6)	8 (8.6)
Educational attainment			
Less than high school	52 (28.4)	27 (30.0)	25 (26.9)
High school or GED	67 (36.6)	34 (37.8)	33 (35.5)
Some college	64 (35.0)	29 (32.2)	35 (37.6)
Employed			
Yes	90 (49.2)	49 (54.4)	41 (44.1)
No	93 (50.8)	41 (45.6)	52 (55.9)
Sexual identity			
Heterosexual	38 (21.0)	18 (20.2)	20 (21.7)
Gay or lesbian	103 (56.9)	50 (56.2)	53 (57.6)
Bisexual or questioning	40 (22.1)	21 (23.6)	19 (20.7)
Gender identity			
Male	145 (79.2)	73 (81.1)	72 (77.4)
Female	25 (13.7)	9 (10)	16 (17.2)
Transgender or gender nonconforming	13 (7.1)	8 (8.9)	5 (5.4)
Age, mean (SD), y	21.4 (1.9)	21.6 (1.9)	21.2 (1.8)
HIV viral load [log], mean (SD)	3.68 (1.23)	3.67 (1.28)	3.69 (1.20)
Alcohol ASSIST score, mean (SD)^a^	11.73 (8.84)	10.22 (7.86)	13.18 (9.51)
Frequency of drinks, mean (SD)^b^	17.98 (36.07)	13.14 (17.43)	22.66 (47.28)

Abbreviation: GED, General Educational Development.

^a^ Scores range from 0 to 33, with higher scores indicating greater severity of alcohol-related problems.

^b^ Drinking frequency is the number of standard drinks consumed in the heaviest 7-day drinking period recorded in the timeline followback interview.

### Primary Outcomes

#### Viral Load

**Figure 2.**
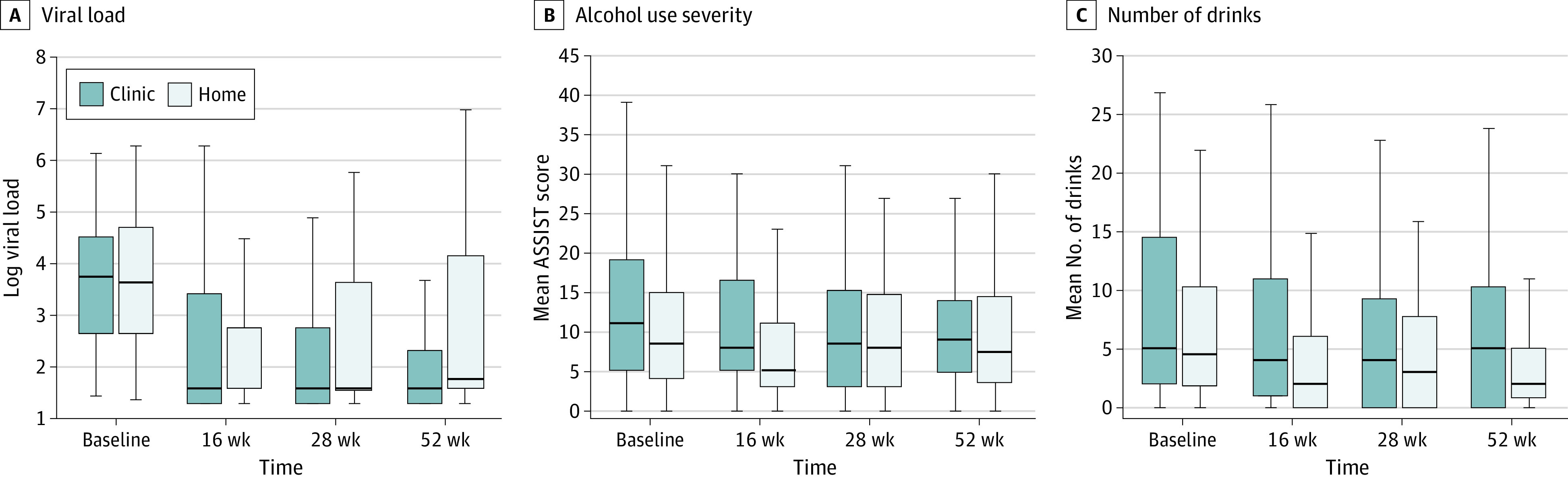
Primary Outcomes Over Time by Condition The boxes represent the interquartile range; the line inside each box, the median; and the whiskers, the most extreme values that fall within 1.5 times the interquartile range above quartile 3 and below quartile 1. ASSIST indicates Alcohol, Smoking and Substance Involvement Screening Test .

In the viral load model (Figure 2), there were no between-condition differences in change from preintervention to postintervention. In contrast, the clinic condition had significantly greater reductions in viral load over the postintervention follow-up period. Among participants with available viral load data, in the home group, 12 participants (21%) had an undetectable viral load at 16 weeks, 12 (22%) at 28 weeks, and 10 (20%) at 52 weeks; in the clinic group, 16 participants (24%) had an undetectable viral load at 16 weeks, 20 (39%) at 28 weeks, and 18 (35%) at 52 weeks (unstandardized β*_slope2_* = −0.07; 95% CI, −0.14 to −0.01; *P* = .02) (Table 2 and eTable 2 in Supplement 2).

**Table 2.** Results of Latent Growth Curve Models for HIV-Related Outcomes^a^

Variable	Viral load		ASSIST (severity)		No. of drinks (frequency)	
	Unstandardized β (95% CI)	*P* value	Unstandardized β (95% CI)	*P* value	Unstandardized β (95% CI)	*P* value
Intercept						
Threshold/intercept	3.52 (3.12 to 3.91)	<.001	8.09 (5.22 to 10.51)	<.001	1.62 (1.21 to 2.02)	<.001
Clinic	0.05 (−0.25 to 0.37)	.73	3.03 (0.59 to 5.47)	.02	0.28 (−0.08 to 0.64)	.12
Slope 1						
Intercept	−0.27 (−0.43 to −0.10)	.001	−0.76 (−1.44 to −0.08)	.03	−0.09 (−0.21 to 2.03)	.09
Clinic	0.00 (−0.13 to 0.12)	.98	0.01 (−0.71 to 0.71)	.98	0.05 (−0.06 to 0.15)	.39
Slope 2						
Intercept	0.00 (−0.06 to 0.06)	.98	0.35 (−0.09 to 0.79)	.13	−0.03 (−0.14 to 0.08)	.60
Clinic	−0.07 (−0.14 to −0.01)	.02	−0.44 (−0.81 to −0.06)	.02	−0.02 (−0.09 to 0.05)	.60

^a^ All models presented controlled for the fixed effect of site in the prediction of all latent growth factors. The Alcohol, Smoking and Substance Involvement Screening Test (ASSIST) model also controlled for baseline ASSIST scores when predicting latent growth slope factors due to the failure of randomization with respect to this outcome. See eTable 2 in Supplement 2 for comprehensive model coefficients.

**Figure 3.**
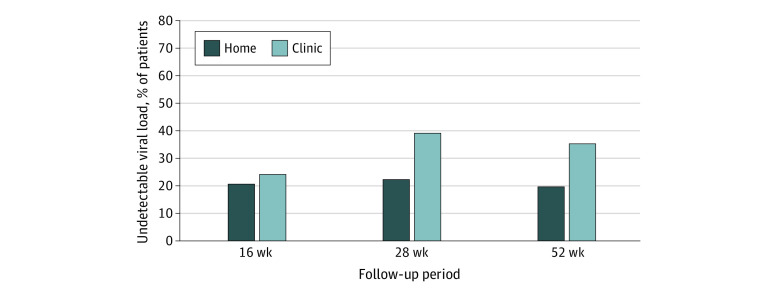
Viral Suppression Over Time by Condition

As a result of divergent trajectories, significant differences between intervention conditions emerged over time postintervention. We did not observe a significant mean difference in log viral loads at the 16-week follow-up (unstandardized β = 0.02; 95% CI, −0.41 to 0.46; *P* = .92) between conditions. Furthermore, at the 28-week follow-up, there was no significant difference in log viral loads (unstandardized β = −0.21; 95% CI, −0.58 to 0.17; *P* = .29). However, we saw a significant mean difference at 52 weeks (unstandardized β = −0.58; 95% CI, −1.06 to −0.10; *P* = .02) for log viral loads between the clinic and home conditions. The observed means provide a direct illustration of the clinical significance of this difference. Those who received the intervention in the clinic had, on average, approximately 355 fewer viral copies per milliliter compared with those who received home delivery. Figure 3 shows percent undetectable at follow-up points, with 0% undetectable at baseline based on eligibility requirements.

#### Alcohol Use

In models of alcohol severity (ASSIST), when correcting for baseline values, the condition was significantly associated with trajectory over time postintervention. Specifically, whereas the home-based condition had a nonsignificant trend over the follow-up period (unstandardized β*_slope 2 intercept_* = 0.35, 95% CI, 0.09 to 0.79; *P* = .13), representing a return to near-baseline levels, the clinic-condition slope was significantly smaller (unstandardized β_s_*_lope2_* = −0.44; 95% CI, −0.81 to −0.06, *P* = .02) in Table 2. Cross-sectional differences between estimated group means were uniformly nonsignificant across 16-week (unstandardized β = 1.76; 95% CI, −0.59 to 4.11; *P* = .14), 28-week (unstandardized *β* = 0.44; 95% CI, −1.46 to 2.34; *P* = .65), and 52-week (unstandardized β = −1.76; 95% CI, −4.21 to 0.69; *P* = .16) follow-up points; however, the direction of nonsignificant differences reversed owing to differences in trajectory, with lower scores indicated among youths who received the intervention in a clinic. The standard deviation of baseline-adjusted ASSIST scores at the 52-week follow-up was approximately 7.10. The cross-sectional difference between conditions would therefore correspond to a Cohen *d* = 0.25, indicating a small effect size.

For alcohol use frequency (Table 2), there were no between-condition differences in change from preintervention to postintervention (unstandardized β = 0.05; 95% CI, −0.06 to 0.15; *P* = .39). At the 16-week assessment period, we observed a between-group difference (unstandardized β = 0.46; 95% CI, 0.02 to 0.91; *P* = .04). Estimates indicated that these differences persisted at 28 weeks (unstandardized β = 0.41; 95% CI, 0.06 to 0.75; *P* = .02). Differences attenuated at 52 weeks (unstandardized β = 0.32; 95% CI, −0.13 to 0.76; *P* = .17). The clinical significance of between-group differences is illustrated by an examination of group means. The observed differences between groups are largest at the 16-week follow-up (4.03 drinks in the heaviest drinking week) and attenuate to a mean difference of 2.5 drinks at 52 weeks. Given that responses ranged from 0 to 39 drinks, these differences are modest in magnitude relative to the variability in drinking.

## Discussion

In this randomized clinical trial, Healthy Choices resulted in improvements in viral load and alcohol use (severity and frequency) during 12 months of follow-up when delivered by CHWs to a primarily racial/ethnic-minority sample of youth living with HIV in the clinic or at home or in the community. Unlike in the original Healthy Choices trial, viral load improvements were sustained over time, possibly owing to the improved regimens in the current trial compared with the state of antiretroviral therapy in the original trial a decade ago. Contrary to our hypothesis, the clinic-delivered intervention outperformed the home- or community-based delivery with regard to viral suppression and alcohol severity; it may also be less costly and easier to implement. However, most youth did not receive a full dose of treatment, and intent-to-treat analysis suggested improvements even with small doses of intervention. Future studies could consider an adaptive design to test the effect of a single session, with additional sessions offered to nonresponders.^40^ Furthermore, because home- and community-based delivery did not result in increased session attendance, other modes of delivery, such as videoconferencing and mobile apps, should be considered to increase session attendance.

More than half of participants did not achieve viral suppression, regardless of condition. However, youths who still have unsuppressed viral load in the current era of simplified regimens and effectiveness of antiretroviral therapy, even with less-than-perfect adherence,^41^ may be faced with many more psychosocial barriers than youths who struggled with viral suppression a decade ago. Thus, improving viral suppression in one-quarter to one-third of such a high-risk group has significant public health implications. While brief interventions may be more easily implemented in clinic settings, a more intensive intervention may be needed to sufficiently halt viral replication among youths at highest risk.^42,43^ Some youths may benefit from motivational interviewing alone, and some may benefit more from motivational interviewing combined with cognitive-behavioral skills-building interventions.^44^

### Limitations

Limitations include lower-than-optimal study retention, although this is not unusual for a real-world effectiveness trial. In addition, generalizability of the sample, recruited from academic medical settings, may be limited.

## Conclusions

This randomized clinical trial found that the Healthy Choices intervention resulted in greater improvements in viral load and alcohol use when delivered in a clinic vs a home setting. Given these findings, in addition to considering designs to develop an adaptive intervention and technology-based delivery, future directions may include conducting a cost-effectiveness analysis of clinic vs community-based delivery.

## Notes

Supplement 1. **Trial Protocol**

Supplement 2. **eAppendix.** Data Analysis **eTable 1.** Analyses of Attrition **eTable 2.** Comprehensive Model Coefficients

Supplement 3. **Data Sharing Statement**
