# Supplementary material for: Comparative Effectiveness of Community-Based vs Clinic-Based Healthy Choices Motivational Intervention to Improve Health Behaviors Among Youth Living With HIV: A Randomized Clinical Trial
Source: JAMA Netw Open. 2020 Aug 26;3(8):e2014650. doi: 10.1001/jamanetworkopen.2020.14650 (PMC7450347; doi:10.1001/jamanetworkopen.2020.14650)
Supplement: Supplement 1. — Trial Protocol [file jamanetwopen-3-e2014650-s001.pdf]

ATN 129
Version 1.0
April 11, 2014

COMPARING THE EFFECTIVENESS OF TWO ALCOHOL+ADHERENCE INTERVENTIONS FOR HIV+ YOUTH

(Healthy Choices 2; HC2)

Wayne State University
(WSU)

Sponsored by:
National Institute on Alcohol Abuse and Alcoholism
(NIAAA)

Through Collaboration of:
A Multi-Center Study of the Adolescent Medicine Trials Network for HIV/AIDS Interventions
(ATN)

**Protocol Co-Chair:**	**Sylvie Naar-King, PhD**
**Protocol Co-Chair:**	**Phebe K. Lam, PhD**
**NICHD Program Scientific Director:**	**Bill G. Kapogiannis, MD**
**NICHD Health Science Administrator:**	**Sonia S. Lee, PhD**
**Protocol Specialist:**	**Dina Monte, RN, BSN**
**ATN Site PI Representative:**	**Aditya H. Gaur, MD**
**ATN Site SC Representative:**	**Rachel M. Jackson, APN, CFNP**

TABLE OF CONTENTS

ATN 129 PROTOCOL TEAM ROSTER 5
REQUIREMENTS FOR SITE PARTICIPATION 7
STUDY MANAGEMENT 8
LIST OF ABBREVIATIONS 9
STUDY ABSTRACT 11
PROTOCOL DESIGN OR PROTOCOL SCHEMA 14
1.0 INTRODUCTION 15
2.0 SIGNIFICANCE 16
3.0 STUDY OBJECTIVES 17
3.1 Primary Objective 16
3.2 Secondary Objectives 16
4.0 PROTOCOL REGISTRATION 18
5.0 SELECTION AND ENROLLMENT OF STUDY PARTICIPANTS 18
5.1 Inclusion Criteria 18
5.2 Exclusion Criteria 19
5.3 Recruitment and Screening 19
5.4 Informed Consent 20
5.5 Contact Information 20
6.0 STUDY PROCEDURES 20
6.1 Enrollment Procedures 20
6.2 Randomization Procedures 21
7.0 INTERVENTION PROCEDURES 22
7.1 Clinic-Based and Home-Based Conditions: Healthy Choices – MET for YLH 22
7.2 Four Sessions of MET 23
7.3 CHW and SUPERVISOR MET Training 23
7.4 Intervention Monitoring/Quality Control 23
8.0 EVALUATIONS AND MEASURES 24
8.1 Pre-Entry Evaluations and Measures 24
8.2 Study Evaluations and Measures (Post-Enrollment) 24

8.3 Process and Cost Effectiveness Measures ... 26
8.4 Intervention and Study Evaluations ... 27
8.5 Overview of Study Evaluations and Measures by Administration ... 27
9.0 DATA COLLECTION AND SITE MONITORING ... 28
9.1 Development of Protocol and Case Report Forms ... 28
9.2 Data Records ... 28
9.3 Data Collection ... 29
9.4 Data Submission ... 30
9.5 Data Quality Assurance ... 31
9.6 Role of Data Management ... 31
9.7 Study Site Monitoring and Record Availability ... 32
10.0 PARTICIPANT MANAGEMENT ... 32
10.1 Tracking Participants / Follow-up ... 32
10.2 Study Visit Management ... 32
10.3 Compensation ... 33
10.4 Intervening on “Social Harms" ... 33
10.5 Criteria for Premature Discontinuation ... 33
11.0 MONITORING UNTOWARD EVENTS ... 34
11.1 Study Data Safety & Monitoring Board Members ... 34
12.0 STATISTICAL/ANALYTIC CONSIDERATIONS ... 35
12.1 Sample Size and Power Estimates ... 35
12.2 Statistical Analysis Plan ... 35
12.3 Missing, Unused and Spurious Data ... 37
13.0 HUMAN PARTICIPANT ... 37
13.1 Participants’ Confidentiality ... 37
13.2 Certificate of Confidentiality ... 38
13.3 Risks and Benefits ... 38
13.4 Institutional Review Board (IRB) Review and Informed Consent ... 38
13.5 Waiver of the Requirement for Parental Permission for Special Circumstances ... 39
13.6 Prisoner Participation ... 39
13.7 45 CFR Parts 160 and 164 Standards for Privacy of Individually Identifiable Health Information ("Privacy Rule" Pursuant to the Health Insurance Portability and Accountability Act - HIPAA) ... 39
13.8 Study Discontinuation ... 40
14.0 PUBLICATION OF RESEARCH FINDINGS ... 40

15.0 REFERENCES.. 41
APPENDICES 49

ATN 129 PROTOCOL TEAM ROSTER

Protocol Co-Chair
[Principle Investigator]
Sylvie Naar-King, PhD
Wayne State University
4707 St. Antoine,
W534, Old Hutzel
Detroit MI, 48201
Phone: 248-207-2903
Fax: 313-745-4993
E-mail: snaarkin@med.wayne.edu

Protocol Co-Chair
[Co-Investigator; Project Director]
Phebe K. Lam, PhD
Wayne State University
4707 St. Antoine,
W534, Old Hutzel
Detroit MI, 48201
Phone: 519-817-8871
Fax: 313-745-4993
E-mail: plam@med.wayne.edu

NICHD Health Science Administrator
Sonia S. Lee, Ph.D.
PAMAB/CRMC/NICHD/NIH
Executive Building, Rm. 4B11K
6100 Executive Blvd, MSC 7510
Rockville, MD 20852
Phone: (301) 594-4783
Fax: (301) 496-8678
E-mail: leesonia@mail.nih.edu

NICHD Program Scientific Director
Bill G. Kapogiannis, MD
PAMAB/CRMC/NICHD/NIH
6100 Executive Boulevard, Room 4B11J
Bethesda, MD 20892-7510
Phone: (301) 402-0698
Fax: (301) 496-8678
E-mail: kapogiannisb@mail.nih.edu

Protocol Team Member
[Co-Investigator; Statistician; Analyst]
Xinguang (Jim) Chen, MD, PhD
College of Public Health & Health Professions and College of Medicine
University of Florida
2004 Mowry, Gainesville, FL 32610
Phone: (352) 293 5945
Fax: (352) 273 5365
E-mail: *Jimax.chen@phhp.ufl.edu*

Protocol Team Member
[Co-Investigator]
Jeffrey T. Parsons, PhD
Hunter College and the Graduate Center of the City University of New York (CUNY)
695 Park Avenue, Room 611N
New York, NY 10065
Phone: (212) 772-5533 Hunter office
(212) 206-7919 x900 CHEST office
Fax: (212) 206-7994 CHEST
Email: jeffrey.parsons@hunter.cuny.edu or jparsons@chestnyc.org

Protocol Team Member
[Co-Investigator]
Sharon Nichols, PhD
University of California, San Diego
9500 Gilman Drive, #0935
La Jolla, CA 92093
Phone: 858-822-6700
Fax: 858-822-6707
E-mail: slnichols@ucsd.edu

Protocol Team Member
[Co-Investigator]
Tyrel Starks, PhD
Center for HIV Education and Training (CHEST)
142 W. 36th St. 9th Floor
New York, NY 10018
Phone: 618 534 2698
Fax: 212 206 7994
Email: tstarks@chestnyc.org

Protocol Team Member
[Co-Investigator]
Deborah Ellis, PhD
Wayne State University
4707 St. Antoine,
W534, Old Hutzel
Detroit MI, 48201
Phone: 313-993-7851
Fax: 313-745-4993
E-mail: dellis@med.wayne.edu

Protocol Team Member
[Co-Investigator; Data Manager; Junior Statistician]
Karen MacDonell, PhD
Wayne State University
4707 St. Antoine,
W534, Old Hutzel
Detroit MI, 48201
Phone: 313-577-6996 (cell 773-220-7280)
Fax: 313-745-4993
E-mail: kkolmodin@med.wayne.edu

Protocol Team Member
Juline Koken, PhD
Center for HIV Education and Training (CHEST)
142 W.36th St., 9th Floor
New York, NY 10018
Phone: 212-206-7919 ext. 927
Fax: 212 206 7994
Email: jkoken@chestnyc.org

Protocol Specialist (Consultant)
Dina Monte, RN, BSN, CCRC
Westat, WB 420S
1600 Research Boulevard
Rockville, MD 20850
Phone: (914) 591-6170
Fax: (914) 591-8030
E-mail: dinamonte@westat.com

ATN Site PI Representative
Aditya H. Gaur, MD
St. Jude Children's Research Hospital
262 Danny Thomas Place
Memphis, TN 38105
Phone: (901) 595-2862
Fax: (901) 595-5068
E-mail: aditya.gaur@stjude.org

ATN Site Study Coordinator Representative
Rachel M. Jackson, APN, CFNP
The CORE Center
2020 W. Harrison
Chicago, IL. 60612
Phone: (312) 572-4554
Fax: (312) 572-4559
Email: rjackson@cookcountyhhs.org

REQUIREMENTS FOR SITE PARTICIPATION

This study is open to five Adolescent Medicine Trial Units (AMTUs) of the Adolescent Trials Network (ATN). Each AMTU will meet the following minimum requirements in order to participate:

Site Characteristics

1) Ability to recruit, over a two-year period, 100 youth with detectable HIV viral load (HIV-VL) and any alcohol use in the last 12 weeks.
2) Have a Community Health Worker (CHW; i.e., outreach worker, advocate, case manager, health educator, navigator, or other paraprofessional staff) who can dedicate 20% full-time equivalent (FTE) for three years and be willing to do home- and clinic-based work. Position starting May 1, 2014.
3) Have a supervisor (i.e., social worker, psychologist) for the Community Health Worker (CHW-S) 5% FTE for 3 years. Position starting May 1, 2014.
4) Ability to run the study with a Research Assistant (RA) 50% FTE. , and a start date of approximately June 1, 2014.
5) Ability to run the study with a Study Coordinator (SC) 5% FTE for 3 years. Position starting April 1, 2014.
6) Ability to submit IRB application to their institution by April 2014.
7) Have the selected study CHW and CHW-S attend the Motivational Interviewing (MI) training May 13 to 15, 2014 in Detroit Michigan (3 full days of training).
8) Have wireless Internet capability.

STUDY MANAGEMENT

Before the recruitment and enrollment of participants, the participating ATN study sites must have the protocol and consent form(s) (and assents) approved by their local Institutional Review Boards (IRBs). In addition, ATN study sites must submit for protocol registration from the ATN Data and Operations Center (DOC). All original, approved documents must be maintained at Wayne State University (WSU) and at the ATN study site. A detailed description of site and protocol registration procedures is included in Chapter 6 of the ATN Manual of General Operations (MOGO).

All sites will follow the MOGO for general ATN study procedures, if there is any doubt or inconsistencies between the MOGO and WSU procedures as stated in this protocol, please send a query to the Protocol Team. All queries for this protocol should be sent to the ATN 129 protocol team using the ATN Protocol Query and Notification System (QNS) accessible via the ATN website (https://www.atnonline.org). The appropriate team member will respond to queries generally within 48 business hours via the ATN QNS and copy the other team members. The Protocol Co-Chairs or their designee will answer general protocol implementation, eligibility, and Case Report Form (CRF) completion. The Protocol Co-Chairs or their designee will respond to study and participant management, exemptions and/or adverse event queries. This study follows the ATN Policy for Guidance for Safety and Impact Reporting located in Appendix I-B of the ATN MOGO. Queries and replies will automatically be archived at the ATN DOC. The Protocol Specialist will post those queries deemed relevant to all sites on the ATN website, where they will be available for future reference.

Concurrent or past participation in neurocognitive research is not an ATN 129 exclusion criteria, although AMTU staff must inform the ATN 129 Protocol Team through the query system. Also, concurrent or past participant in behavioral research (assessment or intervention) targeting adherence or alcohol use OR current involvement in a substance abuse treatment program are allowed and are not study exclusion criteria. Participation in any other ATN protocol whether it's neurocognitive or behavioral research, as with current involvement in a substance abuse treatment program will be documented in the Audio Computer-Assisted Self-Interview (ACASI).

This study will use the ACASI and an interview with a RA (Timeline Follow Back) on a laptop, as well as computerized and paper-and-pencil neurocognitive assessments with a RA to collect study data. All questions related to the ACASI should be directed to the Qualtrics Technical Help Desk AND Dr. Phebe Lam AND Dr. Karen MacDonell. The Qualtrics Technical Help Desk can be contacted either by calling the toll-free Qualtrics helpline at 1-(800)-340-9194 or by e-mailing support@qualtrics.com. The Qualtrics Help Desk at WSU will be available to provide technical assistance to sites. Please remember to always keep Dr. Phebe Lam (plam@med.wayne.edu) and Dr. Karen MacDonell (kkolmodin@med.wayne.edu) on all communications.

All information collected outside of ACASI or Qualtrics program must be documented on study CRF forms (ex, Screener and Eligibility Form, Virology Results Form, Nail Sample Form, etc.), entered into Qualtrics, and uploaded to its designated folder in Dropbox, our secure data sharing website within the allotted time frame of completion.

For protocol registration issues, contact the ATN Regulatory Affairs Office at Regulatory@westat.com.

LIST OF ABBREVIATIONS

ACASI	Audio Computer Assisted Self Interview
ADAP	AIDS Drug Assistance Programs
AIDS	Acquired Immunodeficiency Syndrome
AMTU	Adolescent Medicine Trials Unit
ART	Antiretroviral Therapy
ATN	Adolescent Medicine Trials Network for HIV/AIDS Interventions
BLG	Behavioral Leadership Group
CER	Comparative Effectiveness Research
CFR	Code of Federal Regulations
CHW	Community Health Worker
CHW-S	Community Health Worker Supervisor
CRF	Case Report Form
DATCAP	Drug Abuse Treatment Cost Analysis Program
DCF	Data Collection Form
DHHS	U.S. Department of Health and Human Services
DNA	Deoxyribonucleic Acid
DOC	Data and Operations Center
DSMB	Data Safety and Monitoring Board
EBP	Evidence Based Practice
EBPAS	Evidence Based Practice Attitudes Scale
EC	Ethics Committee
FDA	Food and Drug Administration
FTE	Full-Time Equivalent
GCP	Good Clinical Practices
GMM	Growth Mixture Modeling
HC	Healthy Choices
HIPAA	Health Insurance Portability and Accountability Act
HIV	Human Immunodeficiency Virus
HIV-VL	HIV Viral Load
HIV+	HIV Seropositive
HS	Health State
HVLT-R	Hopkins Verbal Learning Test-Revised
ICH	International Conference on Harmonization
IRB	Institutional Review Board
LES	Life Events Survey
MAR	Missing at Random
MET	Motivational Enhancement Therapy
MI	Motivational Interviewing
MIA-STEP	Motivational Interviewing Assessment – Supervisor Tools for Enhancing Proficiency
MITI	Motivational Interviewing Treatment Integrity
MOGO	Manual of General Operations
NEURO-QOL	Neurological Disorders Quality of Life
NIAAA	National Institute on Alcohol Abuse and Alcoholism
NICHD	National Institute of Child Health and Development
NIDA	National Institute on Drug Abuse
NIH	National Institutes of Health
NIMH	National Institute of Mental Health

NINDS	National Institute of Neurological Disorders and Stroke
OHRP	Office of Human Research Protection
PCR	Polymerase Chain Reaction
PHI	Protected Health Information
PI	Principal Investigator
PID	Patient Identification Number
PSQ	Patient Satisfaction Questionnaire
QNS	Query and Notification System
RA	Research Assistant
RNA	Ribonucleic Acid
SAMHSA	Substance Abuse and Mental Health Services Administration
SC	Study Coordinator
SID	Study Identification Number
SID	Study Identification Number
SOC	Service Organization Controls
SSAE-16	Statements on Standards for Attestation Engagements
TLFB	Timeline Follow Back
TLS	Transport Layer Security
VAS	Visual Analogue Scale (VAS)
WSU	Wayne State University
YLH	Youth Living with HIV

STUDY ABSTRACT

DESIGN:	This study will use a Type 1 Effectiveness-implementation hybrid design to pilot a sustainable model of motivational interviewing (MI) implementation in real-world youth care settings towards the goals of: 1) examining the effectiveness, cost-effectiveness, and scalability of an efficacious behavioral intervention when delivered by Community Health Workers (CHWs) in real-world youth HIV care settings; 2) gathering information about who responds to the intervention and under what contexts; and 3) increasing understanding of the study intervention related barriers and facilitators for future implementation.
SAMPLE SIZE:	Approximately 500 participants will be enrolled among five Adolescent Medicine trials Units (AMTUs), 100 per site (see Appendices or list of sites and their respective principal investigators [PIs]).
POPULATION:	HIV-positive youth who understand and speak English, are 16 through 24 years old at baseline data collection, have any alcohol use in the past 12 weeks **and** have been offered a prescription for antiretroviral (ARV) medications (even if they refuse or did not fill prescription) **and** have detectable HIV viral load (HIV-VL). Youth will be recruited from five Adolescent Trials Network (ATN) sites:<br>1) Site 02 – Los Angeles-Children's Hospital Los Angeles<br>2) Site 04 – Philadelphia-Children's Hospital of Philadelphia<br>3) Site 05 – Chicago-Stroger Hospital and the Core Center<br>4) Site 16 – Memphis-St. Jude Children's Research Hospital<br>5) Site 19 – Detroit-Wayne State University
INTERVENTION:	This study will compare the effectiveness of home- versus clinic-based Motivational Enhancement Therapy (MET) "Healthy Choices" (HC), a brief MI designed to address alcohol use, ARV adherence, and health outcomes in youth living with HIV (YLH). Unlike past studies, Healthy Choices will be delivered by community health workers (CHWs), who are already members of the HIV care team at each site. The intervention consists of four sessions over a 12-week timeframe. **For home-based MET, this can also be at any mutually agreed upon community venue other than the participant's home, but hereinafter will be referred to as "home-based"**. For the home-based youths, if they are at the clinic for AMTU services, and the youth would like to meet their CHW at the clinic for their MET session, they can do so. However, if the home-based youth is only at the clinic for a research visit (data collection), they CANNOT meet the CHW at the clinic for a MET session.

RANDOMIZATION (STRATIFICATION):	Participants will be randomized to receive either clinic-based or home-based Healthy Choices (HC) sessions (1:1 within each site). At each site, the same CHW will deliver the intervention to both groups.
DURATION:	Each enrolled youth will participate for a total of approximately 52 weeks.<br>• Baseline Data Collection<br>• Intervention<br>o Four sessions of HC over 12 weeks delivered by CHWs either at the clinic or in the home (or a mutually agreed upon community venue)<br>• Follow Up Data Collection (post baseline):<br>o 16 weeks<br>o 28 weeks<br>o 52 weeks
PRIMARY OBJECTIVES:	**To compare the effectiveness of home-based versus clinic-based delivery of MET in real-world clinical settings by indigenous CHWs.**<br>The primary goal is to test whether CHW home-based delivery of MET HC adapted for YLH decreases alcohol use and HIV viral load relative to CHW clinic-based delivery.
SECONDARY OBJECTIVES:	1. **To compare the cost-effectiveness of home-based versus clinic-based delivery of MET**. In order to enhance the likelihood of uptake, a cost-effectiveness analysis will be conducted on the two delivery formats.<br>2. **To determine individual, interpersonal, and environmental factors that moderate effectiveness of MET.** Using a social ecological framework, individual (level of baseline drinking, mental health, neurocognitive functioning), interpersonal (disclosure, social support, social norms), and environmental (stigma, life events, employment, income, insurance status, housing, and geocoding of alcohol environment, and neighborhood disadvantage) factors will be assessed, as well as patient preferences, that moderate effectiveness of MET and determine who responds to home-based versus clinic-based delivery.<br>3. **To pilot a sustainable model of MET implementation in real world adolescent HIV care settings.** Local supervisors within the clinic setting will be utilized to sustain the CHW's MI fidelity. We will monitor fidelity throughout the trial and beyond the intervention period and conduct qualitative interviews with key stakeholders to determine feasibility, acceptability, sustainability, and barriers related to implementing MET. In this way, the project is a Type 1 Effectiveness-implementation hybrid design where the primary aim is to compare the effectiveness of two clinical interventions (home-based

	versus clinic-based MET) and a secondary aim is to evaluate the context for implementation.[41]
DATA COLLECTION:	All measures are administered at baseline, 16 weeks, 28 weeks and 52 weeks All baseline assessments should be completed preferably within the same day, and if not, then no later than 7 days after the beginning of the baseline. All follow-up data collections (16-, 28-, and 52-weeks) can be up to two weeks prior to their due date and up to four weeks post their due date.<br>Data collection for biological measures for HIV-VL and alcohol use will be conducted through medical record extraction and/or blood draw (HIV-VL), and finger/toe nail samples (alcohol use; NailStat). Questionnaire measures will be conducted through three mechanisms: Research Assistant (RA) Interview-Timeline Follow Back (TLFB) on a laptop, self-reported measures will occur using a Web-based ACASI survey on a laptop, and neurocognitive measures on a laptop and by paper-and-pencil format with a RA.<br>All intervention sessions will be digitally recorded for Motivational Interviewing Treatment Integrity (MITI) fidelity coding, and investigators will support local supervisors during the active intervention phase.<br>Qualitative interviews with CHWs, CHW-Ss, site staff (SC, RA, PIs) will be conducted post-training and at the end of the study to obtain critical information about barriers and facilitators encountered during implementation. The interviewer will be a member of the research Protocol Team at WSU.
MONITORING:	Routine team monitoring of events identified during the study will rely on site staff notification via the ATN Protocol Query & Notification System (QNS), a real-time, web-based interactive reporting system. Sites will also record it on the *ATN 129 Monitoring Untoward Events Form* and enter in the study database, untoward events occurring during study participation, which will be reviewed during the protocol team's implementation monitoring calls. The current study has an external Data Safety and Monitoring Board (DSMB) outside of the ATN.

PROTOCOL DESIGN OR PROTOCOL SCHEMA

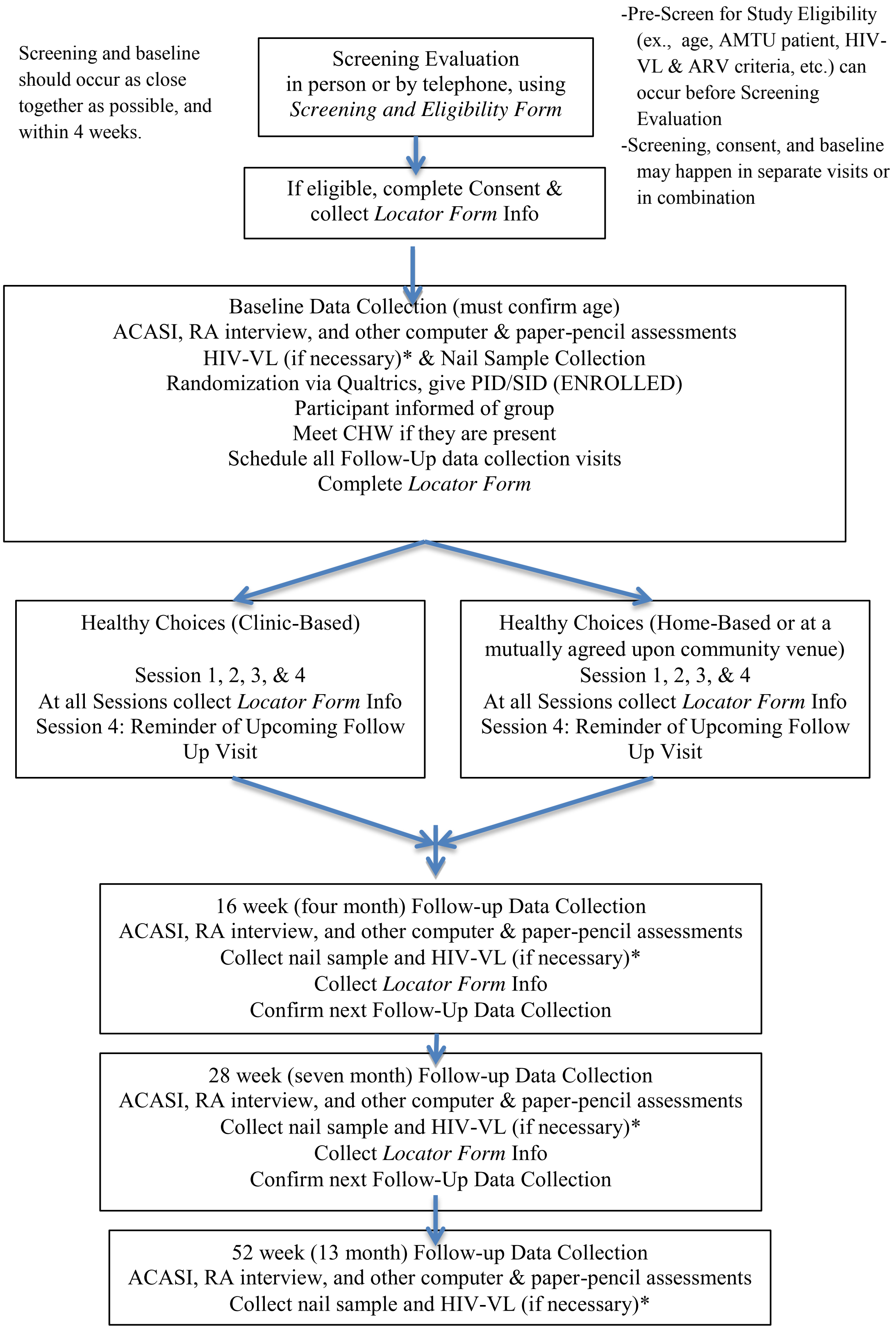

*HIV-VL will need to be drawn for the study if there is no HIV-VL drawn within 4 weeks of Screening, Baseline, or any Follow Up data collections, as verified by chart review.

1.0 INTRODUCTION

Alcohol consumption among HIV seropositive (HIV+) persons exacerbates health problems and accelerates HIV disease progression.[1-7] Antiretroviral therapy (ART) is the single most important treatment for HIV+ persons because ART optimizes viral suppression and slows disease progression.[8-11] Optimal adherence decreases morbidity and mortality,[1,2,11-13] the potential for the development of drug resistant strains of HIV,[14-16] and HIV infectiousness.[17-20] However, ART adherence is suboptimal among youth living with HIV (YLH),[21,22] the age group with the fastest growing rate of HIV infection.[23] Adolescence and emerging adulthood are the developmental periods when risky behaviors such as alcohol use peak,[24] and alcohol is a major factor contributing to poor adherence in adults[25] and YLH.[21]

Brief interventions are recommended as developmentally appropriate for targeting alcohol use in youth[26] and Motivational Enhancement Therapy (MET) is a leading brief, effective alcohol intervention in SAMHSA's registry of evidenced-based programs and practices. This 4-session, 12-week intervention was adapted to target multiple behaviors, including ART adherence and substance use, in YLH in the *Healthy Choices* trial.[27] This adaptation of MET is the only intervention (to our knowledge) to demonstrate improvements in HIV viral load and reduction in alcohol use in YLH in a full-scale, multi-site randomized trial.[28,29] *Healthy Choices* resulted in lower levels of alcohol use in YLH over 15 months of follow-up. Furthermore, trajectory analysis revealed that the intervention increased the likelihood that YLH would be characterized in a low-risk alcohol use group versus a moderate- or high-risk alcohol use group. We now plan to compare the effectiveness and cost-effectiveness of the *Healthy Choices* adaptation of MET using different service delivery methods to promote dissemination on a wider scale.[30-34] This strategy has been identified as being key to advancing our scientific understanding of behavioral interventions in HIV/AIDS.[31]

Healthy Choices was a 5-year RCT through the ATN. Two hundred and five YLH (ages 16 to 24) with at least one problem behavior (adherence, alcohol/drug use, or sexual risk) were randomized to receive 4 sessions of clinic-based MET (weeks 1, 2, 6, 10) plus multidisciplinary HIV care or multidisciplinary HIV care and referrals. Staff (not CHWs) in 5 sites were trained in MI with ongoing fidelity monitoring using the Motivational Interviewing Treatment Integrity (MITI)[96] codes. Retention rates were over 85% for all time-points consistent with other ATN studies. Immediately post-intervention, YLH receiving MET reported significantly greater reductions in depression.[35] At 15-month follow-up, past week alcohol use was significantly lower for intervention youth than control youth (39.7% versus 53.6%) across the whole sample. Figure 1 shows developmental trajectory analysis for alcohol use. The intervention was also associated with reductions in marijuana use for those in the low and moderate-risk trajectories.

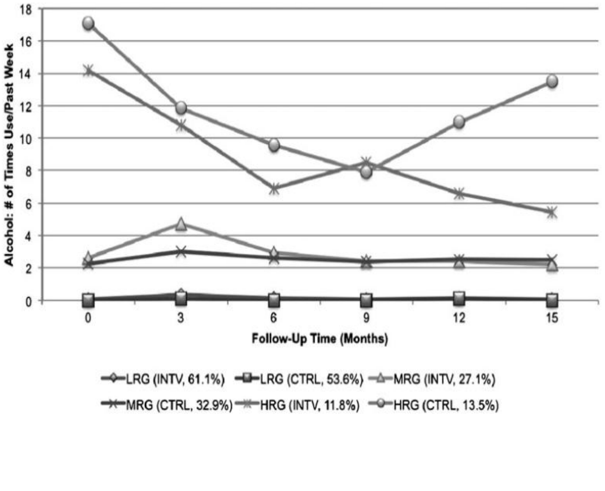

Trajectory analysis revealed that the intervention increased the likelihood that YLH would be characterized in the low risk group versus the moderate-risk group (2 or more drinks in a week) or high-risk group (8 or more drinks in a week). Even among the highest use groups, participation in MET predicted declining trajectory of use across time. Multiple linear regression models assessed the effect of the intervention controlling for differences in viral load at baseline and other covariates and showed that the MET group had significantly greater viral load drop at 6 months (β = -0.36, t = -2.15, df=1, p = 0.03), with youth on ART experiencing a one log drop in viral load, but effects were not maintained at 9 months.[35]

.

In the original *Healthy Choices* trial, 84% of the intervention group attended at least 1 session, only 49% attended all 4 sessions.[35] Thus, we do not know if a full intervention dose would yield greater and more sustainable improvements, and by delivering the intervention in the home we may remove barriers to access. Although results suggest that MET is effective, particularly for alcohol, and is ready for a comparative effectiveness trial, the study was not powered to look at the effects of moderators explaining variability in intervention response. Importantly, the proposed trial would allow an understanding of who responds under which circumstances. Many have suggested home-based service delivery to increase access to behavioral health services, but this remains untested in YLH.[36,37] Thus, a head to head comparison of two delivery formats of an effective intervention, MET home-based versus clinic-based and testing the factors within a social ecological model[38] that moderate this effect, will yield critical information about which intervention delivery is most effective (in terms of dose received and overall outcomes) for which young patients under specific circumstances. By utilizing indigenous community health workers (CHWs; e.g., outreach workers, case managers, health educators, health navigators) who already provide clinic-based and home-based services and providing support for local supervisors to promote fidelity, we may identify accurate, reproducible, and affordable methods for intervening with YLH to improve alcohol use, adherence, and related health outcomes.

We have conducted multiple trials utilizing CHWs to deliver behavioral interventions. In a federally funded study (H97HA03785A0, PI Outlaw), young MSM of color were randomized to receive a single session of field outreach with MI compared to traditional field outreach with the goal of promoting HIV counseling and testing. Providers in both groups were CHWs. More youth in the MI condition than the control condition received HIV counseling and testing (49% vs. 20%; (χ^2 (1, N = 188) = 17.94, p =.00) and returned for test results (98% vs. 72%; (χ^2 (1, N = 65) = 10.22, p =.00). Also, we published a book chapter focusing on using CHWs to successfully implement community-based behavioral interventions[98]. In another federally funded study (PI, Naar-King), YLH were randomized to two sessions of MI to promote retention in HIV care: one group received MI from CHWs and the second group from Master's level staff. Results indicated that both groups improved retention in HIV care, though the effect size and intervention retention rate was higher for CHWs.[99] While MI fidelity was high for both groups, CHWs had significantly higher scores on two scales of the MITI fidelity codes. Dr. Naar-King, along with Dr. Ellis, is also currently funded to train CHWs to deliver MI plus behavioral skills to African American youth with obesity as part of her NHLBI center grant, and has provided MI training as part of a linkage to care protocol.[100]

2.0 SIGNIFICANCE

2.1 Alcohol use impacts HIV disease progression and other health outcomes. Multiple studies have identified a relationship between alcohol use and accelerated HIV disease.[1-7,42-44] Even two drinks per day has been associated longitudinally with accelerated HIV disease progression and a decline in cluster of differentiation 4 (CD4) count.[3] Evidence has also shown that alcohol use increases CYP3A4 activity,[45] such that HIV+ persons on ART metabolized by CYP3A4 may experience reductions in therapeutic drug levels.[46] However, it has also been shown that these negative effects can be reversed following a two-week period of alcohol abstinence.[45] Alcohol use has also been identified as a risk factor for pancreatitis,[47] bone marrow toxicity, and hematopoietic impairment among those on ART,[48] which is of particular concern considering HCV liver disease and that HCV and HIV co-infection is common.[1,49-52] Finally, there is increasing concern about the detrimental effects of HIV infection and alcohol use on brain functioning,[53] and abstinence may reverse these effects[80,81].

2.2 Alcohol use decreases ART adherence, increasing the risk of HIV transmission. A recent meta-analysis of 40 studies found that drinkers were half as likely to be ART adherent compared to those who were abstinent or who consumed alcohol infrequently.[11] While those with alcohol use disorders are at highest risk,[11] even recreational alcohol use has been shown to be associated with both non-adherence as well as poor virologic and immunologic outcomes, independent of its effect on adherence.[4] A multisite study of adherence in YLH also demonstrated that less alcohol use was associated with better adherence[54]. Maintaining viral suppression through ART is a successful method of preventing HIV transmission to sexual partners,[17-20,55-57,58] Thus, reducing viral burden via improved ART adherence has **a double public health benefit**, both in terms of slowing disease progression and improving health outcomes among HIV+ individuals and in helping to curb the sexual transmission of HIV.[1,11,55] Reducing alcohol use

also has **a double public health benefit** because of its relationship with risky sexual behavior[59] and non-adherence to ART in YLH.

2.3 MET has the potential to reduce alcohol use and viral load among YLH, a vulnerable and understudied subpopulation of HIV+ persons, if implemented widely in clinics. Adolescents and young adults represent almost half of new HIV infections.[60] During this developmental period, risk behaviors peak[24] and ART adherence is notoriously poor.[21] Yet, few published behavioral interventions have targeted YLH. MET is a 4-session adaptation of Motivational Interviewing (MI)[61] that is widely disseminated to target alcohol and other drug use. MET was adapted to target multiple health risk behaviors specifically in YLH in our *Healthy Choices* trial. It is the only intervention (to our knowledge) to demonstrate improvements in viral load and to improve alcohol use in YLH in a full-scale, multi-site randomized trial.[28,29] Not only did MET result in lower levels of alcohol use over 15 months of follow-up across the whole sample, but trajectory analysis revealed that the intervention increased the likelihood that YLH would be characterized in the low-risk trajectory group versus the moderate-risk group (2 or more drinks in a week) or high-risk group (8 or more drinks in a week) trajectories. Even among the highest use groups, participation in MET predicted declining trajectory of use across time. MET produced significant improvements in viral load at 6-month follow-up. These improvements were not maintained at 9 months; however, the durability of treatment effects associated with viral load may have been a function of treatment dose. While 84% of the intervention group attended at least 1 intervention session, only 49% attended all 4 sessions. The current study will enhance understanding of the relationship between dose and durability of MET treatment effects. The *Healthy Choices* trial suggested clinic-based delivery could represent a barrier to intervention session attendance by requiring a greater frequency of clinic visits than typical for the standard care of YLH (four visits in three months). Thus, MET should be studied in non-clinic based settings. Although outreach models are highly recommended for marginalized populations including YLH,[62,63] very few outreach models include the provision of mental health or substance abuse services outside of the clinic setting. In a review of the diffusion of evidence-based behavioral interventions for HIV, home-based services are recommended.[64] HIV testing rates are much higher when provided home-based.[65,66] In a study in South Africa, home-based HIV programs have significantly higher follow-up and retention rates, over 85% compared to about 70% in clinic-based programs.[64] The potentially higher costs of home-based delivery may be offset by the benefits in intervention reach and engagement. To date, such comparisons have not been tested in the U.S. or with YLH who may have additional concerns about stigma or barriers to accessing mental health/substance abuse services such as low motivation, unstable housing, or competing interests. Thus, comparative effectiveness research (CER) that focuses on a head-to-head comparison of clinic-based versus home-based delivery of MET for YLH in "real world" clinical settings is a significant next step in improving health outcomes for this vulnerable group.

3.0 STUDY OBJECTIVES

We will conduct this study within the Adolescent Trials Network for HIV/AIDS (ATN), an NIH research network that partners with 14 clinical sites. Thus, we are able to test the intervention in real world clinical settings while capitalizing on a strong research infrastructure with a long history of collaboration among the investigative team. We will achieve our goals through four specific objectives:

3.1 Primary Objective

3.1.1 To compare the effectiveness of home-based versus clinic-based delivery of MET in real-world clinical settings by indigenous CHWs. The primary goal is to test whether CHW home-based delivery of MET HC adapted for YLH decreases alcohol use and HIV viral load relative to CHW clinic-based delivery.

3.2 Secondary Objectives:

3.2.1 To compare the cost-effectiveness of home-based versus clinic-based delivery of MET. In order to enhance the likelihood of uptake, a cost-effectiveness analysis will be conducted on the two delivery formats.

3.2.2 To determine individual, interpersonal, and environmental factors that moderate effectiveness. Using a social ecological framework, individual (level of baseline drinking, mental health, neurocognitive functioning), interpersonal (disclosure, social support, social norms), and environmental (stigma, life events, employment, income, insurance status, housing, and geocoding of alcohol environment, and neighborhood disadvantage) factors will be assessed, as well as patient preferences, that moderate effectiveness of MET and determine who responds to home-based versus clinic-based delivery.

3.2.3 To pilot a sustainable model of MET implementation in real world adolescent HIV care settings. Local supervisors within the clinic setting will be utilized to sustain the CHW's MI fidelity. We will monitor fidelity throughout the trial and beyond the intervention period and conduct qualitative interviews with key stakeholders to determine feasibility, acceptability, sustainability, and barriers related to implementing MET. In this way, the project is a Type 1 Effectiveness-implementation hybrid design where the primary aim is to compare the effectiveness of two clinical interventions (home-based versus clinic-based MET) and a secondary aim is to evaluate the context for implementation.[41]

4.0 PROTOCOL REGISTRATION

Before the enrollment of participant, the participating site must have the protocol approved by its local Institutional Review Board (IRB) or Ethics Committee (EC). The site must then register the protocol with the Regulatory Office at the ATN Data and Operations Center (DOC) by completing the protocol registration checklist and submitting copies of all required regulatory documents (See Chapter 6 of the ATN Manual of General Operations (MOGO). Original documents must be maintained at the clinical research site.

The ATN DOC will approve the required documents only after reviewing them to ensure that they are in compliance with all ATN requirements (See Chapter 5 of the ATN MOGO), and federal and local IRB/EC regulations.

5.0 SELECTION AND ENROLLMENT OF STUDY PARTICIPANTS

5.1 Inclusion Criteria

To be considered eligible for enrollment, an individual must meet the criteria listed below.

5.1.1 Receives services at one of the selected AMTUs

5.1.2 HIV-1 infection as documented in the participant's medical record by at least one of the following criteria:

- Reactive HIV screening test result with an HIV antibody or HIV antibody/antigen-based, Food and Drug Administration (FDA)-licensed assay followed by a positive supplemental assay (e.g., HIV-1 Western Blot, HIV-1 indirect immunofluorescence, HIV-1/HIV-2 discriminatory immunoassay);
- Positive HIV-1 deoxyribonucleic acid (DNA) polymerase chain reaction (PCR) assay;
- Plasma HIV-1 quantitative ribonucleic acid (RNA) assay >1,000 copies/mL; or
- Positive plasma HIV-1 RNA qualitative assay

5.1.3 Youth between the ages of 16 years and 0 days and 24 years and 364 days at the time of baseline data collection (must be confirmed due to possibility of a youth aging out if the screener, consent, and baseline data collection are not completed in the same day).

5.1.4 Detectable plasma HIV-VL using an approved latest generation test and within 4 weeks at screening time.

5.1.5. Have been offered a prescription for antiretroviral (ARV) medications (youth are still eligible for the study even if they refused to take prescription/medication or did not fill prescription).

5.1.6 Any alcohol use in the last 12 weeks prior to screening, even if it is just one sip of alcohol (ex. wine, beer, wine coolers, liquor, jello shots, medication with alcohol (cold or cough) to gain the effects of using alcohol, etc.)?

5.1.7 Not intending to relocate out of the current geographical area for the duration of study participation.

5.1.8 Signed parental permission with participant assent for participants less than 18 years old if a waiver of parental permission was not granted by the site's IRB.

5.1.9 Able to understand, read, and speak English sufficiently to provide informed consent/assent and be interviewed and participate in study intervention in English.

5.1.10 Willingness to provide signed informed consent.

5.2 Exclusion Criteria

To be considered eligible for enrollment, an individual must not meet any of the criteria listed below.

5.2.1 Any thought disorder (psychosis) or intellectual disability that would impair the individual's ability to provide true informed consent/assent and/or interfere with the protocol's objectives.

*** **NOTE:** Co-morbidities such as mental health problems, i.e., ADHD, conduct disorder, depression, and anxiety disorder are NOT considered exclusion criteria. Additionally, current or previous participation in behavioral or neurocognitive studies or interventions is NOT considered an exclusion criterion.

5.2.2 Intoxicated or under the influence of alcohol or other substances at the time of consent/assent that would impair the individual's ability to provide true informed consent/assent and/or interfere with the protocol's objectives.

5.3 Recruitment and Screening

Potential participants will be approached for the study through the clinics by trained clinic/research staff, AMTU staff or other means, as applicable. They will be informed of the nature of the study, the information to be collected, and the evaluations and assessments that are involved. Individuals who express interest in the study will be required to complete a screening and eligibility assessment, and have eligibility criteria confirmed by the research/AMTU staff. There are no limits on the number of times an individual can be re-screened after failing a screening assessment.

Each individual approached for recruitment into ATN 129, will have the following information recorded on an *ATN 129 Study Screening Log* (see Appendices), which will be maintained in a secure area at the AMTU with access available to study staff only: Name or initials, date of birth, age when considered for enrollment, birth gender, race and ethnicity. Individuals who are either not consented or consented, but not enrolled, will have reasons recorded. Data on the screening log will be entered into the ATN 129 database through Qualtrics, except names/initials and dates of birth, by the site research staff. The screening log will allow site staff to track individuals approached for study enrollment and gather information on reasons why consent was not obtained or if it was obtained, why enrollment did not occur. This screening log will uploaded into its specific folder in Dropbox every 18 days. Once study accrual ceases, each site must obliterate the names/initials and dates of birth of all individuals that did not provide consent to participate; thereby removing any link to those personal identities. Overall, this data will allow the protocol team to report on recruitment practices at the sites, assess obstacles that may be preventing individuals from consenting and gather information on reasons why individuals that consent may be deemed ineligible.

5.4 Informed Consent

Once it is determined that the individual is eligible for the protocol, study details will be discussed and all questions answered during the informed consent process. Signed informed consent from the individuals or assent with waiver of parental permission as determined by local Institutional Review Boards (IRB) will be obtained before any study related procedures are performed (See Appendices VI). Once consent/assent is obtained, the participant can complete their baseline data collection. Screening, consent, and baseline should occur as close together as possible, and within four weeks. If greater than four weeks occur, then the participant will need to be rescreened for eligibility. Youth's age must be confirmed at the time of baseline data collection (must be confirmed due to possibility of a youth aging out if the screener, consent, and baseline data collection are not completed in the same day).

Those individuals who refuse to provide signed consent to participate in the study will be asked if they are willing to provide their reason for declining participation and if answered; the responses will be recorded in an anonymous manner on the *ATN 129 Screening and Eligibility Form* (see Appendices).

5.5 Contact Information

Once consented, designated site study staff will complete a *Locator Form* (see Appendices) with the participant. Participants will be asked to provide a home address and zip code, working phone number or valid email address through which they can be reached. Participants will also be asked to provide valid contact information for a family member and/or friend who can be called in the event the participant cannot be reached by phone or email. Participants will be asked if messages can be left at the numbers provided. Study staff will not leave messages unless expressly permitted to do so by the participant, which also will be documented on this form. If permission is given to leave messages, site staff will assure participants that messages left with a family member or friend will only ask the participant to contact study staff and will not include any protected health information or information related to study participation.

The *Locator Form* will be maintained under double locks at the study site, separate from all study records, with access limited to designated site research personnel. The *Locator Form* is completed every time a RA (data collections) or CHW (MET sessions) has contact with the participant.

A sample *ATN 129 Recruitment Script* is located in Appendices.

6.0 STUDY PROCEDURES

A Schedule of Evaluations (Appendices) provides a timeline of when each study activity/measure/etc. is to be performed.

6.1 Enrollment Procedures

A pre-screening of the participant of eligibility based on age, AMTU patient, HIV-VL and ARV criteria (through medical chart review) can be done before screening the participant by phone or in person. The information can be completed on the *ATN 129 Screening and Eligibility Form*. If deemed study eligible, the participant will be consented if they choose to participate in the study. After obtaining consent, the *ATN 129 Locator Form* will be completed to gather participant contact information. Baseline data collection can then be scheduled or the participant can complete baseline immediately if they choose to. Youth's age must be confirmed at the baseline data collection (must be confirmed due to possibility of a youth aging out if the screener, consent, and baseline data collection are not completed in the same day). If this timeframe goes past 4 weeks, then the participant will need to be rescreened. For every scheduled study visit, the ATN 129 *Visit Report Form* must be completed. Randomization via Qualtrics will occurs immediately after baseline data collection. If this is the first time an individual is participating in an ATN-sponsored study and he or she does not already have an ATN Patient Identification Number (PID), site staff will assign a PID in consecutive order from the pre-printed PID-Assignment Log that is provided by the ATN DOC and record the assignment in the Log. Participants with an existing PID number will continue to use the same number. Participants

who are confirmed eligible and enrolled will be assigned a protocol-specific SID from the SID list that is provided to the site by the Protocol Team. Each ATN site will use a different sequence of protocol numbers. SIDs will be organized as follows: Protocol Number, Site Number, Individual ID Number:

- 129 05 001
  - Protocol 129, Site 5 and Participant 1
- 129 04 099
  - Protocol 129, Site 4, Participant 99

Official enrollment on to this study will start when the participant is randomized and a SID (and PID if necessary) assigned to them. Their assigned PID and SID numbers are to be recorded on the *ATN 129 Screening and Eligibility Form* as well as other protocol-specific case report forms (CRF). All CRFs are to be uploaded into its specific folder in Dropbox and information entered into Qualtrics (if required) within 18 days of completion, except for the *ATN 129 Screening and Eligibility Form* and *ATN 129 Monitoring Untoward Events Form,* which must be loaded within 48 hours of event.

The site SC or RA will assign the PID and SID numbers, oversee study at their site, and facilitate recruitment and retention. AMTU staff (SC) will also maintain the list linking site participants to their assigned PID, SID, and linkage to CHWs. The linkage list will be stored separately from all data (i.e., digitial recordings, and completed measures). They will each be stored in a password protected folder. No individual data collected during the course of the study will be shared with the ATN sites.

Once enrolled, the RA will inform the participant of their study group (clinic- or home-based MET sessions). If the CHW is present at the clinic, then the participant can meet the CHW to schedule their first MET session if possible. Youths randomized to the clinic-based MET intervention can complete their first MET session with the CHW immediately after baseline data collection if the youth choose. For the home-based youths, if they are at the clinic for AMTU services, and the youth would like to meet their CHW at the clinic for their MET session, they can do so. However, if the home-based youth is only at the clinic for a research visit (data collection), they CANNOT meet the CHW at the clinic for a MET session. Finally, the RA should schedule all of the participants follow up data collections tentatively (16-, 28-, and 52-week time point).

Screening and eligibility check, consent, baseline data collection, and randomization should occur as close together as possible, and within four weeks.

6.2 Randomization Procedures

Qualtrics online software (http://qualtrics.com/) will be used to provide 24 hours, 7 day a week randomization. Qualtrics is a private research company providing software for many kinds of online data collection. Participants who are determined eligible at the completion of baseline will be randomized to one of two treatment conditions. Each site will receive a unique link to the Qualtrics survey so randomization can be completed at each site independently of other sites. The RA will log onto a computer with Internet connectivity and ask the participant (or enter the data if the data are known already) the following 13 questions.

ATN 129 Randomization Survey

The research assistant/study coordinator completes the information below during the baseline data collection. The information is also entered into Qualtrics for randomization.

1. What is the participant's SID number ________
2. How old is the participant? ________ (years)
3. What was the participant's assigned sex at birth? _______{male/female}

4. How does the participant self-identify? ____________ {gay/lesbian/bisexual; heterosexual}
5. Does the participant identify as Hispanic or Latino? ____ {yes/no}
6. What language does the participant speak at home? ______ {English, Other}
7. Which best describes the participant's racial identification? ____________ {White, non-white}
8. Does the participant live with a parent or other family member? _____ {yes/no}
9. How does the participant believe she/he was infected? ________ {pre/perinatally; injection drug use/sexual transmission}
10. How many days in the past 30 did the participant miss a dose of her/his medication? ____ {0-30 days; or indicate "new medication"}
11. How many days in the past 12 weeks did the participant have a drink containing alcohol, even a sip (ex. wine, beer, wine coolers, liquor, jello shots, medication with alcohol (cold or cough) to gain the effects of using alcohol, etc.)? ____ {0-12 weeks}
12. Did the participant use any substances (e.g., illegal or unprescribed prescription drugs) other than alcohol during the past 12 weeks? _____ {yes or no}
13. How long has the participant been prescribed their current ARV regimen? ________ {less than 12 weeks, 12 weeks or greater, or No Prescription}

Randomization status from Qualtrics (circle one): HOME CLINIC

Participant received randomization status: ________________(date)

After the Randomization Survey is completed, Qualtrics will display the treatment arm to which the participant is assigned. The SC will then record the treatment arm assignment into the appropriate fields on the Screener and Eligibility Form. The composition of participants assigned to each condition will be tracked by the study statistical team. The research team will adjust randomization specifications on Qualtrics if the covariates tracked in the Randomization Survey are found to be highly skewed at any study site.

7.0 INTERVENTION PROCEDURES

7.1 Clinic-Based and Home-Based Conditions: Healthy Choices – MET for YLH.

The four-session intervention is based on MET from Project Match[104] and MET to address substance use and HIV medication adherence among HIV+ men and women.[97,105] In the original Healthy Choices,[28,106,107] the basic MET session format was maintained, but youth could work on 2 of 3 possible problem behaviors (any alcohol or drug use, adherence, or sexual risk). Educational handouts were modified for YLH. Because normative behavioral data were not available for YLH, personalized feedback was delivered based on the baseline assessment. Youth will focus on alcohol use and ART adherence, though other behaviors such as sexual risk and other substance use may be addressed in the course of the sessions and referrals provided as necessary. Sessions will be delivered by a CHW (outreach worker,

advocate, case manager, or other paraprofessional) already providing services in the clinic. CHWs will be reimbursed for their travel to meet with their home-based youths.

7.2 Four Sessions of MET

In session 1, youth will choose which behavior to discuss first (alcohol or ART adherence), and the CHW will elicit the client's view of the problem using standard MI techniques, building motivation for change by eliciting and reinforcing change talk. The CHW will deliver feedback and discuss the consideration of a behavior change plan option, and the client sets the change plan goal. The session ends with MI strategies to consolidate commitment. The second session (week 2) follows the same format but targeting the second behavior. In the last two sessions (weeks 6 and 10), the CHW will review the change plan, continue to elicit and reinforce change talk, problem-solve barriers, consolidate commitment, and consider strategies to maintain behavior change.[108] After the last session, the CHW MAY be able to informally "booster" via regularly scheduled clinic visits. As this is an effectiveness trial, it is expected that CHWs could utilize the MI skills as part of clinical care (and their actual use of the skills will be assessed per the design). THE MET SESSIONS LAST APPROXIMATELY 60 MINUTES

7.2.1 Dose Timeline: Two MET sessions will be completed within weeks one through four, then one session from week five through eight, and one session from week nine through 12. All attempts to schedule/reschedule sessions, missed sessions, contacts (phone, text, email etc.) with participant, "drop by" home visits will be documented on the Contact Log by the CHW. The CHW-S will be responsible for maintaining these documents. Please note that only the CHW can provide the MET session to the youths, and the Protocol Team must be notified through the query system if a CHW is not available to provide sessions where it will affect the dose timeline. For the home-based youths, if they are at the clinic for AMTU services, and the youth would like to meet their CHW at the clinic for their MET session, they can do so (a mutually agreed upon community venue). However, if the home-based youth is only at the clinic for a research visit (data collection), they CANNOT meet the CHW at the clinic for a MET session.

7.2.2 No Show policy: The CHW will wait up to 15 minutes for a participant to show up for a MET session before considering the session a "no show". The CHW continues to call and text and attempt to reschedule per the dose timeline (two sessions in month one, week one through four; one session in month two, week five through eight; one session in month 3, week nine through 12). The CHW-S will provide oversight per clinic protocol. For home-based clients, after one missed session or "no show", if the CHW does not hear from the participant, they will make two drop bys to the participant's home. They will then call and text until the next visit. After any three "no shows" for home-based visits, the CHW will require the participant to confirm their appointment by phone or text before each scheduled session before the CHW attempts to visit the participant at their home.

7.3 CHW and SUPERVISOR MET Training

The MET training protocol will include: 1) initial 3-day training for CHWs and their local supervisors; 2) a 2-3 month training period of role-play practice, coding and feedback, and supervision modeling; 3) ongoing 1 hour weekly supervision sessions between local supervisor and CHW; and 4) quarterly Skype booster training for the supervisors by the Intervention Team. The CHWs and CHW-Ss will complete a separate safety and paperwork training with a member of the Protocol Team (conference call or SKYPE).

7.4 Intervention Monitoring/Quality Control

MI Implementation Intervention (Quality Assurance Protocols). All materials (e.g., slides, training exercises, supervisory tools) will be packaged for potential dissemination as part of the ATN's formal protocol development process (see data management below).

1. The team will hold a centralized 3-day training for CHWs and local coaches. Most components of the training will be delivered to CHWs and CHW-S together as it is not assumed that supervisors will have MI background; however, the last part of the second day, and the third day of training will be split so that CHWs can have more practice with the protocol, and CHW-Ss can focus on coaching MI. The Intervention Team will be trained using modified materials from the National Institute on Drug Abuse (NIDA) and SAMHSA

initiative – Motivational Interviewing Assessment – Supervisor Tools for Enhancing Proficiency (MIA-STEP). However, instead of expecting the CHW-S to code sessions, the Intervention Team will provide external MITI coding and MI Coach Rating Scale (fidelity measure) for the CHW-S to use as feedback, a method that has shown to be superior to supervision or ratings alone (EMMEE Study[88]).

2. Both CHWs and CHW-Ss will submit separate role-play sessions for MITI coding until beginner competency for each is achieved. The Intervention Team will provide coaching and feedback to the local CHW-S via Skype, and will model coaching and feedback for the CHW with the CHW-S observing.

3. Once beginner competency is met and CHWs are cleared to see participants, the local CHW-S will now take over weekly individual supervision of the CHWs. All sessions will be digitally recorded, and one recording per CHW will be randomly selected for MITI coding. CHW-Ss will complete fidelity checklists for the supervision session so the team can monitor implementation. All digital recordings and completed forms and checklists are to be uploaded into its respective folder in Dropbox within one business day of completion.

4. CHW-Ss will attend quarterly boosters via group Skype with the Intervention Team. Prior to the booster, they will submit a recording of a supervision session for review. Boosters will cover successes and challenges, MITI scores, updated MI Skill Development plans for each CHW, and role-plays of supervision skills. The Intervention Team will be available to join supervision sessions if MITI scores fall below competency without remediation. All boosters will be recorded and qualitatively analyzed as part of the implementation Aim 4.

5. At the end of the project, with support from the Research Team, Intervention Team, CHW-S, CHWs, and site PIs will create sustainability plans to continue provision of MI with fidelity both at regularly scheduled clinic visits and potentially to sustain MET for YLH with alcohol and adherence concerns.

8.0 EVALUATIONS AND MEASURES

All measures are administered at baseline, 16 weeks (post baseline), 28 weeks (post baseline), and 52 weeks (post baseline). Baseline data collections should be completed all within the same day, and no later than 7 days after the beginning of the baseline. All follow-up data collections (16-, 28-, and 52-weeks) can be collected up to 14 days prior to their follow up due date and up to 28 days post their follow up due date. Self-report measures will be collected via three mechanisms: The Timeline Follow Back is completed with a RA on a laptop computer, self-reported measures occur using a web-based ACASI (audio computer-administered self-interviewing) survey on a laptop computer at each site, and neurocognitive assessments are administered on a laptop computer by an RA, as well as paper-and-pen interviewing format with a RA. The following neurocognitive assessments will only be completed at baseline and week 28 (7 months) data collection: NIH Tool Box assessments (Flanker task, List Sorting Test, and the Pattern Comparison Test), the Hopkins Verbal Learning Test-Revised (HVLT-R), and NIDA's PhenX Tool Kit (Delayed Reward Discounting Task, Balloon Analog Risk Task). Data collection for biological measures for HIV-VL and alcohol use will be conducted through medical record extraction and/or blood draw (HIV-VL), and finger/toe nail samples (alcohol use; NailStat).

The total assessment time is estimated to be 140 minutes. Measures can be found in the Appendices.

NOTE: The next section is divided into pre-entry evaluations (e.g., screening) and post-entry. Evaluations include measures of participant outcomes, program process and cost, and intervention sustainability.

8.1 Pre-Entry Evaluations and Measures

8.1.1 Screening and Study Entry

The ATN 129 Eligibility and Enrollment Form is used to confirm eligibility. This form contains key eligibility criteria including demographics and viral load. The ATN 129 Randomization Survey is completed at baseline and entered directly into Qualtrics prior to randomization.

8.2 Study Evaluations and Measures (Post-Enrollment)

8.2.1 Primary Outcome Measures

Alcohol Use. Multiple methods are used to assess alcohol use including calendar-based interview, biomarker, and self-report questionnaire.

- Timeline Follow Back (TLFB) Interview: Using a calendar, people provide retrospective estimates of their daily drinking over a time period (the last month) from the interview date.
- Biological Marker of Alcohol Use. NailStat procedure is used as a biological marker of alcohol use. For those with acrylic nails, we try to collect toe nails.
- ASSIST (self-report of alcohol and other substance use). Participants complete the Alcohol, Smoking and Substance Involvement Screening Test (ASSIST) to report lifetime use, three month use and consequences of use for each substance.

HIV Viral Load (HIV-VL). HIV-VL is extracted from medical records or collected if a recent HIV-VL test is not available. For Screening of eligible participants, HIV-VL can be from within 4 weeks from date screening was completed. For baseline data collection, HIV-VL can be +/- 4 weeks from baseline data collection date. For all the follow up data collections (16-, 28-, and 52-weeks, HIV-VL can be +/- 4 weeks from the follow up data collection due date. The *ATN 129 Virology Results Form* should be completed and data entered into Qualtrics.

8.2.2 Secondary Outcome Measures

Medication Adherence. The Visual Analogue Scale (VAS) for Medication Adherence asks participants to consider a specific time period (e.g., previous month) and to estimate along a continuum the percentage of medication doses. The scale is anchored by 0%, 50%, and 100%, with 0% indicating that no medication was taken during the specified time period, 50% half the medication was taken, and 100% indicating that all medication has been taken during the time period.

Sexual Behavior is measured as a General Assessment of Risky Sexual Acts, or prevalence rate of no condom use during sexual intercourse and the number of times of intercourse without a condom obtained by ACASI interview of sexual behavior in the previous 3 months (based on Chen, Murphy, Naar-King, Parsons, and ATN, 2010).

Substance Use. The ASSIST, as described above under alcohol use, is used to measure substance use (other than alcohol).

8.2.3 Social Ecological Factors

Disclosure. Participants respond yes or no as to whether they have disclosed to immediate family members, extended family close friends, and acquaintances.

Life Events. Recent stressful life events will be measured with an adaptation of the Life Events Survey (LES) modified to include only those events that were considered moderate to severely stressful.

Psychological Health. Participant psychological health is measured using the Brief Symptom Inventory (BSI-18). This inventory has been used extensively in medical, psychiatric, and non-patient populations. Participant psychological health related to externalizing behavior is measured using the Behavioral Complexity Scale (BCS).

Social Support. The Patient-Reported Outcomes Measurement Information System (PROMIS) is used to measure social support. PROMIS includes 5 "short forms" of 4 items each assessing emotional support, informational support, instrumental support, social isolation and companionship.

Social Norms for Alcohol. This measure includes items from 2 previous measures. First, it includes specific questions about perception of alcohol use norms (frequency of use in peers, quantity of use in peers, etc.). These include i*njunctive (attitudinal) norms*, originally developed by Larimer et al and d*escriptive (behavioral) norms* originally developed by Baer et al. Second, participants are asked about the drinking of current sexual partners using the Daily Drinking Questionnaire**.**

Stigma. This is measured using the shortened version of Berger's Stigma Scale (10 items).

8.2.4 Neurocognitive Measures

Objective Measures. Subtests from the NIH's "Toolbox" are used to measure attention and executive functioning (Flanker task), working memory (List Sorting Test) and processing speed (Pattern Comparison Test). In addition, participants complete the Hopkins Verbal Learning Test-Revised (HVLT-R) to assess learning of a word list over four trials and retention of the list over a delay. The HVLT-R has a CRF to complete, and then all data on CRF is to be entered into Qualtrics. These four assessments are given at baseline and 28 week (7 month) data collections only.

PhenX Toolkit. Tasks are drawn from NIDA's PhenX Toolkit to measure risk taking and delay and reward preferences. Degree of preference for immediate over delayed rewards is assessed using the Delayed Reward Discounting Task, a questionnaire that presents the subject with a series of scenarios involving balance of delay and reward amount. Risky decision making is assessed using the Balloon Analog Risk Task, a computerized task that involves earning money by inflating balloons to a desired level by entering "pumps" while avoiding bursting the balloon. These two assessments are given at baseline and 28 week (7 month) data collections only.

Youth Report of Cognitive Functioning. Concerns about cognitive functioning over the previous week will be assessed using the NEURO-QOL, a set of NINDS-sponsored "common currency" measures of health-related quality of life. Eight items assess general cognitive concerns and 8 items assess concerns about executive functions. The Prospective and Retrospective Memory Questionnaire (PRMQ) is a measure that assesses the frequency of memory complaints in everyday life. PRMQ has a CRF to complete, and then all data on CRF is to be entered into Qualtrics.

8.2.5 Environmental Measures.

Both self-report and geocoding are used to assess participant environmental factors. The Demographic Questionnaire collects basic demographics such age, ethnicity, gender, income, and zip code, as well as income, employment, insurance, educational history, housing, and access to a working automobile and public transportation. Geocoding is also used to collect data on environment. The current ATN geocoding consultants at Columbia University will develop the following variables: alcohol outlets, neighborhood disadvantage, and public transportation. Location of alcohol consumption is collected through the TLFB procedure described above.

8.3 Process and Cost Effectiveness Measures

The study includes a cost effectiveness analysis via two components: 1) cost analysis of the intervention, and 2) an incremental cost effectiveness analysis that compares the value of the home vs. clinical based treatment conditions.

A modification of The Drug Abuse Treatment Cost Analysis Program (DATCAP), combined with study contact and expenditure records, is used to estimate the cost of the each treatment condition. The DATCAP is a standardized data collection instrument that estimates the economic cost of alcohol treatment programs. Administration of the DATCAP will be a collaborative effort involving our study economist and various members of the intervention staff

administrators, therapist and accounting/finance personnel. The DATCAP organizes program resources into the following categories: personnel, buildings and facilities, supplies and materials, and miscellaneous resources including the value of donated or subsidized items. Client case flow data is incorporated to determine the average annual cost per client for each service type. Other useful computations include weekly cost per client, average cost per intervention episode (based on length of stay in the program), and marginal cost per contact.

CHWs maintain the Implementation Log and Contact Logs for all time spent on the patient-related activities including (calls, contacts, and missed appointments). Via chart review, we obtain information about frequency of clinic visits, appointment history, and contact with CHW during those visits throughout the study to determine potential "dose" of MI beyond the 4-session MET intervention. Both CHWs and supervisors maintain training logs to document all time spent on MI implementation activities.

MITI Coding and the MI Coach Rating Scale are completed by the MI Supervisors to measure intervention fidelity of the CHWs' sessions. The MI Coach Rating Scale will be used to code the MI interactions. The raters will complete 12 items about the quality of implementation (i.e., 1 = Poor, 2 = Fair, 3 = Good, 4 = Excellent) after listening to the interaction (full session). Six items address the relational component of MI and six items address the technical component of MI. The categorized average scores will also be used to assess rates of achieving beginner competence (2.5 - 3.5) and solid competence (≥3.5) or below competence (<2.5).

Evidence Based Practice Attitudes Scale (EBPAS). The EBPAS (Aarons, 2004) is completed by both CHWs and CHW Supervisors prior to their MET Training, immediately post MET Training, and at the end of the study.

8.4 Intervention and Study Evaluations

Participant satisfaction with the treatment is evaluated using the Patient Satisfaction Questionnaire (PSQ) and the Client Experience of Motivational Interviewing scale (CEMI) at 16 week (four month) follow-up.

Qualitative Interviews**.** All participating trainers, staff, and key organization leaders complete an exit interview at the end of the intervention period and the end of the trial to assess sustainability. The qualitative interviews will be completed by the WSU research team and IRB will be obtained only from WSU. All interviews are recorded (with participant's permission).

8.5 Overview of Study Evaluations and Measures by Administration

	ACASI	Paper-and-pencil	Interview	Other (Describe)
ATN 129 Eligibility and Enrollment Form		X		
ATN 129 Randomization Survey				X (Qualtrics)
Timeline Follow-back			X	
NailStat				X (Biological)
Alcohol, Smoking and Substance Involvement Screening Test (ASSIST)	X			
HIV Viral Load (HIV-VL)				X (Biological)
Disclosure	X			
Life Events	X			
Medication Adherence	X			
Psychological Health (BSI-18)	X			
Psychological Health (Externalizing; BCS)	X			
Sexual Behavior	X			

Social Support (PROMIS)	X			
Social Norms for Alcohol	X			
Stigma	X			
NIH Toolbox: Flanker Task				X
NIH Toolbox: List Sorting Test				X
NIH Toolbox: Pattern Comparison Test				X
Hopkins Verbal Learning Test-Revised	X			
PhenX Toolkit: Delayed Reward Discounting Task				X
PhenX Toolkit: Balloon Analog Risk Task				X
NEURO-QOL	X			
Prospective and Retrospective Memory Questionnaire	X			
Demographic Questionnaire	X			
Geocoding				X (geographic data; consultants)
Patient Satisfaction Questionnaire	X			
Client Experience of Motivational Interviewing	X			
The Drug Abuse Treatment Cost Analysis Program				X (Cost)
Implementation Log				X (CHW+)
Contact Log				X (CHW+)
MITI Coding & MI Coach Rating Scale				X (MI Supervisors)
Exit Interview			X	

9.0 DATA COLLECTION AND SITE MONITORING

This study follows ATN standards and recommended guidelines for data management that are specified in Chapter 8 of the ATN MOGO.

9.1 Development of Protocol and Case Report Forms

The Protocol Team is responsible for the development of this protocol as well as the Case Report Forms (CRFs) needed to collect the information required to implement this protocol.

9.2 Data Records

Participant-related study information will be identified through the PID and SID on all participant CRFs, digitally recorded audio files and ACASI files. Participant names or other personally-identifying information will not be used on any study documents. All study-related information will be kept in double-locked, limited access areas at each study site. A log that links the names of participants to their PID and SID numbers will also be kept under double locks separate from all other research records, accessible only to the site research staff, ATN site monitors, and representatives from the National Institute of Child Health and Development (NICHD) and NIAAA. Original source documents for individual participants will be maintained at the respective AMTU and will be accessible only to the study staff. Data from original source documents will be transcribed on CRFs as applicable.

Individuals who do not complete the screening process and those who complete the screening process, but do not consent to participate, will have information collected on the *ATN 129 Screening and Eligibility Form* which will be entered into the study database using a method to maintain anonymity:

- SID numbers will not be assigned to these individuals;
- Personally identifying information will not be collected on this form;

- Individuals will not be listed on any log that could link unique identifiers to individual names; and
- No source documentation will be maintained by the site staff.

9.2.1 File Sharing Site

Dropbox
Dropbox will be used as the study's file sharing site. Study SCs, RAs, CHWs, and CHW-Ss will have access to specific folders according to their study responsibilities to download or upload files. These folders are site specific, meaning that each site only has access to their site Dropbox folder and NOT to any other site folders. Furthermore, there will be separate folders for MET Intervention material versus research study material.

Dropbox Security (*From Dropbox https://www.dropbox.com/help/27/en)

Dropbox provides secure shared folders for storing, sharing, and accessing files across sites. In order to allow others to access Dropbox files, the Project Director must deliberately share these folders. Thus, Dr. Phebe Lam and the research team will invite sites to share specific Dropbox folders. These folders will be accessible once the invitation is accepted and Dropbox is installed by the site. Dropbox accounts and folders are password protected.

At Dropbox, a dedicated security team using the best tools and engineering practices available to build and maintain Dropbox, and has implemented multiple levels of security to protect and back up files. There is also a two-step verification, a login authentication feature which one can enable to add another layer of security to the account.

Other Dropbox users can't see our files in Dropbox unless the Project Director deliberately share links to files or share folders. Dropbox employees are prohibited from viewing the content of files stored in the ATN 129 account. Employees may access file metadata (e.g., file names and locations) when they have a legitimate reason, like providing technical support. Like most online services, a small number of Dropbox employees will be able to access user data for the reasons stated in their privacy policy (e.g., when legally required to do so). But that's the rare exception, not the rule. Dropbox has strict policies and technical access controls that prohibit Dropbox employee access except in these rare circumstances. In addition, Dropbox employ a number of physical, technical, and heuristic security measures to protect user information from unauthorized access. Currently, ATN 128 uses Dropbox for file sharing between the MI trainers and the Protocol Co-Chairs.

9.3 Data Collection

9.3.1 Case Report Forms

Study monitoring data, including information about screening and eligibility, virologic results, etc., will be collected on CRFs. All CRFs and tools for this study will be available for download from the study Dropbox folder.

9.3.2 Digitally Recorded Data

<u>Procedure for Uploading/Downloading Files</u>

All MET sessions are to be digitally recorded (audio) by the CHW. Audio recordings are to be uploaded onto Dropbox (specified folder), the study web-storage space, within **one business day** of session completion. Once the session is saved to Dropbox, the CHW will notify the site RA. Once the RA confirms that it is uploaded (opens audio file to confirm sound), the RA will contact the CHW and he/she will delete the session from the digital audio recorder. This reduces the possibility that a session could be lost in the community should a recorder get lost during a home visit or some other time.

<u>Labeling of the Digital Recording</u>

Following completion of each session (i.e. intervention session) all media (digital audio recordings etc.) will be labeled as follows:

1. Last name of CHW-month/day/year-ID#-Session#
2. If it's role-play the word 'role-play' goes in place of ID number

Responsibilities/Roles

The CHW is responsible for uploading the digital recording, the site RA is responsible for reviewing the files for quality, and communicating directly with CHW to ensure all the necessary data has been received and reviewed. The CHW will be responsible for deleting the session from the digital recorder.

Risks/Safeguards

When not in use, all digital recordings and recorders will be securely maintained at the AMTU in a secured location under double-lock when not in use, and with restricted access during work hours and/or when unattended or should be carried with the CHW at all times or stored in a locked/secure location in the CHW's home. Digital audio-recorders should never be left in a car, even if the car is locked, as they are likely to be stolen in the event of car theft or break-in. Digitally recorded data will not be saved on any of the study laptops. Any loss of data must be immediately reported to the protocol team via the ATN QNS system.

9.3.3 Audio Computer Assisted Self-Interview (ACASI)

All data collected using ACASI will be accessed via Qualtrics which uses a Transport Layer Security (TLS) encryption (also known as SSLv3.1) for all Internet transmitted data. The ACASI responses will remain confidential; no personal identifying information will be collected during the computer session. The participant's unique SID number will be used in order to link the interview responses to the participant's CRF data.

Detailed instructions on the operation of the Qualtrics ACASI will be provided in the ATN 129 Qualtrics ACASI Operating Guide, which will be developed by the Protocol Team and provided to each participating site.

9.3.3.1 ACASI Data Security

Overview of Qualtrics Security (from *Qualtrics Security White Paper*, Jan 14, 2014)

Qualtrics' most important concerns are the protection and reliability of customer data. Our servers are protected by high-end firewall systems, and vulnerability scans are performed regularly. Complete penetration tests are performed yearly. All services have quick failover points with redundant hardware, and complete backups are performed nightly. Qualtrics uses Transport Layer Security (TLS) encryption (also known as SSLv3.1) for all Internet transmitted data. Surveys may be protected with passwords. Our services are hosted by trusted third party data centers that are SSAE-16 SOC 1 Type II audited. All data at rest are encrypted, and data on deprecated hard drives are destroyed by U.S. DOD methods and delivered to a third-party data destruction service.

Qualtrics services allow customers to control individual permissions of their accounts and surveys. This means administrators can decide who creates, distributes, and analyzes their surveys. ATN 129 uses password-protection to protect surveys and to control which users have access. Qualtrics data are maintained and analyzed by the statistical team.

9.4 Data Submission

9.4.1 Case Report Forms (CRFs)

Study sites must follow study protocol guidelines for CRF completion and entry. Research staff at the sites will be responsible for ensuring that applicable CRF data are entered into Qualtrics within the timeframe specified on the CRF.

Each site is responsible for entering data on CRFs into the Qualtrics database. Sites will complete *an ATN 129 Screening and Eligibility Form* and *ATN 129 Monitoring Untoward Event Form* and enter it into Qualtrics within 48 hours after participant enrollment in the study (Screening and Eligibility Form) or when site staff is aware of adverse event (ATN 129 Monitoring Untoward Event Form). All other CRFs must be completed and entered into the database (Qualtrics) within 18 days from the date of the study visit, unless otherwise specified.

Here is a list of all CRFs for the current protocol:

- Study Screening Log
- Screening and Eligibility Form
- Locator Form
- Virology Results Form
- Nail Sample Form
- Off Study Form
- Premature Discontinuation From Study Intervention Form
- Monitoring Untoward Event Form
- Visit Report Form

These CRFs must be signed and dated by the clinical site staff member recording the data.

9.4.2 Qualtrics Randomization

Only authorized users with a login name and password will be able to access Qualtrics for the randomization survey. Each site will receive a unique link to the Qualtrics survey so randomization can be completed at each site independently of other sites. The composition of participants assigned to each condition will be tracked only by the study statistical team. Data are encrypted and stored on a secure, firewall-protected network.

9.5 Data Quality Assurance

Investigators receiving federal funding must adhere to the Code of Federal Regulations (CFR) to protect research participants and produce reliable study information. Sites participating in research sponsored by the NICHD need to have an internal quality assurance (QA) plan that will identify problems and correct errors in research study records. Sites are responsible for following the ATN data quality assurance procedures (see Chapter 9 of the ATN MOGO).

9.6 Role of Data Management

Data will be entered directly by site staff (RA) into Qualtrics. Data will reside on a secure server with firewall protection and SSL encryption. Only the Protocol Co-Chairs, Data Management and Statistical Teams will have administrator privileges enabling them to download data. The database, data structure, and data quality will be routinely reviewed by the Data Management and Statistical Team. Data quality will be examined before statistical analysis can be conducted, including examination of missing data, assessment of distributional assumptions, identification of outliers. In addition to data quality, the comparability between the two intervention groups will be carefully examined, including baseline balance and differential attritions at all waves of follow-up. Where indicated, we will use full-information maximum likelihood estimation to impute missing data under Missing at Random (MAR) assumptions. Where the degree of imputation prevents model convergence, we will explore the use of multiple-imputation strategies or modeling approaches which model missingness across time. If necessary, transformations to normality will be applied.

9.7 Study Site Monitoring and Record Availability

Site monitors from the ATN DOC will visit participating study sites to review a selected portion of the individual participant records, including assent/consent forms, CRFs and supporting source documentation to ensure the protection of study participants, compliance with the protocol, and accuracy and completeness of records. Regulatory files, as required, will also be inspected to ensure that regulatory requirements are being followed.

The site investigator will make study documents (e.g., consent forms, case report forms) and pertinent hospital or clinic records readily available for inspection by the local IRB, the site monitors, the NICHD, the Office of Human Research Protection (OHRP), or the sponsor's designee for confirmation of the study data.

10.0 PARTICIPANT MANAGEMENT

10.1 Tracking Participants / Follow-up

Site SCs, with the help of the site RA, will track participant study recruitment and retention for intervention and follow-up visits. CHWs' tracking of their participants will be monitored by the CHW-Ss.

10.2 Study Visit Management

10.2.1 Completing the ACASI

- The participant is reminded of his or her right to discontinue at any time with no penalty and the right to choose to leave any questions unanswered;
- The participant is given headphones and a laptop or desktop computer in an accommodating, private and quiet area;
- The evaluations and interventions are conducted with ACASI, interview with RA (Timeline FollowBack), and neurocognitive assessments that are computerized and paper-pencil format (completed with a RA);
- If the participant requires a break, AMTU staff will remind the participant that they must restart within 30 minutes in order to continue where they left off. AMTU staff will make sure the computer program is exited and re-entered properly so that the participant's confidentiality is maintained; and
- If the ACASI is discontinued for any reason (during the follow-up visits only), the participant can plan to return to finish the survey another time, up to 7 days from the start of the survey. If the ACASI is not completed within 7 days from the start of the survey, they will have to restart the ACASI from the beginning and will have up to 4 weeks past their follow up due date to complete.

10.2.2 Debriefing and Referral Procedures for ACASI Participants

- The participant will be debriefed about possible reactions to answering questions of a sensitive nature, such as short-term feelings of sadness or anxiety. Participants will be instructed to contact study personnel or to consult the list of referrals provided if feelings persist or worsen after several days;
- Referrals for mental health services will be provided to all participants, if warranted; and
- Before completing the ACASI debriefing, the AMTU staff member present during the session will ask the following question:

"*Is there anything else about the interview that you would like to discuss?*"

If the respondent says *"no,"* she or he should be thanked for participation. The respondent will be given contact information for mental health personnel available at the AMTU and informed that she or he can also contact study personnel in the event that issues or concerns arise later. This contact information for mental health personnel should be a form already used within each AMTU site.

If the response indicates the participant is in urgent need of mental health assistance, site staff should follow their individual site procedures for acute mental health referrals. Site staff should contact a supervisor immediately and stay with the study participant until the supervisor, mental health professional or emergency services, if needed, arrives. Otherwise, the interviewer should say, *"If you decide that you would like to speak with a counselor, here is the contact information for a counselor and a list of agencies in the community that provide this service"* and provide the list of referrals.

10.3 Compensation

Participants are compensated $50 per assessment with no incentives for intervention sessions. Transportation costs (for example, bus tickets, parking reimbursements etc.) for study-related visits will be reimbursed or covered by the study for youth randomized for clinic-based sessions as consistent with the sites' standard of care.

10.4 Intervening on "Social Harms"

All sites have specific policies governing the treatment of human participant. These policies specify that medical and psychological assistance will be available in the immediate environment in the event a participant should experience any adverse reactions resulting from study procedures.

While participants will be informed that they may refuse to answer any question at any time, responses or reactions to certain questions may indicate distress on the part of the participants. If at any time during the study, a participant divulges that he or she is at risk for harm, including but not limited to being abused or experiencing violence, if harm is suspected or likely, or if the participant states he or she is suicidal/homicidal, measures will be taken to ensure his or her safety. Reporting will be done as appropriate to the situation and the legal statutes, including reporting to child protection agencies or other appropriate agencies and referrals will be provided to appropriate support, counseling or treatment resources.

10.5 Criteria for Premature Discontinuation

All premature discontinuations are reported to the protocol team via the ATN QNS within 48 hours.

10.5.1 Premature Discontinuation from Intervention

If there are any concerns that a participant may need to be discontinued from the intervention, the CHW and/or the CHW-S must notify the SC immediately and the SC must within 48 hours send a query to the Protocol Team. The Protocol Team will make a decision on whether the participant is discontinued from the study or only discontinued from the intervention. If the participant is to be prematurely discontinued from the study, please see section 10.5.2 and follow the stated procedures. Complete the *ATN 129 Premature Discontinuation From Study Intervention Form* (Appendices) when the decision is made to permanently discontinue the participant from the intervention but will still remain in the study and will have follow-up study visit evaluations performed per the Schedule of Evaluation Appendices.

Participants prematurely discontinued from the intervention will not be replaced.

10.5.2 Premature Study Discontinuation

Participants will be prematurely discontinued from the study if any of the following occurs:

- Participant fails to comply with the study requirements so as to cause harm to himself/herself or seriously interfere with the validity of the study results.
- Investigator determines that further participation would be detrimental to the health or well-being of the participant.
- Participant develops a health problem and needs treatment that would affect the results of this study.
- Participant withdraws consent/assent or parent/legal guardian withdraws permission.
- Participant becomes incarcerated or detained.
- Study is stopped by a government agency such as the National Institutes of Health (NIH).
- Study is stopped for other administrative reasons.

Complete the *ATN 129 Off Study Form* (Appendices) when the decision is made to permanently discontinue the participant from the study and no further study follow-up or data collection will occur. If the participant was also on the study intervention at the time of Premature Study Discontinuation, complete the *ATN 129 Premature Discontinuation From Study Intervention Form* (Appendices) as well.

Participants prematurely discontinued from the study will not be replaced.

11.0 MONITORING M EVENTS

Site staff must first follow their own IRB's procedure/timeline for reporting and managing untoward events. ATN Behavioral and Community Prevention protocols follow the ATN Policy for Guidance for Safety and Impact Reporting (See Appendix 1-B9 of the ATN MOGO).

There are three types of untoward events to be identified: (1) those related to the participant, (2) those related to the study staff, and (3) those related to the neighborhood/community.

First, the study will catalogue any untoward event experienced by the participant. Reporting is required for occurrences including social harms, psychological distress and serious life threatening events such as suicide attempts. These may be immediately apparent to the study staff, such as the participant's emotional upset requiring referral for counseling; or they may be delayed and reported later to study staff, such as physical harm to an individual for having participated in the study. Study staff will notify the protocol team of these untoward events as soon as possible, but no later than 48 hours after awareness of the event using the ATN QNS accessible through the ATN website (www.atnonline.org). Study staff will be briefed during the training on the scope of possible untoward events and instructed to report them. Site staff will also report the untoward event on the *ATN 129 Monitoring Untoward Event Form* for entry into the study database within three working days after awareness of the event.

Second, study staff may encounter untoward events during sessions that personally affect them. Training and guidance will seek to minimize this risk. Nonetheless, an assessment of the cost of conducting this study must include cataloguing these events as well. The protocol team should be notified of these events within 48 hours of occurrence using the ATN QNS so that they may be immediately addressed, evaluated, and guidance modified or expanded to minimize similar risk to other staff. Sites staff will also report the untoward event on the *ATN 129 Monitoring Untoward Event Form* in the same manner explained above.

Third, a critically important area any community-based study intends to evaluate is the impact, including untoward effects, of the project on the community. This will be done informally for ATN 129 with untoward events on the community being reported to the protocol team via the ATN QNS and documented on the *ATN 129 Monitoring Untoward Event Form* and entered into the study database as described above.

11.1 Study Data Safety & Monitoring Board Members

The study has an external Data Safety and Monitoring Board (DSMB).

12.0 STATISTICAL/ANALYTIC CONSIDERATIONS

12.1 Sample Size and Power Estimates

Power analysis. Power analysis consisted of a 2-step approach. First, power was analyzed assuming independence of participant observations (assuming that the nesting of people-within-clinic was irrelevant). Under these assumptions, the proposed design (N=500; 5 sites) has power = .80 to detect a treatment effect on alcohol use assuming that the treatment accounts for at least 1% of unique variance in the outcome. Preliminary studies have suggested that the intervention effect is at least this large for both alcohol use and HIV viral load. Second, we examined the design effect[147]. The nesting of individuals within clinics has the potential to reduce power because substantial variability in outcome across clinics can obscure level II treatment effects.[147,148] The design effect can be used to tailor power analyses calculated under assumptions of independence. In the case of a Level I predictor with a fixed effect which is uncorrelated with other covariates in the model, the design effect is equal to the $1 - \rho$, where ρ is the intra-class correlation or the percentage of variance accounted for by variability between clinics.[147] The between clinic variability in HIV viral load and alcohol consumption did not differ significantly from zero, suggesting that the design effect would result in a negligible reduction in power. Power in moderation analyses is reduced when one variable is continuous. A sample size of 500 is sufficient to detect a moderation effect with an f^2 of .02. [149] designates this as a small effect; however, more recent work has characterized an effect of this size as moderate to large as applied to moderation.[150] In instances where tests of continuous moderators are marginally significant, we will explore dichotomizing moderators, a practice which increases power in tests of moderation.[150]

12.2 Statistical Analysis Plan

12.2.1 The primary hypothesis is that YLH receiving home-based MET will have greater improvements in alcohol use and HIV viral load than YLH receiving clinic-based MET.

Analytic Plan. Initial analyses will examine whether salient demographic factors were associated with treatment condition after randomization or with attrition over the follow up period. Primary research questions associated with the effect of home-based versus clinic-based delivery on alcohol use and HIV viral load will be addressed through multi-level growth mixture modeling (GMM). Following the methodology of the original Health Choices outcome measurement, we will model the number of days of alcohol use reported during the 7-day period involving the highest level of alcohol use in the assessment period. This operationalization captures the intensity of drinking at highest-use periods. Because all individuals will report some drinking at baseline, individuals who report no drinking during a follow up period will be modeled as true-zeros. If the rate of abstinence is high enough to permit model-convergence, we will model the occurrence of abstinence as a separate (dichotomous) outcome. Log-viral will be treated as continuous outcome. Separate GMM's will be calculated for each primary outcome. Each model will be a two-level model in which individuals (Level I) are nested in clinics (Level II). This approach controls for the non-independence of individuals within clinics. Because 5 sites provide extremely limited predictive power at Level II, no site covariates are included in the model. (The models are empty at Level II). Sites will be deliberately sampled to minimize between-site differences that would have a substantive impact on treatment delivery and intervention outcome. In a GMM, a latent growth curve with an intercept and linear slope factor is specified. Latent class analysis is applied to these two growth components (intercept and slope) to identify groups of individuals who share trajectories. For example, "immediate and sustained responders" may have the lowest post-intervention intercept and a flat slope. Meanwhile, "non-responders" might have the highest post-intervention intercept and a flat slope. In contrast, "delayed responders" might have a high post-intervention intercept but a significant negative slope, indicating reductions in missed medication over the follow up period. If modeling results indicate that discrete classes are not present, we will proceed with analyses in which the growth factors (intercept and slope) are predicted directly by intervention condition (and demographic factors found to be associated with condition after randomization or with attrition over follow up). GMM's can subsequently incorporate predictors of class membership. These analyses can be conceptualized as a multi-nominal logistic regression with the latent trajectory-class membership constituting the outcome variable. The

predictor of primary interest will be intervention condition. We will include as covariates any demographic variables that were associated with condition after randomization or with attrition over the follow up period.

12.2.2 Secondary Analyses: Identification of Moderators of Treatment Effect. Secondary analyses will utilize the trajectory classes identified in primary analyses as their outcome. As such, all analyses will involve multinomial regression. We will vary the referent outcome group to evaluate all possible class-comparisons. The purpose of these analyses is to identify moderators of the association between trajectory class membership and treatment condition. Note, if primary analyses indicate the absence of specific trajectory classes, growth factors (intercept and slope) will be directly modeled as outcomes. We will utilize the framework of the social-ecological model to organize our approach to moderation. Three primary groups of moderators will be explored: individual factors, interpersonal factors, and environmental factors. Initially, we will undertake three groups of exploratory analyses, one for each set of factors. For each group, the significance of individual potential moderators and the significance of a corresponding treatment-by-moderator interaction term will be tested one at a time. Where indicated, and where permitted by the limitations of power and model convergence, we will examine higher order interactions involving multiple potential moderators. Ideally, we will explore ways in which macro-level factors (e.g., neighborhood resources) shape the associations between micro-level factors (e.g., neurocognitive functioning) and treatment outcome.

12.2.3 Cost effectiveness. The cost-effectiveness of HIV treatment has been documented in numerous studies both nationally and globally.[151-157] Findings suggest that interventions that target adherence and provide at least moderate effectiveness are both cost-effective and provide significant long-term health benefits for individuals.[158-162] Further, research has demonstrated that individuals with comorbid alcohol/drug abuse difficulties have significantly higher treatment costs and poorer health outcomes,[163] making the potential benefits of improved adherence substantially greater for individuals who have comorbid alcohol-related problems. Mathematical modeling using local data can improves our understanding of the most cost effective approaches that can be used in community settings to improve HIV health outcomes, and the budget impact expected if they are adopted.[164] We will provide such data by performing an economic analysis as part of this study. This is an efficient means of improving diffusion of effective and high-value interventions, as well as for retarding adoption of inefficient new treatment approaches.

12.2.3.1 MET Program Cost Analysis. We will use a modification of The Drug Abuse Treatment Cost Analysis Program (DATCAP),[165] combined with study contact and expenditure records to estimate the cost of the home-based MET vs. clinic-based MET. The DATCAP is a standardized data collection instrument that estimates the economic cost of alcohol treatment programs.[166,167] Administration of the DATCAP is generally a collaborative effort involving an economist and various members of the intervention staff (administrators, therapist and accounting/finance personnel). The DATCAP organizes program resources into the following categories: personnel, buildings and facilities, supplies and materials, and miscellaneous resources including the value of donated or subsidized items. Client case flow data is incorporated to determine the average annual cost per client for each service type. Other useful computations include travel cost per client, cost of missed appointments, average cost per intervention episode (based on length of stay in the program), and marginal cost per contact.[165,168]

12.2.3.2 Cost Data Analyses. We will estimate the marginal costs of delivering home-based MET vs. clinic-based MET. Using data from the modified DATCAP and study contact and expenditure records, key statistics from the cost evaluation will include total annual economic cost for the program, weekly economic cost per client, and total economic cost per intervention episode. To highlight the relative contribution of the various cost components and support need for data for budgeting in the future, we will also perform a descriptive analysis of the cost accounted for by each resource category. The mean aggregate cost of the two interventions will be used as inputs in the cost effectiveness modeling part of the study. The Clinical Data Analysis for the cost effectiveness modeling will be performed by our Cost Analysis Consultant. The baseline values for HIV-VL and all follow up data will be analyzed and used as input into the model. Mean HIV-VL baseline value to place each participant into a model health state (HS) defined by HIV-VL. The model iterates every quarter, thus participants will be assumed to occupy this HS for the subsequent 91 days. Subsequent occupancy of the model HS will be defined based on the last measured HIV-VL value for the model quarter. This approach will assure that the level of risk of an Acquired Immunodeficiency Syndrome (AIDS) event and rate of progression through the HS in the model (as measured by the HIV-VL suppression rate) are

captured in a consistent manner for all participants. The assumption of a decrease of one HS level will be applied to participants who drop out of the study and thus have missing values for all subsequent model quarters. HS-specific progression rates reported in the literature for patients on highly-active antiretroviral therapy will be used to estimate progression for all participants for time after the end of the study. Cost-effectiveness of MET Delivery Type. Cost-effectiveness will be modeled for home-based MET vs. clinic-based MET for the differences in HIV viral load values over the time of the study and predicted through Markov modeling for 2, 5 and 10 years. The modeling will be performed from the perspectives of 1) a third party payer, 2) the medical care system, and 3) society. Our Cost Analysis Consultant has extensive experience in the use of this type of model for economic analysis of interventions for HIV-disease.[169-171] ***Model Structure***. We will use a published mathematical model of HIV-disease combined with clinical findings from the study and the study intervention cost data, supplemented, as needed, with clinical and epidemiological data from published studies and cost data from archival data bases. The model uses a Markov approach to estimate treatment effects and progression rates for patients with chronic HIV-disease based on their HIV viral load.[164] The programming will be performed in Excel. Crystal Ball Monte Carol estimation software will be used to simulate outcomes for a patient cohort under varying assumptions about the distribution of the data for the model parameters. ***Model Parameters***. The most critical data in the model will come from the clinical parameters (adherence, HIV-VL levels achieved) and the study intervention cost data. Incidence of AIDS-defining conditions and other expected medical events are based on analysis of epidemiologic data from large patient cohorts or on reported values in the literature. Quality of life weights are based on literature reports and/or archival data owned by the investigators. Direct intervention costs for home-based MET vs. clinic-based MET will be estimated as described above. Cost of medical care and drug therapy will be based on Medicaid data; other costs will be based on values in the literature or archival data belonging to the investigators. The effects of different costing perspectives will be assessed. Both cost and years of life will be discounted by 3% when cost effectiveness or cost effectiveness is examined. ***Model Outputs***. The model will estimate 2, 5-year and 10-year flow of fund differences to example individuals, Medicaid, ADAP, and other payers under specific assumptions, as well as overall cost effectiveness and cost utility estimates. Differences in expected population survival, quality of life, and costs attributable to MET will also be reported. One-way and multi-way sensitivity analysis will be performed for parameters that appear to “drive” the results, and a Monte Carlo simulation that varies all parameters will be performed.

12.3 Missing, Unused and Spurious Data

Where indicated, we will use full-information maximum likelihood estimation to impute missing data under Missing at Random (MAR) assumptions. Where the degree of imputation prevents model convergence, we will explore the use of multiple-imputation strategies or modeling approaches which model missingness across time. If necessary, transformations to normality will be applied.

13.0 HUMAN PARTICIPANT

This study will be conducted in compliance with the protocol, International Conference on Harmonization (ICH) Good Clinical Practice (GCP) guidelines, and 45 Code of Federal Regulations (CFR) §46.

13.1 Participants’ Confidentiality

All study-specific laboratory specimens, questionnaires, including the ACASI, interviews/assessments, evaluation forms, CRFs, reports, digital recordings, and other research-related records will be identified by a SID number only, to maintain participant confidentiality. All records must be stored in a secured location under double-lock when not in use, and with restricted access during work hours and/or when unattended. The linkage list that contains information on the participant name and their corresponding PID and SID will be kept in a password protected computer file, with access only to Site Research staff (SC and RA). Nail specimens collected for this study that are sent to the NailStat laboratory will not have the participant’s SID number recorded on their labels or associated requisition forms. All computer entry and networking programs will be done with SID numbers only. Clinical information will not be released without written permission of the participant (and parent or legal guardian, when applicable), except as necessary for monitoring by the ATN DOC or NICHD.

13.2 Certificate of Confidentiality

To further protect the privacy of the study participants, the ATN has obtained a Certificate of Confidentiality from the U.S. Department of Health and Human Services (DHHS). With this Certificate in place, the ATN researchers cannot be forced to turn over identifying information about a study participant in any Federal, State, or local criminal, administrative, legislative, or other proceedings. This Certificate does not prevent a study participant from volunteering to turn over their research information nor does it prevent researchers from providing research-related information to others when requested by the study participant.

13.3 Risks and Benefits

13.3.1 Risks

Risks to participants in this research study may include:
Risk Category: Research not involving greater than minimal risk (45 CRF §46.404 and 21 CFR §50.51)

Participation in this study poses no more harms or discomforts to research participants than they may experience in normal daily life or during routine physical or psychological examinations or tests.

The measurements that are involved in this study require venipuncture to collect blood samples. This procedure may cause local discomfort, bleeding, or bruising; rarely small clot or infection can occur at the blood draw site. This measurement should not be condisered greater thatn minimal risk in and of itself given its routine use in genreal health care delivery.

Participation in this study does not involve any physical risk. However, there is some risk of emotional discomfort or distress due to the personal nature of some questions. Participants will be informed that they are free to decline to answer any questions, or withdraw from participation at any time without penalty. Participants will be instructed to contact study personnel or to consult the list of referrals provided if feelings persist or worsen after several days. If the response indicates the participant is in urgent need of mental health assistance, site staff should follow their individual site procedures for acute mental health referrals. Site staff should contact a supervisor immediately and stay with the study participant until the supervisor, mental health professional or emergency services, if needed, arrives.

13.3.2 Benefits

Possible individual benefits of participation may include:

Information from this study may benefit other youth, now or in the future, by understanding methods to motivate HIV-positive young people to start and adhere to their prescribed HIV treatments and to decrease their alcohol use.
Participant benefits include: (1) improvements in HIV medication adherence, alcohol use, and thus, health status, and (2) assisting in identifying a potential intervention to improve HIV medication adherence, alcohol use, and health outcomes for youth and young adults living with HIV.

13.4 Institutional Review Board (IRB) Review and Informed Consent

This protocol, the informed consent documents and any subsequent modifications will be reviewed and approved by the IRB or ethics committee responsible for the oversight of the study. The informed consent will describe the purpose of the study, the procedures to be followed, and the risks and benefits of participation.

Signed informed consent will be obtained from the participant 18 years and older. For participants 16 to 17 years old, their assent must also be obtained if he or she is able to understand the nature, significance, and risks of the study. The

signed original consent/assent form will be kept on file at the site and a copy of the consent/assent form will be given to the participant. Sample informed consent/assent forms are included in Appendix.

13.5 Waiver of the Requirement for Parental Permission for Special Circumstances

This study will request waiver of parental/guardian permission in accordance with the provision at 45 CRF §46.4116 (c) or §46.4116 (d), include sufficient detail to justify the waiver.)

The site IRBs will be requested to grant waiver of parental permission to participate in this research study for youth participants under the age of 18 (16 years to 17.11 years).

Under 45 CFR 46.408 (c), an IRB has the authority to waive parental permission if it determines that "a research protocol is designed for conditions or a participant population for which parental or guardian permission is not a reasonable requirement to protect the participant" and "an appropriate mechanism for protecting the children who will participate as research participant is substituted" and "that the waiver is not inconsistent with Federal, State, or local law."

Rational for requesting waiver of parental permission:

The protocol team would submit that:

- This study is not considered greater than minimal risk. Participants will complete surveys and supply blood specimens for routine HIV monitoring. None of the content of this study is beyond what would be covered during routine medical or psychological visits or procedures related to the problem behaviors being studied. The probability of harm from participating in this study is no greater than that occurring in routine care.

- The AMTUs involved in the study and most other community agencies offering HIV-related services are confidential and do not require parental/legal guardian notification or permission to treat under state regulations.

- Contacting a parent/legal guardian could constitute a breach of confidentiality for these HIV-positive participants and could potentially put some HIV-positive youth at risk for abuse or ousting from the home if parents/guardians are not aware of their HIV status.

- It is expected that there will be participants who have not disclosed their HIV status to parents/guardians nor will the parents/guardian be aware of the participant's risk behaviors. A requirement for parental permission in this type of study could not only affect a person's willingness to participate, but could also potentially impact the ability of researchers to engage in this type of HIV-related research with youth.

- Adequate protection has been substituted by the mechanisms in place to protect the privacy and confidentiality of participants and by the treatment referrals offered if needed.

13.6 Prisoner Participation

The ATN and NICHD have concluded that this protocol does NOT meet Federal requirements governing prisoner participation in human participant research and should NOT be considered by local IRBs for the recruitment of prisoners. Participant enrolled who subsequently become incarcerated or are placed in detention may not continue study participation. Study visits cannot be conducted during the period of incarceration or detention. This must be reported through the QNS to the Protocol Team and also to the site's local IRB if required.

13.7 45 CFR Parts 160 and 164 Standards for Privacy of Individually Identifiable Health Information ("Privacy Rule" Pursuant to the Health Insurance Portability and Accountability Act - HIPAA)

Each site is responsible for adherence to their individual institution's HIPAA policies and procedures.

13.8 Study Discontinuation

This study may be discontinued at any time by the NICHD and NIAAA.

14.0 PUBLICATION OF RESEARCH FINDINGS

Publication of the results of this trial will be governed by the Protocol Co-Chair and Principle Investigator, Dr. Sylvie Naar-King

15.0 REFERENCES

1. Azar MM, Springer SA, Meyer JP, Altice FL. A systematic review of the impact of alcohol use disorders on HIV treatment outcomes, adherence to antiretroviral therapy and health care utilization. *Drug And Alcohol Dependence*. 2010;112(3):178-193.
2. Hahn JA, Samet JH. Alcohol and HIV disease progression: weighing the evidence. *Curr HIV/AIDS Rep*. 2010;7(4):226-233.
3. Baum M, Rafie C, Lai S, Sales S, Page J, Campa A. Alcohol use accelerates HIV disease progression. *AIDS Research And Human Retroviruses*. 2010;26(5):511-518.
4. Haubrich RH, Little SJ, Currier JS, et al. The value of patient-reported adherence to antiretroviral thearapy in predicting virologic and immunologic response. *AIDS*. 1999;13:1099-1107.
5. Michel L, Carrieri MP, Fugon L, et al. Harmful alcohol consumption and patterns of substance use in HIV-infected patients receiving antiretrovirals (ANRS-EN12-VESPA Study): relevance for clinical management and intervention. *AIDS Care*. 2010;22(9):1136-1145.
6. Samet JH, Horton NJ, Meli S, et al. Alcohol consumption and antiretroviral adherence among HIV-infected persons with alcohol problems. *Alcoholism: Clinical and Experimental Research*. 2004;28:572-577.
7. Samet JH, Phillips SJ, Horton NJ, et al. Detecting alcohol problems in HIV-infected patients: use of the CAGE questionnaire. *AIDS Res. Hum. Retroviruses*. 2004;20:151-155.
8. Kitahata MM, Reed SD, Dillingham PW, et al. Pharmacy-based assessment of adherence to HAART predicts virologic and immunologic treatment response and clinical progression to AIDS and death. *International Journal Of STD & AIDS*. 2004;15(12):803-810.
9. Paterson DL, Swindells S, Mohr J, et al. Adherence to protease inhibitor therapy and outcomes in patients with HIV infection. *Annuals of Internal Medicine*. 2000;133(1):21-30.
10. Piacenti FJ. An Update and Review of Antiretroviral Therapy. Pharmacotherapy. 2006;26:1111-1133.
11. Hendershot CS, Stoner SA, Pantalone DW, Simoni JM. Alcohol use and antiretroviral adherence: review and meta-analysis. *J Acquir Immune Defic Syndr*. 2009;52(2):180-202.
12. Hogg RS, Yip B, Kully C, et al. Improved survival among HIV-infected patients after initiation of triple-drug antiretroviral regimens. *Canadian Medical Association Journal*. 1999;160:659-665.
13. Summers J, Zisook S, Atkinson JH, et al. Psychiatric morbidity associated with acquired immune deficiency syndrome-related grief resolution. *Journal of Nervous Mental Disorder*. 1995;183(384-389).
14. Richman DD. Advances in antiretroviral drug resistance: mechanisms, pathogenesis, clinical significance. *Experimental Medicine & Biology*. 1996;394(383-395).
15. Gardner EM, Sharma S, Peng G, et al. Differential adherence to combination antiretroviral therapy is associated with virological failure with resistance. *AIDS*. 2008;22(1):75-82.
16. Sethi AK, Celentano DD, Gange SJ, et al. Association between adherence to antiretroviral therapy and Human Immunodeficiency Virus drug resistance. *Clinical Infectious Diseases*. 2003;37:1112-1118.
17. Cohen MS, Chen YQ, McCauley M, et al. Prevention of HIV-1 Infection with Early Antiretroviral Therapy. *New England Journal of Medicine*. 2011;365(6):493-505.
18. Cohen MS, Gay CL. Treatment to Prevent Transmission of HIV-1. *Clinical Infectious Diseases*. May 15, 2010 2010;50(Supplement 3):S85-S95.
19. Cohen MS, McCauley M, Gamble TR. HIV treatment as prevention and HPTN 052. *Curr Opin HIV AIDS*. 2012;7:99–105.
20. Garnett GP, Becker S, Bertozzi S. Treatment as prevention: translating efficacy trial results to population effectiveness. *Curr Opin HIV AIDS*. 2012;7:157–163.
21. MacDonell K, Naar-King S, Huszti H, Belzer M. Barriers to medication adherence in behaviorally and perinatally infected youth living with HIV. *AIDS and Behavior*. 2013;17(1):86-93.
22. Tanney MR, Naar-King S, Murphy DA, Parsons JT, Janisse H. Multiple risk behaviors among youth living with Human Immunodeficiency Virus in five U.S. cities. *Journal of Adolescent Health*. 2010;46(1):11-16.
23. UNAIDS. 2008 Report on the global AIDS epidemic. 2008. http://www.unaids.org/en/KnowledgeCentre/HIVData/GlobalReport/2008/2008_Global_report.asp.

24. Galvan A, Hare T, Voss H, Glover G, Casey B. Risk-taking and the adolescent brain: who is at risk? *Developmental science*. 2007;10(2):F8-F14.

25. Hendershot CS, Stoner SA, Pantalone DW, Simoni JM. Alcohol use and antiretroviral adherence: review and meta-analysis. *Journal of acquired immune deficiency syndromes (1999)*. 2009;52(2):180.

26. Monti PPM, Colby PSM, O'Leary TA. *Adolescents, alcohol, and substance abuse: Reaching teens through brief interventions*: The Guilford Press; 2004.

27. Naar-King S, Templin T, Wright K, Frey M, Parsons JT, Lam P. Psychosocial factors and medication adherence in HIV-positive youth. *AIDS Patient Care & STDs*. 2006;20(1):44-47.

28. Naar-King S, Parsons JT, Murphy DA, Chen X, Harris DR, Belzer ME. Improving health outcomes for youth living with the human immunodeficiency virus: A multisite randomized trial of a motivational intervention targeting multiple risk behaviors. *Arch Pediatr Adolesc Med*. 2009;163(12):1092-1098.

29. Murphy DA, Chen X, Naar-King S, Parsons JT, for the Adolescent Trials Network. Alcohol and Marijuana Use Outcomes in the Healthy Choices Motivational Interviewing Intervention for HIV-Positive Youth. *AIDS Patient Care and STDs*. 2012;26(2):95-100.

30. Godwin M, Ruhland L, Casson I, et al. Pragmatic controlled clinical trials in primary care: the struggle between external and internal validity. *BMC Medical Research Methodology*. 2003;3(28):1-7.

31. Kelly JA, Spielberg F, McAuliffe TL. Defining, designing, implementing, and evaluating Phase 4 HIV prevention effectiveness trials for vulnerable populations. *J Acquir Immune Defic Syndr*. 2008;47((Suppl 1)):S28-S33.

32. Prochaska JO, Evers KE, Prochaska JM, Van Marter D, Johnson JL. Efficacy and effectiveness trials examples from smoking cessation and bullying prevention. *J Health Psychol*. 2007;12:170-178.

33. Carroll KM, Rounsaville BJ. Bridging the gap: A hybrid model to link efficacy and effectiveness research in substance abuse treatment. *Psychiatric Services*. 2003;54(3):333-339.

34. Carroll KM, Ball SA, Jackson R, et al. Ten take home lessons from the first 10 years of the CTN and 10 recommendations for the future. *The American Journal of Drug and Alcohol Abuse*. 2011;37:275-282.

35. Naar-King S, Parsons JT, Murphy D, Kolmodin K, Harris DR. A Multisite Randomized Trial of a Motivational Intervention Targeting Multiple Risks in Youth Living With HIV: Initial Effects on Motivation, Self-Efficacy, and Depression. *Journal of Adolescent Health*. 2010;46(5):422-428.

36. Snowden L, Masland M, Ma Y, Ciemens E. Strategies to improve minority access to public mental health services in California: Description and preliminary evaluation. *Journal of Community Psychology*. 2006;34(2):225-235.

37. Gopalan G, Goldstein L, Klingenstein K, Sicher C, Blake C, McKay MM. Engaging families into child mental health treatment: Updates and special considerations. *Journal of the Canadian Academy of Child and Adolescent Psychiatry*. 2010;19(3):182.

38. Scribner R, Theall KP, Simonsen N, Robinson W. HIV Risk and the Alcohol Environment. *Alcohol research & health: the journal of the National Institute on Alcohol Abuse and Alcoholism*. 2009;33(3):179-183.

39. National Institute of Health. Alcohol and HIV/AIDS: Intertwining Stories. In: National Institute on Alcohol Abuse and Alcoholism, ed. http://pubs.niaaa.nih.gov/publications/AA80/AA80.htm: U. S. Dept of Health and Human Services; 2010.

40. DHHS. Panel on Antiretroviral Guidelines for Adults and Adolescents. Guidelines for the use of antiretroviral agents in HIV-1 infected adults and adolescents: Department of Health and Human Services; 2012.

41. Curran GM, Bauer M, Mittman B, Pyne JM, Stetler C. Effectiveness-implementation hybrid designs: combining elements of clinical effectiveness and implementation research to enhance public health impact. *Medical Care*. 2012;50(3):217-226.

42. Fong IW, Read S, Wainberg MA, Chia WK, Major C. Alcoholism and rapid progression to AIDS after seroconversion. *Clinical Infectious Disease*. 1994;19:337-338.

43. Wang JY, Liang B, Watson RR. Alcohol consumption alters cytokine release during murine AIDS. *Alcohol*. 1997;14:155-159.

44. Nachega J, Marconi V, van Zyl G, Gardner E, et al. HIV treatment adherence, drug resistance, virologic failure: Evolving concepts. *Infectious Disorders-Drug Targets (Formerly Current Drug Targets-Infectious*. 2011;11(2):167-174.

45. Flexner CW, Cargill VA, Sinclair J, Kresina TF, Cheever L. Alcohol use can result in enhanced drug metabolism in HIV pharmacotherapy. *AIDS Patient Care and STDs*. 2001;15(2):57-58.

46. Slain D, Pakyz A, Isreal DS, Monroe S, Polk RE. Variability in activity of hepatic CYP3A4 in patients infected with HIV. *Pharmacotherapy*. 2000;20:898-907.

47. Moore RD, Keruly JC, Chaisson RE. Differences in HIV disease progression by injecting drug use in HIV-infected persons in care. *J Acquir Immune Defic Syndr*. 2004;35:46-51.

48. Prakash O, Rodriguez VE, Tang ZY, et al. Inhibition of hematopoietic progenitor cell proliferation by ethanol inhuman immunodeficiency virus type 1 tat-expressing transgenic mice. *Alcoholism: Clinical and Experimental Research*. 2001;25(3):450-456.

49. Conen A, Fehr J, Glass TR, et al. Self-reported alcohol consumption and its association with adherence and outcome of antiretroviral therapy in the Swiss HIV Cohort Study. *Antivir Ther*. 2009;14(3):349-357.

50. Conigliaro J, Gordon AJ, McGinnis KA, Rabeneck L, Justice AC. How harmful is hazardous alcohol use and abuse in HIV infection: do health care providers know who is at risk? *J Acquir Immune Defic Syndr*. 2003;33:521-525.

51. Conigliaro J, Madenwald T, Bryant K, Braithwaite S, et al. The Veterans Aging Cohort Study: Observational Studies of Alcohol Use, Abuse, and Outcomes Among Human Immunodeficiency Virus–Infected Veterans. *Alcoholism: Clinical and Experimental Research*. 2004;28(2):313-321.

52. Justice AC, Sullivan L, Fiellin D, for the Veterans Aging Cohort Study Project Team. HIV/AIDS, comorbidity, and alcohol: Can we make a difference? *Alcohol Research & Health*. 2010;33(3):258-266.

53. Rosenbloom MJ, Sullivan EV, Pfefferbaum A. Focus on the brain: HIV infection and alcoholism. *Alcohol Res Health*. 2010;33(3):247-257.

54. Murphy DA, Belzer M, Durako SJ, et al. Longitudinal Antiretroviral Adherence Among Adolescents Infected With Human Immunodeficiency Virus. *Arch Pediatr Adolesc Med*. 2005;159(8):764-770.

55. Cohen J. Breakthrough of the Year: HIV Treatment as Prevention. *Science*. 2011;334(6063):1628.

56. Duerr A. HIV treatment as protection: next steps. *Current Opinion in HIV and AIDS*. 2012;7(2):97.

57. Hammer SM. Antiretroviral treatment as prevention. *New England Journal of Medicine*. 2011.

58. NYC Department of Health. Health Department Now Recommends Antiretroviral Therapy for Those Diagnosed or Living with HIV2011.

59. Bruce D, Kahana S, Harper GW, Fernández MI, the ATN. Alcohol use predicts sexual risk behavior with HIV-negative or partners of unknown status among young HIV-positive men who have sex with men. *AIDS care*. 2013/05/01 2012;25(5):559-565.

60. UNAIDS. Fact Sheet: Adolescents, young people and HIV. In: Joint United Nations Programme on HIV/AIDS, ed. http://www.unaids.org/en/media/unaids/contentassets/documents/factsheet/2012/20120417_FS_adolescentsyoungpeoplehiv_en.pdf, 2010.

61. Miller WR, Rollnick S. The atmosphere of change. In: Miller WR, Rollnick S, eds. *Motivational interviewing: Preparing people to change addictive behavior*. New York, NY: The Guilford Press; 2002.

62. Martinez J, Bell D, Dodds S, et al. Transitioning youths into care: linking identified HIV-infected youth at outreach sites in the community to hospital-based clinics and or community-based health centers. *Journal of Adolescent Health*. 2003;33(2):23-30.

63. Bradford JB. The promise of outreach for engaging and retaining out-of-care persons in HIV medical care. *AIDS Patient Care and STDs*. 2007;21(S1):S-85-S-91.

64. Rotheram-Borus MJ, Swendeman D, Lee S-J, Li L, Amani B, Nartey M. Interventions for families affected by HIV. *Translational behavioral medicine*. 2011;1(2):313-326.

65. Ventuneac A, Carballo-Diéguez A, Leu C-S, et al. Use of a rapid HIV home test to screen sexual partners: an evaluation of its possible use and relative risk. *AIDS and Behavior*. 2009;13(4):731-737.

66. Were W, Mermin J, Bunnell R, Ekwaru JP, Kaharuza F. Home-based model for HIV voluntary counselling and testing. *The Lancet*. 2003;361(9368):1569.

67. Conner KR, Pinquart M, Gamble SA. Meta-analysis of depression and substance use among individuals with alcohol use disorders. *Journal of Substance Abuse Treatment*. 2009;37(2):127-137.

68. Gamble SA, Conner KR, Talbot NL, Yu Q, Tu XM, Connors GJ. Effects of pretreatment and posttreatment depressive symptoms on alcohol consumption following treatment in project MATCH. *Journal of studies on alcohol and drugs*. 2010;71(1):71.

69. Lejoyeux M, Lehert P. Alcohol-use disorders and depression: results from individual patient data meta-analysis of the acamprosate-controlled studies. *Alcohol and Alcoholism*. 2011;46(1):61-67.

70. Fama R, Rosenbloom MJ, Nichols BN, Pfefferbaum A, Sullivan EV. Working and Episodic Memory in HIV Infection, Alcoholism, and Their Comorbidity: Baseline and 1-Year Follow-Up Examinations. *Alcoholism: Clinical and Experimental Research*. 2009;33(10):1815-1824.

71. Schulte T, Müller-Oehring E, Sullivan E, Pfefferbaum A. White matter fiber compromise contributes differentially to attention and emotion processing impairment in alcoholism, HIV-infection, and their comorbidity. *Neuropsychologia*. 2012.

72. Squeglia LM, Pulido C, Wetherill RR, Jacobus J, Brown GG, Tapert SF. Brain response to working memory over three years of adolescence: Influence of initiating heavy drinking. *Journal of studies on alcohol and drugs*. 2012;73(5):749.

73. Hanson KL, Medina KL, Padula CB, Tapert SF, Brown SA. Impact of adolescent alcohol and drug use on neuropsychological functioning in young adulthood: 10-year outcomes. *Journal of child & adolescent substance abuse*. 2011;20(2):135-154.

74. Anand P, Springer SA, Copenhaver MM, Altice FL. Neurocognitive impairment and HIV risk factors: a reciprocal relationship. *AIDS and Behavior*. 2010;14(6):1213-1226.

75. Heaton R, Clifford D, Franklin D, et al. HIV-associated neurocognitive disorders persist in the era of potent antiretroviral therapy CHARTER Study. *Neurology*. 2010;75(23):2087-2096.

76. Fernie G, Cole JC, Goudie AJ, Field M. Risk-taking but not response inhibition or delay discounting predict alcohol consumption in social drinkers. *Drug and Alcohol Dependence*. 2010;112(1):54-61.

77. Bowden-Jones H, McPhillips M, Rogers R, Hutton S, Joyce E. Risk-taking on tests sensitive to ventromedial prefrontal cortex dysfunction predicts early relapse in alcohol dependency: a pilot study. *The Journal of neuropsychiatry and clinical neurosciences*. 2005;17(3):417-420.

78. Iudicello JE, Woods SP, Cattie JE, Doyle K, Grant I, The HIV Neurobehavioral Research Program (HNRP) Group. Risky decision-making in HIV-associated neurocognitive disorders (HAND). *The Clinical Neuropsychologist*. 2012;27(2):256-275.

79. Thames AD, Streiff V, Patel SM, Panos SE, Castellon SA, Hinkin CH. The role of HIV infection, cognition, and depression in risky decision-making. *The Journal of neuropsychiatry and clinical neurosciences*. 2012;24(3):340-348.

80. Kopera M, Wojnar M, Brower K, et al. Cognitive functions in abstinent alcohol-dependent patients. *Alcohol*. 2012;46(7):665-671.

81. Schuckit MA, Smith TL, Kalmijn J. Relationships Among Independent Major Depressions, Alcohol Use, and Other Substance Use and Related Problems Over 30 Years in 397 Families [OPEN ACCESS]. *Journal of studies on alcohol and drugs*. 2013;74(2):271.

82. Glass TA, McAtee MJ. Behavioral science at the crossroads in public health: extending horizons, envisioning the future. *Social Science & Medicine*. 2006;62(7):1650-1671.

83. Sullivan P GD. The promise of comparative effectiveness research. *JAMA*. 2011;305(4):400-401.

84. Naik AD, Petersen LA. The neglected purpose of comparative-effectiveness research. *New England Journal of Medicine*. 2009;360(19):1929-1931.

85. Mukherjee JS, Eustache FE. Community health workers as a cornerstone for integrating HIV and primary healthcare. *AIDS care*. 2007;19(S1):73-82.

86. Nichols P, Ussery-Hall A, Griffin-Blake S, Easton A. The Evolution of the Steps Program, 2003-2010: Transforming the Federal Public Health Practice of Chronic Disease Prevention. *Preventing Chronic Disease*. 2012;9.

87. Lewin SA, Dick J, Pound P, et al. *Lay health workers in primary and community health care*: The Cochrane Library Database 2005.

88. Miller WR, Yahne CE, Moyers TB, Martinez J, Pirritano M. A randomized trial of methods to help clinicians learn motivational interviewing. *Journal of Consulting and Clinical Psychology*. 2004;72:1050-1062.

89. Mitcheson L, Bhavsar K, McCambridge J. Randomized trial of training and supervision in motivational interviewing with adolescent drug treatment practitioners. *Journal of Substance Abuse Treatment*. 2009;37(1):73-78.

90. Moyers T, Manuel J, Wilson P. A randomized trial investigating training in motivational interviewing for behavioral health providers. *Behavioural and Cognitive Psychotherapy*. 2008;36:149-162.

91. Söderlund LL, Madson MB, Rubak S, Nilsen P. A systematic review of motivational interviewing training for general health care practitioners. *Patient Education and Counseling*. 2010.

92. Martino S, Brigham GS, Higgins C, et al. Partnerships and pathways of dissemination: The National Institute on Drug Abuse--Substance Abuse and Mental Health Services Administration Blending Initiative in the Clinical Trials Network. *Journal of Substance Abuse Treatment*. 2010;38(Supplement 1):S31-S43.

93. Peterson K. Biomarkers for alcohol use and abuse-A summary. *Alcohol Research and Health*. 2004;28(1):30.

94. Jones J, Jones M, Plate C, et al. Liquid chromatography-tandem mass spectrometry assay to detect ethyl glucuronide in human fingernail: comparison to hair and gender differences. *American Journal of Analytical Chemistry*. 2012;3(1):83-91.

95. Morini L, Colucci M, Ruberto MG, Groppi A. Determination of ethyl glucuronide in nails by liquid chromatography tandem mass spectrometry as a potential new biomarker for chronic alcohol abuse and binge drinking behavior. *Analytical and bioanalytical chemistry*. 2012;402(5):1865-1870.

96. Moyers TB, Martin T, Manuel JK, Hendrickson SML, Miller WR. Assessing competence in the use of motivational interviewing. *Journal of Substance Abuse Treatment*. 2005;28(1):19-26.

97. Parsons JT, Rosof E, Punzalan JC, DiMaria L. Integration of motivational interviewing and cognitive behavioral therapy to improve HIV medication adherence and reduce substance use among HIV-positive men and women: Results of a pilot project. *AIDS Patient Care and STDs*. 2005;19(1):31-39.

98. Outlaw AY, Green-Jones M, Naar-King S, de la Cruz. Community-based Intervention Research. In: Stanton B, Galbraith JS, Kaljee L, eds. *The uncharted path from clinic-based to community-based research*: Nova Science Pub., Inc.; 2008:13.

99. Naar-King S, Outlaw A, Green-Jones M, Wright K, Parsons JT. Motivational interviewing by peer outreach workers: a pilot randomized clinical trial to retain adolescents and young adults in HIV care. *AIDS Care: Psychological and Socio-medical Aspects of AIDS/HIV*. 2009;21(7):868 - 873.

100. Fortenberry JD, Martinez J, Rudy BJ, Monte D. Linkage to care for HIV-positive adolescents: a multisite study of the adolescent medicine trials units of the adolescent trials network. *Journal of Adolescent Health*. 2012.

101. Miller PM, Anton RF. Biochemical alcohol screening in primary health care. *Addictive Behaviors*. 2004;29:1427-1437.

102. Williams AB, Rivet Amico K, Bova C, Womack JA. A proposal for quality standards for measuring medication adherence in research. *AIDS & Behavior*. 2012;Epub ahead of print.

103. CDC. PRS Efficacy Criteria for Best-Evidence (Tier 1) Medication Adherence (MA Behavioral Interventions. *HIV/AIDS Prevention* http://www.cdc.gov/hiv/dhap/prb/prs/efficacy/ma/criteria/bestEvidence.html. Accessed May 2013.

104. Miller WR, Zweben A, DiClemente C, Rychtarik RG. *Motivational enhancement therapy manual: A clinical research guide for therapists treating individuals with alcohol abuse and dependence*. Rockville, MD: National Institute on Alcohol Abuse and Alcoholism; 1992.

105. Parsons JT, Golub SA, Rosof E, Holder C. Motivational interviewing and cognitive- behavioral intervention to improve HIV medication adherence among hazardous drinkers: A randomized controlled trial. *J Acqui Immune Defic Syndr*. 2007;46(4):443-450.

106. Naar-King S, Wright K, Parsons J, et al. Healthy Choices: Motivational enhancement therapy for health risk behaviors in HIV+ Youth. *AIDS Education and Prevention*. 2006;18(1):1-11.

107. Naar-King S, Lam P, Wang B, Wright K, Parsons JT, Frey MA. Brief Report: Maintenance of effects of motivational enhancement therapy to improve risk behaviors and HIV-related health in a randomized controlled trial of youth living with HIV. *Journal of Pediatric Psychology*. 2008;33(4):441-445.

108. Naar-King S, Earnshaw P, Breckon J. Toward a Universal Maintenance Intervention: Integrating Cognitive-Behavioral Treatment With Motivational Interviewing for Maintenance of Behavior Change. *Journal of Cognitive Psychotherapy*. 2013;27(2):126-137.

109. National Institute on Alcohol Abuse and Alcoholism. Assessing Alcohol Problems: A guide for clinicians and researchers, 2nd edition. In: U.S. Department of Health and Human Services, ed: National Institute of Health; 2003.

110. Collins RL, Kashdan TB, Koutsky JR, Morsheimer ET, Vetter CJ. A self-administered Timeline Followback to measure variations in underage drinkers' alcohol intake and binge drinking. *Addictive Behaviors*. 2008;33(1):196-200.

111. Rueger SY, King AC. Validation of the Brief Biphasic Alcohol Effects Scale (B-BAES). *Alcoholism: Clinical and Experimental Research*. 2013;37(3):470-476.

112. Jones M, Jones J, Lewis D, et al. Correlation of the alcohol biomarker ethyl glucuronide in fingernails and hair to reported alcohol consumed. *Annual Conference for the Research Society on Alcoholism*. Atlanta, GA; 2011.

113. Newcombe D, Humeniuk R, Hallet C, Ali R. Validation of the World Health Organization Alcohol, Smoking and Substance Involvement Screening Test (ASSIST) and pilot brief intervention. *Parkside, Australia: Drug & Alcohol Services Council of Australia*. 2003.

114. Center for Disease Control and Prevention (CDC). Reported CD4+ T-lymphocyte and viral load results for adults and adolescents with HIV infection - 37 states, 2005-2007. In: HIV Surveillance Supplemental Report 2010, ed. Vol 16, 2011.

115. Mellors JW, Munoz A, Giorgi JV, et al. Plasma viral load and CD4+ lymphocytes as prognostic markers of HIV-1 infection. *Ann Intern Med*. 1997;126(946-954).

116. Hammer SM, Saag MS, Schechter M, et al. International AIDS Society-USA panel. Treatment for adult HIV infection: 2006 recommendations of the International AIDS Society-USA panel. *JAMA*. 2006;296:827-843.

117. Bartlett JG. Medical Management of HIV infections Baltimore: Port City Press; 1999:5-7, 72-73.

118. DHHS. Panel on Antiretroviral Guidelines for Adults and Adolescents. Guidelines for the use of antiretroviral agents in HIV-1 infected adults and adolescents. In: DHHS e, ed2009:1-168.

119. Giordano TP, Guzman D, Clark R, Charlebois ED, Bangsberg DR. Measuring adherence to antiretroviral therapy in a diverse population using a visual analogue scale. *HIV Clin Trials*. 2004;5(2):74-79.

120. Maneesriwongul WL, Tulathong S, Fennie KP, Williams AB. Adherence to antiretroviral medication among HIV-positive patients in Thailand. *J Acquir Immune Defic Syndr*. 2006;43 (Suppl 1):S119-S122.

121. Oyugi JH, Byakika-Tusiime J, Charlebois ED, et al. Multiple validated measures of adherence indicate high levels of adherence to generic HIV antiretroviral therapy in a resource-limited setting. *J Acquir Immune Defic Syndr*. 2004;36(5):1100-1102.

122. Carey KB, Maisto SA, Carey MP, Purnine DM. Measuring readiness-to-change substance misuse among psychiatric outpatients I. Reliability and validity of self-report measures. *Journal of studies on alcohol*. 2001;62(1):79.

123. Weinhardt LS, Carey MP, Maisto SA, Carey KB, Cohen MM, Wickramasinghe SM. Reliability of the timeline follow-back sexual behavior interview. *Annals of Behavioral Medicine*. 1998;20(1):25-30.

124. Parsons J, Lelutiu-Weinberger C, Botsko M, Golub S. Predictors of Day-Level Sexual Risk for Young Gay and Bisexual Men. *AIDS and Behavior*. 2013/05/01 2013;17(4):1465-1477.

125. Rongkavilit C, Naar-King S, Wang B, et al. Motivational Interviewing Targeting Risk Behaviors for Youth Living with HIV in Thailand. *AIDS and Behavior*. 2013:1-12.

126. Derogatis LR, Spencer MS. *The Brief Symptom Inventory (BSI): Administration, scoring, and procedures manual - 1*. Baltimore: John Hopkins University School of Medicine, Clinical Psychometrics Research Unit; 1982.

127. Letendre S, Grant I. Assessment, Diagnosis and Treatment of Human Immunodeficiency Virus (HIV)-Associated Neurocognitive Disorders (HAND): A Consensus Report of the Mind Exchange Program. *Clinical Infectious Diseases*. 2013;56(7):1004-1017.

128. Heaton RK, Franklin DR, Ellis RJ, et al. HIV-associated neurocognitive disorders before and during the era of combination antiretroviral therapy: differences in rates, nature, and predictors. *Journal of neurovirology*. 2011;17(1):3-16.

129. Benedict RH, Schretlen D, Groninger L, Brandt J. Hopkins Verbal Learning Test–Revised: normative data and analysis of inter-form and test-retest reliability. *The Clinical Neuropsychologist*. 1998;12(1):43-55.

130. Stanger C, Ryan SR, Fu H, et al. Delay discounting predicts adolescent substance abuse treatment outcome. *Experimental and Clinical Psychopharmacology*. 2012;20(3):205-212.

131. Lejuez C, Read JP, Kahler CW, et al. Evaluation of a behavioral measure of risk taking: The Balloon Analogue Risk Task (BART). *Journal of Experimental Psychology Applied*. 2002;8(2):75-84.

132. Verbruggen F, Logan GD, Stevens MA. STOP-IT: Windows executable software for the stop-signal paradigm. *Behavior Research Methods*. 2008;40(2):479-483.

133. Cella D, Lai J-S, Nowinski C, et al. Neuro-QOL Brief measures of health-related quality of life for clinical research in neurology. *Neurology*. 2012;78(23):1860-1867.

134. Crawford J, Smith G, Maylor E, Della Sala S, Logie R. The Prospective and Retrospective Memory Questionnaire (PRMQ): Normative data and latent structure in a large non-clinical sample. *Memory*. 2003;11(3):261-275.

135. Woods SP, Carey CL, Moran LM, Dawson MS, Letendre SL, Grant I. Frequency and predictors of self-reported prospective memory complaints in individuals infected with HIV. *Archives of Clinical Neuropsychology*. 2007;22(2):187-195.

136. Khuns, Naar-King S, Garofalo R, Woods M. Prospective Memory and Antiretroviral Adherence in Youth Living with HIV. *8th International Conference on HIV Treatment and Prevention Adherence,* 2013.

137. Carey KB, Henson JM, Carey MP, Maisto SA. Perceived norms mediate effects of a brief motivational intervention for sanctioned college drinkers. *Clinical Psychology: Science and Practice*. 2010;17(1):58-71.

138. Larimer ME, Turner AP, Anderson BK, et al. Evaluating a brief alcohol intervention with fraternities. *Journal of studies on alcohol and drugs*. 2001;62(3):370.

139. Baer JS, Stacy A, Larimer M. Biases in the perception of drinking norms among college students. *Journal of studies on alcohol and drugs*. 1991;52(6):580.

140. Collins RL, Parks GA, Marlatt GA. Social determinants of alcohol consumption: The effects of social interaction and model status on the self-administration of alcohol. *Journal of Consulting and Clinical Psychology*. 1985;53:189-200.

141. Lam PK, Naar-King S, Wright K. Social support and disclosure as predictors of mental health in HIV-positive youth. *AIDS Patient Care and STDs*. 2007;21(1):20-29.

142. Berger BE, Ferrans CE, Lashley FR. Measuring stigma in people with HIV: Psychometric assessment of the HIV stigma scale. *Research in nursing & health*. 2001;24(6):518-529.

143. Tanney MR, Naar-King S, MacDonnel K. Depression and stigma in high-risk youth living with HIV: a multi-site study. *Journal of Pediatric Health Care*. 2011.

144. Leserman J, Ironson G, O'Cleirigh C, Fordiani JM, Balbin E. Stressful life events and adherence in HIV. *AIDS Patient Care and STDs*. 2008;22(5):403-411.

145. Ware Jr JE. Effects of acquiescent response set on patient satisfaction ratings. *Medical Care*. 1978;16(4):327-336.

146. Brogan KE, Naar-King S, Yeh Y, Carcone AI, Madson M. Client Evaluation of Motivational Interviewing (CEMI) Scale with Obese African American Adolescent-Caregiver Dyads. *The Obesity Society annual meeting*. Orlando, FL; 2011.

147. Snijders TAB. Power and Sample Size in Multilevel Linear Models. In: Everitt BS, Howell DC, eds. *Encyclopedia of Statistics in Behavioral Science*. Vol 3. Chicester: Wiley; 2005:1570-1573.

148. Ling EN, Cotter D. Statistical power in comparative aquaculture studies. *Aquaculture*. 2003;224(1):159-168.

149. Cohen J. *Statistical power analysis for the behavioral sciences*: Routledge Academic; 1988.

150. Aguinis H, Beaty JC, Boik RJ, Pierce CA. Effect size and power in assessing moderating effects of categorical variables using multiple regression: A 30-year review. *Journal of Applied Psychology*. 2005;90(1):94-106.

151. Beck EJ, Mandalia S, Sangha R, et al. The Cost-Effectiveness of Early Access to HIV Services and Starting cART in the UK 1996–2008. *PloS one*. 2011;6(12):e27830.

152. Granich R, Kahn JG, Bennett R, et al. Expanding ART for Treatment and Prevention of HIV in South Africa: Estimated Cost and Cost-Effectiveness 2011-2050. *PloS one*. 2012;7(2):e30216.

153. Johnston KM, Levy AR, Lima VD, et al. Expanding access to HAART: a cost-effective approach for treating and preventing HIV. *Aids*. 2010;24(12):1929.

154. Long EF, Brandeau ML, Owens DK. The cost-effectiveness and population outcomes of expanded HIV screening and antiretroviral treatment in the United States. *Annals of internal medicine*. 2010;153(12):778.

155. Schackman BR, Freedberg KA, Weinstein MC, et al. Cost-effectiveness implications of the timing of antiretroviral therapy in HIV-infected adults. *Archives of internal medicine*. 2002;162(21):2478.

156. Schackman BR, Goldie SJ, Weinstein MC, Losina E, Zhang H, Freedberg KA. Cost-effectiveness of earlier initiation of antiretroviral therapy for uninsured HIV-infected adults. *American journal of public health*. 2001;91(9):1456.

157. Sorensen SW, Sansom SL, Brooks JT, et al. A Mathematical Model of Comprehensive Test-and-Treat Services and HIV Incidence among Men Who Have Sex with Men in the United States. *PloS one*. 2012;7(2):e29098.

158. Freedberg KA, Hirschhorn LR, Schackman BR, et al. Cost-Effectiveness of an Intervention to Improve Adherence to Antiretroviral Therapy in HIV-Infected Patients. *JAIDS Journal of Acquired Immune Deficiency Syndromes*. 2006;43:S113-S118 110.1097/1001.qai.0000248334.0000252072.0000248325.

159. Mannheimer SB, Morse E, Matts JP, et al. Sustained Benefit From a Long-Term Antiretroviral Adherence Intervention: Results of a Large Randomized Clinical Trial. *JAIDS Journal of Acquired Immune Deficiency Syndromes*. 2006;43:S41-S47 10.1097/1001.qai.0000245887.0000258886.ac.

160. Simoni JM, Pearson CR, Pantalone DW, Marks G, Crepaz N. Efficacy of Interventions in Improving Highly Active Antiretroviral Therapy Adherence and HIV-1 RNA Viral Load: A Meta-Analytic Review of Randomized Controlled Trials. *JAIDS Journal of Acquired Immune Deficiency Syndromes*. 2006;43:S23-S35 10.1097/1001.qai.0000248342.0000205438.0000248352.

161. Smith-Rohrberg D, Mezger J, Walton M, Bruce RD, Altice FL. Impact of Enhanced Services on Virologic Outcomes in a Directly Administered Antiretroviral Therapy Trial for HIV-Infected Drug Users. *JAIDS Journal of Acquired Immune Deficiency Syndromes*. 2006;43:S48-S53 10.1097/1001.qai.0000248338.0000274943.0000248385.

162. Zaric GS, Bayoumi AM, Brandeau ML, Owens DK. The cost-effectiveness of counseling strategies to improve adherence to highly active antiretroviral therapy among men who have sex with men. *Medical Decision Making*. 2008;28(3):359-376.

163. Conover CJ, Weaver M, Ang A, et al. Costs of care for people living with combined HIV/AIDS, chronic mental illness, and substance abuse disorders. *AIDS care*. 2009;21(12):1547-1559.

164. Simpson K, Voit E, Goodman R, Chumney E. Estimating the social and economic benefits of pharmaceutical innovation: modeling clinical trial results in hiv disease. In: Irina Farquhar KS, Alan Sorkin, ed. *Investing in Health: The Social and Economic Benefits of Health Care Innovation (Research in Human Capital and Development, Volume 14)*: Emerald Group Publishing Limited; 2001:175-196.

165. French MT, Dunlap LJ, Zarkin GA, et al. A structured instrument for estimating the economic cost of drug abuse treatment 1: The Drug Abuse Treatment Cost Analysis Program (DATCAP). *Journal of Substance Abuse Treatment*. 1997;14(5):445-455.

166. French MT, Salomé HJ, M C. Using the DATCAP and ASI to estimate the costs and benefits of residential addiction treatment in the State of Washington. *Social Science & Medicine*. 2002;55(12):2267-2282.

167. French MT, Salomé HJ, Sindelar JL, A M. Benefit- Cost analysis of addiction treatment: methodological guidelines and empirical application using the DATCAP and ASI. *Health Services Research*. 2002;37(2):433-455.

168. Salome HJ, French MT. Using cost and financing instruments for economic evaluation of substance abuse treatment services. In: M G, ed. *Recent developments in alcoholism, Vol. 15: Services research in the era of managed care*. New York: Kluwer Academic/Plenum Publishers; 2001.

169. Simpson KN. Economic Modeling of HIV Treatments. *Current Opinion in HIV and AIDS*. 2010;5(3):242-248.

170. Simpson KN, Baran R, Kirbach SE. Economics of Switching to Second-Line Antiretroviral Therapy with Lopinavir/ritonavir (LPV/r) in Africa: Estimates Based on DART Trial Results and Costs for Uganda and Kenya. *Value Health*. 2011;14(8):1048-1054.

171. Simpson KN, Roberts G, Hicks CB, Finnern HW. Cost-effectiveness of Tipranavir in Treatment Experienced HIV Patients in the US. *HIV Clinical Trials*. 2008;9(4):225-237.

APPENDICES

APPENDIX I: SCHEDULE OF EVALUATIONS

APPENDIX II: ATN 129 PARTICIPATING SITES & PRINCIPAL INVESTIGATORS

APPENDIX III: MEASURES

APPENDIX IV: CASE REPORT FORMS ("CRFS")

APPENDIX V: IRB FORMS

APPENDIX I: SCHEDULE OF EVALUATIONS

	Pre-Entry	Baseline Visit[1]	Intervention Sessions 1-4	4 Month (16 Weeks) Follow-Up Visit (Post-Baseline)	7 Month (28 Weeks) Follow-Up Visit (Post-Baseline)	13 Month (52 Weeks) Follow-Up Visit (Post-Baseline)
**Conduct Screening & Eligibility Form**	X					
**Conduct Screening & Eligibility Verification**	X					
**Signed and Dated Consent/Assent**	X					
**Collect/Update Locator Information**	X	X	X	X	X	X
**Randomization[2] , Assign PID/SID**		X				
**Inform Participant of Treatment Condition[3]**		X				
**Data Collection: ACASI & other assessments**		X		X	X	X
**Nail sample (NailStat)**		X		X	X	X
**Real-time Plasma HIV-1 RNA (HIV viral load)[4]**	X	X		X	X	X
**Schedule Baseline Visit**	X					
**Schedule Follow-Up Visit**		X		X	X	X
**Compensation[5]**		X		X	X	X

[1]Screening and Baseline should occur as close together as possible. Must be <30 days.

[2]Randomization must be conducted immediately after Baseline through Qualtrics; once PID/SID is assigned then participant is considered ENROLLED.

[3]After completion of all baseline assessments and laboratory testing. Youths can meet the CHW (if present) and complete their first MET session after baseline data collection if they are willing and not tired.

[4]Real-time plasma HIV RNA: Required at pre-entry, baseline and each follow-up visit. Participants who have had a HIV viral load drawn within 4 weeks of the pre-entry screening, within 4 weeks of the baseline data collection visit, and within 4 weeks of all the follow-up assessments (16-, 28, and 52-week data collection) will not need to repeat this measure.

[5]Participants are not compensated for attending the Intervention Sessions, except for transportation cost for those in the clinic-based group. Two MET sessions will be completed within weeks one through four, then one session from week five through eight, and one session from week nine through 12.

APPENDIX II: ATN 129 PARTICIPATING SITES AND PRINCIPAL INVESTIGATORS

Children's Hospital of Los Angeles<br>Los Angeles, CA<br>Site 02	Marvin Belzer, MD<br>Children's Hospital of Los Angeles<br>Division of Adolescent Medicine<br>5000 Sunset Blvd, 4th Floor<br>Los Angeles, CA 90027	E-mail:<br>Phone:<br>Fax:	mbelzer@chla.usc.edu<br>323-671-4758<br>323-913-3614
Children's Hospital of Philadelphia<br>Philadelphia, PA<br>Site 04	Steven Douglas, MD<br>CHOP Research Institute<br>CHOP Perelman School of Medicine<br>34th and Civic Center Boulevard<br>Philadelphia, PA 19104	E-mail:<br>Phone:<br>Fax:	douglas@email.chop.edu<br>215-590-3561<br>215-590-3044
Stroger Hospital of Cook County<br>Chicago, IL.<br>Site 05	Jaime Martinez, MD<br>Stroger Hospital of Cook County<br>The Core Center<br>1900 W. Polk Street<br>Administration Building, Room 1110<br>Chicago, IL 60612	E-mail<br>Phone:<br>Fax:	jmartinez@cookcountyhhs.org<br>312-864-3573<br>312-864-9721
	Lisa Henry-Reid, MD<br>Stroger Hospital of Cook County<br>The Core Center<br>1900 W. Polk Street<br>Administration Building, Room 1112<br>Chicago, IL 60612	E-mail:<br>Phone:<br>Fax:	lhenryreid@aol.com<br>312-864-3582<br>312-864-9721
St. Jude Children's Research Hospital<br>Memphis, TN.<br>Site 16	Aditya H. Gaur, MD<br>St. Jude Children's Research Hospital<br>Department of Infectious Diseases<br>262 Danny Thomas Place, Mail Stop 600<br>Memphis, TN 38105	E-mail:<br>Phone:<br>Fax	aditya.gaur@stjude.org<br>901-595-5067<br>901-595-5068
Children's Hospital of Michigan<br>Detroit, MI.<br>Site 19	Elizabeth Secord, MD<br>Children's Hospital of Michigan<br>Division of Allergy, Immunology & Rheumatology<br>3901 Beaubien<br>Detroit, MI 48201	E-mail:<br>Phone:<br>Fax:	esecord@med.wayne.edu<br>313-745-4450<br>313-993-8699

APPENDIX III: MEASURES

1. Study evaluations and measures:

    Primary Outcomes
    Secondary Outcomes
    Social Ecological Factors

2. Process and cost effectiveness measures

3. Intervention and study evaluations

1. Study Evaluations and Measures

Primary Outcome Measures
1) Alcohol Use:
Timeline Follow Back (TLFB) Interview
NailStat procedure
Alcohol, Smoking and Substance Involvement Screening Test (ASSIST)
2) HIV Viral Load

TIMELINE FOLLOW BACK (TLFB) INTERVIEW

What we would like you to do is recall your drinking and drug use for the past month (30 days). We want to know how much alcohol you consumed on each day during the last month. We also want to know how much drugs you used. This is not a difficult task, especially when you use a calendar like this one. (Hand participant reference calendar)

What the calendar does is give you a picture of dates & patterns of your drinking and drug use. Using the calendar also helps you to recall how much you have had to drink and how much drugs you used. The idea is to recall the number of drinks or the amount of drugs you had each day. For the days that you had something to drink or used any drugs, you would tell me exactly what type of drinks you had and also the amount you consumed. For each day you had something to drink or used any drugs, you would also tell me exactly how much money you spent on your drinks or drugs.

Standard holidays such as Christmas are marked on the calendar to help you recall your drinking & drug use around those times. You can also enter in days which may be important for you such as birthdays, vacations, sporting events, parties or any other special days. Do you have any special holidays or dates you want to mark on the calendar to help you better recall your drinking or drug use during the past 30 days? (Direct participant to fill them out on the reference calendar)

People who have a fairly regular drinking or drug use pattern can use the pattern to help them to fill out the calendar. For example, some people may drink more on weekends than during the week. Other people may have a day every week, such as payday or a day when they get together with friends when they are more likely to drink or use drugs.

When filling out the calendar, we want you to be as accurate as possible. We understand that it is difficult to remember exactly how much you may have had to drink, so if you can't remember, take your best guess. For example, if you can't remember if you had 19, 20 or 21 drinks on a certain day choose the midpoint, 20. The important point is that there is a difference if you had 5 or 6 drinks on one day and if you had 20 or 21 drinks or that there is a difference between using marijuana one time or twenty times.

Let's begin. As I said before, what we want you to do is use the calendar to record your drinking and drug use over the past 30 days. Let's start with yesterday (insert yesterday's date) and go back.

1. When did you last drink or use drugs in this 30 day period?

   |____|____|-|____|____|-|____|____|____|____|

   Did not use drugs or alcohol in the last 30 days (0)

2. What type of substance or substances did you use on this day? ***(Select all that apply)***
   Alcoholic beverages (beer, wine, spirits, etc.)
   Cannabis (marijuana, pot, grass, hash, etc.)
   Cocaine (coke, crack, etc.)

Amphetamine type stimulants (speed, diet pills, ecstasy, etc.)
Inhalants (nitrous, glue, petrol, paint thinner, etc.)
Sedatives or Sleeping Pills (Valium, Serepax, Rohypnol, etc.)
Hallucinogens (LSD, acid, mushrooms, PCP, Special K, etc.)
Opioids (heroin, morphine, methadone, codeine, etc.)
Other

3. How much (name substance) did you use on this day?
4. Where did you get or buy (name substance) on this day?
5. How much money did you spend on (name substance) on this day? (round to nearest whole US dollar)

   $ |____|____|____|____|

6. When did you last use drugs or alcohol before that?

   |____|____|-|____|____|-|____|____|____|____|

   Did not use drugs or alcohol before that in the last 30 days (0)

7. What was the greatest amount of alcohol you consumed on any given day during this period?

   |____|____| drinks

   181a. Do you recall when this occurred?

   |____|____|-|____|____|-|____|____|____|____|

8. What was the least amount of drinking during this period?

   |____|____| drinks

   182a. When did it occur?

   |____|____|-|____|____|-|____|____|____|____|

9. As I mentioned earlier, some people have patterns to their drinking that can help them recall their use. Do you have any notable patterns to your drinking?

10. What was the greatest amount you used on any given day during this period?

    |____|____|

    184a. Do you recall when this occurred?

    |____|____|-|____|____|-|____|____|____|____|

11. What was the least amount of drugs you used during this period and when did it occur?

12. As I mentioned earlier, some people have patterns to their drug use that can help them recall their use. Do you have any notable patterns to your drug use?

NailStat Procedure

NailStat procedure is used as a biomarker of alcohol use. Those with acrylic nails or who are unable to provide samples for another reason will be considered missing and handled in the analysis.

NailStat™ Collection Instructions

Nails provide a test sample that is simple to collect, very difficult to adulterate and easy to ship and store. Drugs are incorporated into the nail from the blood stream and remain locked in the nail as it grows. Drugs can be identified in nail clippings 1-2 weeks following ingestion and the window of detection may extend from 3 months up to 6 months after the last usage. Environmental exposure is immediately detectable. Fingernails grow approximately 3 millimeters (mm) to 5 mm per month, and toenails grow at a much slower rate of 1 mm per month. Drugs can be detected in toenail clippings up to 1 year after ingestion. The optimum amount of nail clippings needed is 100 mg. If trimming all 10 fingernails, each clipping must be at least 2 mm to 3 mm long.

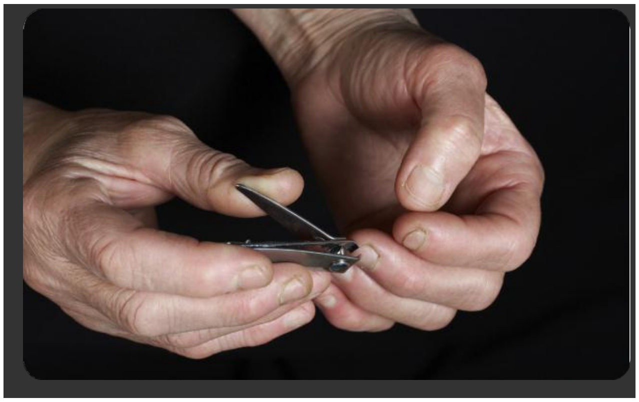

Unlike other matrices, nail collection introduces the opportunity for potential harm to the client. This is an observed collection with the client clipping his or her own nails. USDTL recommends non-cosmetically treated scalp hair collection when fingernails are too short. USDTL accepts toenails or body hair, but cannot provide in-depth, time-line, follow-back history with results. Collectors should avoid toenail collection from a client with peripheral artery disease or diabetes due to reduced circulation and potential infection.

Materials needed for collection:

Provided by USDTL:

- Envelope with security seal and foil inside
- Plastic specimen bag
- Requisition form / chain-of-custody (test subject ID number, date of collection, date and signature of sample collector must be completed at the time of collection)

WSU Research Team will provide to sites (including Pre-Paid Courier Bag/Envelope):

- Metal nail clipper: Each participant will have their own nail clipper, and will NOT share with anyone else. The nail clipper will have a label that has the participant's SID number on it. The site will retain for them throughout the study in a container (e.g., envelope or plastic bag) labeled with the participant's SID.
- Isopropyl alcohol
- Gem scale

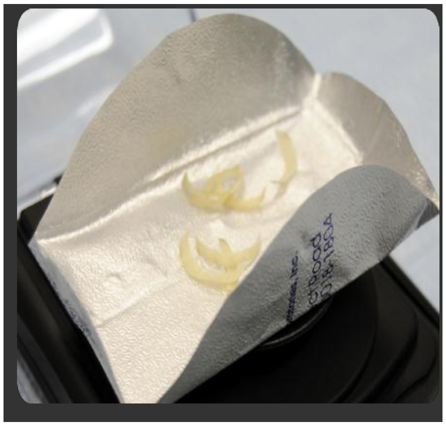

Open the nail/hair collection material in the presence of the client.

1. Confirm that nails do not have an artificial acrylic, gel or silk overlay. Consider another sample matrix, hair or toenails, if an overlay is present. Do not collect toenails if client is diabetic or suffers from peripheral artery disease. It is not necessary to remove nail polish.

2. Have client wash hands with soap and water prior to sample collection.

3. Soak clippers in either a 70% or a 91% isopropyl alcohol solution for 10 minutes prior to each use. **Do not use an ethyl alcohol products.**

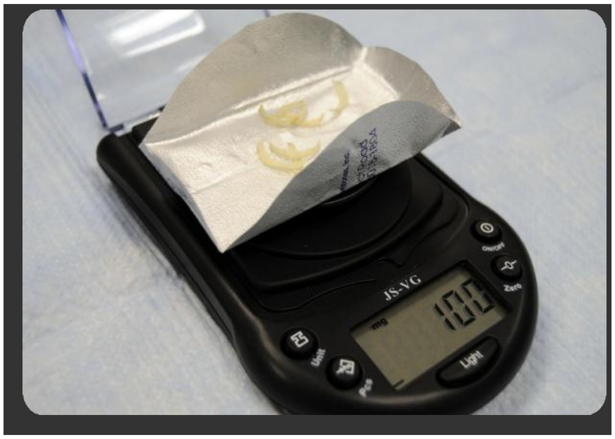

4. Observe client clipping nails over an 8-inch x 11-inch sheet of paper. Clip entire fingernail as close to the nail bed as comfortable, at least 2 millimeters of nail from all 10 fingers. Assay requires 100 milligrams of nail sample. (Photographs show 100 milligrams of nails.) A gem scale may be used for a more accurate sample collection.

5. Fold each side of the foil sample collector up to form a tray-like object. Pour the nails collected on the 8-inch x 11-inch sheet of paper into the foil tray. Fold each side of the foil tray inward to secure the nails. Place foil collector in the specimen collection envelope.

6. Seal the envelope with red security seal and initial seal. Place chain-of-custody number sticker from the requisition form on the envelope. Have the donor (guardian/witness/etc.) read and initial the appropriate area on the envelope, then date, print and sign their name on the requisition form. Date, print and sign the requisition form. This initiates chain-of-custody.

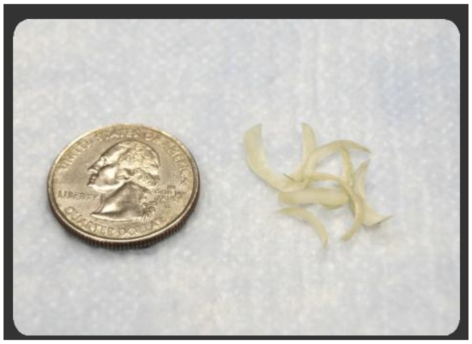

7. In the presence of the donor, place the white copy of the requisition form in the outer pocket of the security bag. Place the envelope in the other pocket of the security bag. Then, seal and initial the bag. The other copies of the requisition form are to be distributed at the discretion of the collecting institution.

8. Be sure that you indicate which NailStat test(s) you require.

9. Mail in Courier bag/envelope to NailStat company. WSU Research Team is covering cost of delivery.

Alcohol, Smoking and Substance Involvement Screening Test (ASSIST)

Now I am going to ask you some questions about your experience of using alcohol, tobacco products and other drugs across your lifetime and in the past three months. These substances can be smoked, swallowed, snorted, inhaled, injected or taken in the form of pills.

Some of the substances listed may be prescribed by a doctor (like amphetamines, sedatives, pain medications). For this section, we will not record medications that are used as prescribed by your doctor. However, if you have taken such medications for reasons other than prescription, or taken them more frequently or at higher doses than prescribed, please let me know. While we are also interested in knowing about your use of various illicit drugs, please be assured that information on such use will be treated as strictly confidential.

Section 1

**In your life, which of the following substances have you ever used? (NON-MEDICAL USE ONLY)**	**No**	**Yes**
1. Tobacco products (cigarettes, chewing tobacco, cigars, etc.)	☐	☐
2. Alcoholic beverages (beer, wine, spirits, etc.)	☐	☐
3. Cannabis (marijuana, pot, grass, hash, etc.)	☐	☐
4. Cocaine (coke, crack, etc.)	☐	☐
5. Amphetamine type stimulants (speed, diet pills, ecstasy, etc.)	☐	☐
6. Inhalants (nitrous, glue, petrol, paint thinner, etc.)	☐	☐
7. Sedatives or Sleeping Pills (Valium, Serepax, Rohypnol, etc.)	☐	☐
8. Hallucinogens (LSD, acid, mushrooms, PCP, Special K, etc.)	☐	☐
9. Opioids (heroin, morphine, methadone, codeine, etc.)	☐	☐
10. Other	☐	☐
10A. Please specify any other substances that you have ever used. ____________________		

Section 2

**[HAND ASSIST REFERENCE CARD TO PARTICIPANT]** **In the past three months, how often have you used....**	**0. Never**	**1. Once or Twice**	**2. Monthly**	**3. Weekly**	**4. Daily or Almost**
11. Tobacco products (cigarettes, chewing tobacco, cigars, etc.)?	☐	☐	☐	☐	☐
12. Alcoholic beverages (beer, wine, spirits, etc.)?	☐	☐	☐	☐	☐
13. Cannabis (marijuana, pot, grass, hash, etc.)?	☐	☐	☐	☐	☐
14. Cocaine (coke, crack, etc.)?	☐	☐	☐	☐	☐
15. Amphetamine type stimulants (speed, diet pills, ecstasy, etc.)?	☐	☐	☐	☐	☐
16. Inhalants (nitrous, glue, petrol, paint thinner, etc.)?	☐	☐	☐	☐	☐
17. Sedatives or Sleeping Pills (Valium, Serepax, Rohypnol, etc.)?	☐	☐	☐	☐	☐
18. Hallucinogens (LSD, acid, mushrooms, PCP, Special K, etc.)?	☐	☐	☐	☐	☐

19. Opioids (heroin, morphine, methadone, codeine, etc.)?	☐	☐	☐	☐	☐
20. [Other (Q10) from Section 1]?	☐	☐	☐	☐	☐

Section 3

**During the past three months, how often have you had a strong desire or urge to use...**	**0. Never**	**1. Once or Twice**	**2. Monthly**	**3. Weekly**	**4. Daily or Almost**
21. Tobacco products (cigarettes, chewing tobacco, cigars, etc.)?	☐	☐	☐	☐	☐
22. Alcoholic beverages (beer, wine, spirits, etc.)?	☐	☐	☐	☐	☐
23. Cannabis (marijuana, pot, grass, hash, etc.)?	☐	☐	☐	☐	☐
24. Cocaine (coke, crack, etc.)?	☐	☐	☐	☐	☐
25. Amphetamine type stimulants (speed, diet pills, ecstasy, etc.)?	☐	☐	☐	☐	☐
26. Inhalants (nitrous, glue, petrol, paint thinner, etc.)?	☐	☐	☐	☐	☐
27. Sedatives or Sleeping Pills (Valium, Serepax, Rohypnol, etc.)?	☐	☐	☐	☐	☐
28. Hallucinogens (LSD, acid, mushrooms, PCP, Special K, etc.)?	☐	☐	☐	☐	☐
29. Opioids (heroin, morphine, methadone, codeine, etc.)?	☐	☐	☐	☐	☐
30. [Other (Q10) from Section 1]?	☐	☐	☐	☐	☐

Section 4

**During the <u>past three months</u>, how often has your use of *(FIRST DRUG, SECOND DRUG, ETC*) led to health, social, legal or financial problems?**	**0. Never**	**1. Once or Twice**	**2. Monthly**	**3. Weekly**	**4. Daily or Almost Daily**
31. Tobacco products (cigarettes, chewing tobacco, cigars, etc.)	☐	☐	☐	☐	☐
32. Alcoholic beverages (beer, wine, spirits, etc.)	☐	☐	☐	☐	☐
33. Cannabis (marijuana, pot, grass, hash, etc.)	☐	☐	☐	☐	☐
34. Cocaine (coke, crack, etc.)	☐	☐	☐	☐	☐
35. Amphetamine type stimulants (speed, diet pills, ecstasy, etc.)	☐	☐	☐	☐	☐
36. Inhalants (nitrous, glue, petrol, paint thinner, etc.)	☐	☐	☐	☐	☐
37. Sedatives or Sleeping Pills (Valium, Serepax, Rohypnol, etc.)	☐	☐	☐	☐	☐
38. Hallucinogens (LSD, acid, mushrooms, PCP, Special K, etc.)	☐	☐	☐	☐	☐
39. Opioids (heroin, morphine, methadone, codeine, etc.)	☐	☐	☐	☐	☐
40. [Other (Q10) from Section 1]?	☐	☐	☐	☐	☐

Section 5

**During the <u>past three months</u>, how often have you failed to do what was normally expected of you because of your use of...**	**0. Never**	**1. Once or Twice**	**2. Monthly**	**3. Weekly**	**4. Daily or Almost Daily**
41. Tobacco products (cigarettes, chewing tobacco, cigars, etc.)?	☐	☐	☐	☐	☐
42. Alcoholic beverages (beer, wine, spirits, etc.)?	☐	☐	☐	☐	☐
43. Cannabis (marijuana, pot, grass, hash, etc.)?	☐	☐	☐	☐	☐
44. Cocaine (coke, crack, etc.)?	☐	☐	☐	☐	☐
45. Amphetamine type stimulants (speed, diet pills, ecstasy, etc.)?	☐	☐	☐	☐	☐
46. Inhalants (nitrous, glue, petrol, paint thinner, etc.)?	☐	☐	☐	☐	☐
47. Sedatives or Sleeping Pills (Valium, Serepax, Rohypnol, etc.)?	☐	☐	☐	☐	☐
48. Hallucinogens (LSD, acid, mushrooms, PCP, Special K, etc.)?	☐	☐	☐	☐	☐
49. Opioids (heroin, morphine, methadone, codeine, etc.)?	☐	☐	☐	☐	☐
50. [Other (Q10) from Section 1]?	☐	☐	☐	☐	☐

Section 6

**Has a friend or relative or anyone else <u>ever</u> expressed concern about your use of...**	**0. Never**	**1. Yes, but not in the past 3 months**	**2. Yes, in the past 3 months**
51. Tobacco products (cigarettes, chewing tobacco, cigars, etc.)?	☐	☐	☐
52. Alcoholic beverages (beer, wine, spirits, etc.)?	☐	☐	☐
53. Cannabis (marijuana, pot, grass, hash, etc.)?	☐	☐	☐
54. Cocaine (coke, crack, etc.)?	☐	☐	☐
55. Amphetamine type stimulants (speed, diet pills, ecstasy, etc.)?	☐	☐	☐
56. Inhalants (nitrous, glue, petrol, paint thinner, etc.)?	☐	☐	☐
57. Sedatives or Sleeping Pills (Valium, Serepax, Rohypnol, etc.)?	☐	☐	☐
58. Hallucinogens (LSD, acid, mushrooms, PCP, Special K, etc.)?	☐	☐	☐
59. Opioids (heroin, morphine, methadone, codeine, etc.)?	☐	☐	☐
60. [Other (Q10) from Section 1]?	☐	☐	☐

Section 7

Have you ever tried and failed to control, cut down or stop using...	0. Never	1. Yes, but not in the past 3 months	2. Yes, in the past 3 months
61. Tobacco products (cigarettes, chewing tobacco, cigars, etc.)?	☐	☐	☐
62. Alcoholic beverages (beer, wine, spirits, etc.)?	☐	☐	☐
63. Cannabis (marijuana, pot, grass, hash, etc.)?	☐	☐	☐
64. Cocaine (coke, crack, etc.)?	☐	☐	☐
65. Amphetamine type stimulants (speed, diet pills, ecstasy, etc.)?	☐	☐	☐
66. Inhalants (nitrous, glue, petrol, paint thinner, etc.)?	☐	☐	☐
67. Sedatives or Sleeping Pills (Valium, Serepax, Rohypnol, etc.)?	☐	☐	☐
68. Hallucinogens (LSD, acid, mushrooms, PCP, Special K, etc.)?	☐	☐	☐
69. Opioids (heroin, morphine, methadone, codeine, etc.)?	☐	☐	☐
70. [Other (Q10) from Section 1]?	☐	☐	☐

Section 8

	Never	Yes, but not in the past 3 months	Yes, in the past 3 months
71. Have you ever used any drug by injection? *(NON-MEDICAL USE ONLY)*	☐	☐	☐

Secondary Outcome Measures (Adherence, Sexual Risk, and Other Substance Use)

1. *Visual Analogue Scale (VAS) for Medication Adherence*
2. General Assessment of Risky Sexual Acts (*Sex Risk Behavioral Scale*)
3. *ASSIST* **(as listed in previous section)**

<u>Visual Analogue Scale (VAS) of Medication Adherence</u>

Now I'm going to ask some questions about your HIV medications.

Most people with HIV have many pills or other medications to take at different times during the day. Many people find it hard to always remember to take their pills or medicines. For example:

- Some people get busy and forget to carry their pills with them.
- Some people find it hard to take their pills according to all the instructions, such as "with food" or "on an empty stomach," "every 8 hours," or "with plenty of fluids."
- Some people decide to skip taking pills to avoid adverse effects or to just not take pills that day.

We need to understand what people with HIV are really doing with their pills or medicines. Please tell us what you are actually doing. Don't worry about telling us you don't take all your pills or medicines. We need to know what is really happening, not what you think we "want to hear."

Which antiretroviral medications have you been prescribed to take within the last 30 days?

INTERVIEWER: LIST CODES FOR ALL ANTIRETROVIRALS THAT SUBJECT WAS PRESCRIBED TO TAKE IN LAST 30 DAYS. IDENTIFY UP TO 4 DRUGS

Now, I am going to ask you some questions about these drugs. Please put an "X" on the line below at the point showing your best guess about how much (DRUGS A-D) you have taken in the last 3-4 weeks. We would be surprised if this were 100% for most people.

0% means you have taken no (DRUG A)

50% means you have taken half your (DRUG A)

100% means you have taken every single dose of (DRUG A)

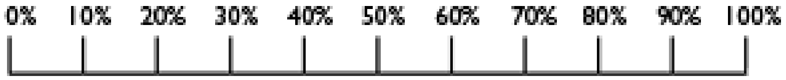

General Assessment of Risky Sexual Acts
Sexual Risk Behavioral Scale

These next questions ask about very specific things about sexual behavior. You may or may not do these things all the time. We know everyone's answers might be different. Remember, your answers will not be shared with anyone.

*****Biological Male Version (includes male to female transgender)*****

Section 1 – Sex with Biological Males (includes male to female transgender) in Last 3 Months (Biological Male participants only. All visits.)

1. In the last 3 months, how many biological males (including male to female transgender) have you had anal sex with? ***(If 0, skip to Section 2)***

2. Thinking about the last three months **[name time frame],** how many times have you had anal sex performed to you by a biological male (his penis in your rectum)? ***(If 0, Go to 3)***

   2A. How many of those times was your partner wearing a condom? ***If (2 - 2A) = 0, Skip to 3)***

   2B. So ***[2 - 2A]*** times, your partner was not wearing a condom. How many of those times were you under the influence of drugs or alcohol?

3. Thinking about the last three months, how many times have you performed anal sex to a biological male (your penis in his rectum)? ***(If 0, Go to Section 2)***

   3A. How many of those times were you wearing a condom? ***(if (3 - 3A) = 0, Skip to Section 2)***

   3B. So ***[3 - 3A]*** times, you were not wearing a condom. How many of those times were you under the influence of drugs or alcohol?

Section 2 – Sex with Females in Last 3 Months (Male participants only. All visits.)

4. In the last 3 months, how many biological women (includes female to male transgender) have you had intercourse with (vaginal or anal sex)? ***(If 0, skip to Section 4)***

5. Thinking about the last three months **[name time frame],** how many times have you had vaginal sex with a biological woman (your penis in her vagina)? ***(If 0, Go to 6)***

   5A. How many of those times were you or your partner wearing a condom? ***(if (5 - 5A) = 0, Skip to 6)***

   5B. So **[5 - 5A]**, times you did not use a condom. How many of those times were you under the influence of drugs or alcohol?

6. Thinking about the last three months **[name time frame],** how many times have you performed anal sex to a biological woman (your penis in her rectum)? ***(If 0, Skip to Section 4)***

6A. How many of those times were you wearing a condom? ***(if (6 – 6A) = 0, Skip to Section 4)***

6B. So ***[6 – 6A]*** times, you were not wearing a condom. How many of those times were you under the influence of drugs or alcohol?

*****Biological Female Version*****

Section 3 - Sex with biological Males in Last 3 Months (Biological Female participants only including female to male transgender. All visits.)

7. In the last 3 months, how many men have you had intercourse with (vaginal, or anal)? ***(If 0, Skip to Section 4)***

8. Thinking about the last three months, how many times have you had vaginal sex performed to you by a biological male (his penis in your vagina)? ***(If 0, Skip to 9)***

8A. How many of those times were you or your partner wearing a condom? ***(if (8 - 8A) = 0 Skip to 9)***

8B. So ***[8 - 8A]*** times, you did not use a condom. How many of those times were you under the influence of drugs or alcohol?

9. Thinking about the last three months, how many times have you had anal sex with a biological male (his penis in your rectum)? ***(If 0, Skip to Section 4)***

9A. How many of those times was your partner wearing a condom? ***(if (9 - 9A) = 0 Skip to Section 4)***

9B. So ***[9 - 9A]*** times, your partner was not wearing a condom. How many of those times were you under the influence of drugs or alcohol?

Section 4: STDs and Pregnancy (MALES AND FEMALES)

10.	***(Baseline Visit)*** Have you ever been diagnosed with gonorrhea (GC)?<br><br>***(Non-Baseline Visit)*** Have you been diagnosed with gonorrhea (GC) since your last interview on **[insert date of last visit]**?	No ***(Go to 11)***<br>Yes

10A. What is the month and year of the most recent episode of gonorrhea (GC)?
|____|____|--|____|____|____|____|

11.	***(Baseline Visit)*** Have you ever been diagnosed with Chlamydia?<br><br>***(Non-Baseline Visit)*** Have you been diagnosed with Chlamydia since your last interview on **[insert date of last visit]**?	No ***(Go to 12)***<br>Yes

11A. What is the month and year of the most recent episode of Chlamydia?
|_____|_____|--|_____|_____|_____|_____|

12.	***(Baseline Visit)*** Have you ever been diagnosed with syphilis?<br><br>***(Non-Baseline Visit)*** Have you been diagnosed with syphilis since your last interview on **[insert date of last visit]**?	No ***(Go to 13)***<br>Yes

12A. What is the month and year of the most recent episode of syphilis?
|_____|_____|--|_____|_____|_____|_____|

13.	***(Baseline Visit)*** Have you ever been diagnosed with Trichomonas (trich)?<br><br>***(Non-Baseline Visit)*** Have you been diagnosed with Trichomonas (trich) since your last interview on **[insert date of last visit]**?	No ***(Go to 14)***<br>Yes

13A. What is the month and year of the most recent episode of Trichomonas (trich)?
|_____|_____|--|_____|_____|_____|_____|

14.	***(Baseline Visit)*** Have you ever been diagnosed with Herpes?<br><br>***(Non-Baseline Visit)*** Have you been diagnosed with Herpes since your last interview on **[insert date of last visit]**?	No ***(Go to 15)***<br>Yes

14A. What is the month and year of the first episode?
|_____|_____|--|_____|_____|_____|_____|

14B. What is the month and year of the most recent episode of Herpes?
|_____|_____|--|_____|_____|_____|_____|

15.	***(Baseline Visit)*** Have you ever been diagnosed with Venereal Warts/ Genital Warts (HPV)?<br><br>***(Non-Baseline Visit)*** Have you been diagnosed with Venereal Warts/ Genital Warts (HPV) since your last interview on **[insert date of last visit]**?	No ***(Females go to 16, Males go to 19)***<br>Yes

15A. What is the month and year of the first episode?
|_____|_____|--|_____|_____|_____|_____|

15B. What is the month and year of the most recent episode of Venereal Warts/ Genital Warts (HPV)?

|_____|_____|--|_____|_____|_____|_____|

FEMALES ONLY

16. ***(Baseline Visit)*** Have you ever been diagnosed with Pelvic Inflammatory Disease (PID)?

(Non-Baseline Visit) Have you been diagnosed with Pelvic Inflammatory Disease (PID) since your last interview on **[insert date of last visit]**?

No ***(Go to 17)***
Yes

16A. What is the month and year of the first episode?
|____|____|--|____|____|____|____|

16B. What is the month and year of the most recent episode of Pelvic Inflammatory Disease (PID)?
|____|____|--|____|____|____|____|

17. ***(Baseline Visit)*** Have you ever had an abnormal pap smear?

(Non-Baseline Visit) Have you had an abnormal pap spear since your last interview on **[insert date of last visit]**?

No ***(Go to 18)***
Yes

17A. What is the month and year of the first time you had an abnormal pap smear?
|____|____|--|____|____|____|____|

17B. What is the month and year of the most recent time you had an abnormal pap smear?
|____|____|--|____|____|____|____|

18. How many times have you been pregnant? ***(If 0, skip to END)***

18A. How many of these pregnancies were planned?

18B. Have you gotten pregnant in the last 3 months?
NO
YES

Biological MALES ONLY

19. How many times have you gotten a partner pregnant***? (If 0, skip to END)***

19A. How many of these pregnancies were planned?

19B. Have you gotten someone pregnant in the last 3 months?
NO
YES

Social Ecological Factors

1. Disclosure (2 item self-report)
2. *Life Events Survey (LES)*
3. *Brief Symptom Inventory (BSI-18)*
4. *Behavioral Complexity Scale (BSC)*
5. *Patient-Reported Outcomes Measurement Information System (PROMIS)*
6. Social Norms for Alcohol.
    - Larimer et al.: Perception of alcohol use norms
    - *Daily Drinking Questionnaire* (drinking of sexual partners)
7. *Stigma Scale*
8. NIH ToolBox (Neurocognitive measures)
    - *Flanker Task*
    - *List Sorting Test*
    - *Pattern Comparison Test*
9. *Hopkins Verbal Learning Test-Revised (HVLT-R)*
10. NIDA's PhenX Toolkit
    - *Delayed Reward Discounting Task,*
    - *Balloon Analog Risk Task*
11. *Quality of Life in Neurological Disorders measurement system (NEURO-QOL)*
12. *Prospective and Retrospective Memory Questionnaire (PRMQ)*
13. Demographic Questionnaire
14. Geocoding

<u>Disclosure (Self-Report)</u>

1. Have you disclosed your HIV status to anyone?
    - Yes ***(Go to Q2)***
    - No

2. Who have you told? (Choose all that apply).
    - Current sex partner who is not a steady girlfriend or boyfriend
    - Past sex partner who is not a steady girlfriend or boyfriend
    - Current steady boyfriend (someone you knew for a while with whom you have an ongoing relationship)
    - Past steady boyfriend (someone you knew for a while with whom you had an ongoing relationship)
    - Current steady girlfriend (someone you knew for a while with whom you have an ongoing relationship)
    - Past steady girlfriend (someone you knew for a while with whom you had an ongoing relationship)
    - Friend
    - Mother
    - Father
    - Other relative
    - Priest/clergy
    - Other (specify)

<u>**Life Events Survey (LES)**</u>

Listed below are events that sometimes bring about changes in people's lives. For each event, we need to know whether or not it happened to you **in the last six months**. If it did not happen to you, <u>**leave the rating scale blank**</u>. If it did happen to you, use the numbers to indicate its effect on your life. How much of a positive or negative impact did the event have on your life?

A rating of "-3" would indicate an extremely negative impact. A rating of "-2" would indicate that the impact was negative; a rating of -1 would indicate that the impact was slightly negative. A "0" indicates it had no impact, either positive or negative; "1" indicates the impact was slightly positive, a "2" that it was quite positive and a "3" indicates the impact was extremely positive.

Remember, if the event has NOT occurred in the last 6 months, LEAVE THE IMPACT SCALE BLANK. If the event HAS occurred in the last 6 months, mark a number between -3 and +3 to indicate its impact on your life.

Please just pick one number for each event (or leave blank if it didn't happen to you in the last 6 months up to today).

Impact on Your Life	*Extremely Negative*	*Negative*	*Slightly Negative*	*No Impact*	*Slightly Positive*	*Positive*	*Extremely Positive*
Marriage	-3	-2	-1	0	+1	+2	+3
Detention in jail or comparable institution	-3	-2	-1	0	+1	+2	+3
Death of spouse	-3	-2	-1	0	+1	+2	+3
Major change in sleeping habits (much more or much less sleep)	-3	-2	-1	0	+1	+2	+3
Death of close family member	-3	-2	-1	0	+1	+2	+3
Major change in eating habits (much more or much less food intake)	-3	-2	-1	0	+1	+2	+3
Foreclosure on mortgage or loan	-3	-2	-1	0	+1	+2	+3
Death of a close friend	-3	-2	-1	0	+1	+2	+3

Brief Symptom Inventory-18

Below is list of problems people sometimes have. Read each one carefully and circle the number of the response that best describes **HOW MUCH THAT PROBLEM HAS DISTRESSED OR BOTHERED YOU DURING THE PAST 7 DAYS INCLUDING TODAY.**

Circle only one number for each problem (0 1 2 3 4). Do not skip any items. If you change your mind, draw an X through your original answer and then circle your new answer.

HOW MUCH WERE YOU DISTRESSED OR BOTHERED BY THE ITEMS BELOW IN THE LAST 7 DAYS INCLUDING TODAY:

0 = Not at all 1 = A little bit 2 = Moderately 3 = Quite a bit 4 = Extremely

1. Faintness or dizziness	0	1	2	3	4
2. Feeling no interest in things	0	1	2	3	4
3. Nervousness or shakiness inside	0	1	2	3	4
4. Pains in heart or chest	0	1	2	3	4
5. Feeling lonely	0	1	2	3	4
6. Feeling tense or keyed up	0	1	2	3	4
7. Nausea or upset stomach	0	1	2	3	4
8. Feeling blue	0	1	2	3	4
9. Suddenly scared for no reason	0	1	2	3	4
10. Trouble getting your breath	0	1	2	3	4
11. Feelings of worthlessness	0	1	2	3	4
12. Spells of terror or panic	0	1	2	3	4
13. Numbness or tingling in parts of your body	0	1	2	3	4
14. Feeling hopeless about the future	0	1	2	3	4
15. Feeling so restless you couldn’t sit still	0	1	2	3	4
16. Feeling weak in parts of your body	0	1	2	3	4
17. Thoughts of ending your life	0	1	2	3	4
18. Feeling fearful.	0	1	2	3	4

Behavioral Complexity Scale (BCS)

http://gaincc.org/_data/files/Posting_Publications/Conrad_et_al_2011_BCS_Rasch_Report.pdf

The BCS is a scale of the General Individual Severity Scale (GISS). The GISS in turn is part of the larger Global Appraisal of Individual Needs (GAIN) which is actually a series of related instruments that share the same general instructions, questions (and variable names in most cases), scoring, interpretation, and clinical decision trees. The GAIN is a biopsychosocial assessment that integrates research and clinical practice to do diagnosis, placement, individualized treatment planning, and program evaluation and meets major reporting requirements.

The BCS "is a count of past-year symptoms related to externalizing disorders, including attention deficit, hyperactivity/impulsivity, and conduct disorder; it is based on the DSM_IV (American Psychiatric Association, 2000) symptoms/disorders that are common in adolescents but still persist into adulthood and are correlated with substance use severity (Dennis, Chan, & Funk, 2006).

The BCS consists of three subscales with a total of 33 items. Its subscales are the Inattentive Disorder Scale (IDS; 9 items), Hyperactivity-Impulsivity Scale (HIS; 9 items), and Conduct Disorder Scale (CDS; 15 items). **The item stem reads: "During the past 12 months, have you done the following things two or more times?" The response format is Yes/No (coded: no=0, yes=1).**

Items by Subscale:

INATTENTIVE DISORDER SCALE

1. Made mistakes because you were not paying attention.
2. Had a hard time paying attention at school, work or home.
3. Had a hard time listening to instructions at school, work or home.
4. Not followed instructions or not finished your assignments.
5. Had a hard time staying organized or getting everything done.
6. Avoided things that took too much effort, like school work or paperwork.
7. Lost things that you needed for school, work or home.
8. Been unable to pay attention when other things were going on.
9. Been forgetful or absentminded.

HYPERACTIVITY IMPULSIVITY SCALE

10. Fidgeted or had a hard time keeping your hands or feet still when you were supposed to.
11. Been unable to stay in a seat or where you were supposed to stay.
12. Felt restless or the need to run around or climb on things.
13. Gotten in trouble for being too loud when you were playing or relaxing.
14. Felt like you were always on the go or driven by a motor.
15. Talked too much or had others complain that you talked too much.
16. Gave answers before the other person finished asking the question.
17. Had a hard time waiting your turn.
18. Interrupted or butted into other people's conversations or games.

CONDUCT DISORDER SCALE

19. Been a bully or threatened other people.

20. Started fights with other people.
21. Used a weapon in fights.
22. Been physically cruel to other people.
23. Been physically cruel to animals.
24. Taken a purse, money or other things from a person by force.
25. Forced someone to have sex with you when they did not want to.
26. Set fires.
27. Broken windows or destroyed property.
28. Taken money or things from a house, building or car.
29. Lied or conned to get things you wanted or to have to avoid having to do something.
30. Taken things from a store or written bad checks to buy things.
31. Stayed out at night later than your parents or partner wanted.
32. Run away from home (partner) for at least one night.
33. Skipped work or school.

Patient-Reported Outcomes Measurement Information System PROMIS

Social Support: PROMIS Item Bank v2.0 - Companionship (Short Form 4a)

© 2008-2012 PROMIS Health Organization and PROMIS Cooperative

Please respond to each item by marking one box per row.	Rarely	Sometimes	Usually	Always	Never
Do you have someone with whom to have fun?	☐ 1	☐ 2	☐ 3	☐ 4	☐ 5
Do you have someone with whom to relax?	☐ 1	☐ 2	☐ 3	☐ 4	☐ 5
Do you have someone with whom you can do something enjoyable?	☐ 1	☐ 2	☐ 3	☐ 4	☐ 5
Can you find companionship when you want it?	☐ 1	☐ 2	☐ 3	☐ 4	☐ 5

PROMIS Item Bank v2.0 - Emotional Support (Short Form 4a)

Please respond to each item by marking one box per row. Never	Rarely	Sometimes	Usually	Always	Never
I have someone who will listen to me when I need to talk	☐ 1	☐ 2	☐ 3	☐ 4	☐ 5
I have someone to confide in or talk to about myself or my problems	☐ 1	☐ 2	☐ 3	☐ 4	☐ 5
I have someone who	☐ 1	☐ 2	☐ 3	☐ 4	☐ 5

makes me feel appreciated					
I have someone to talk with when I have a bad day	☐ 1	☐ 2	☐ 3	☐ 4	☐ 5

PROMIS Item Bank v2.0 - Informational Support (Short Form 4a)

**Please respond to each item by marking one box per row.**	**Rarely**	**Sometimes**	**Usually**	**Always**	**Never**
I have someone to give me good advice about a crisis if I need it	☐ 1	☐ 2	☐ 3	☐ 4	☐ 5
I have someone to turn to for suggestions about how to deal with a problem	☐ 1	☐ 2	☐ 3	☐ 4	☐ 5
I have someone to give me information if I need it	☐ 1	☐ 2	☐ 3	☐ 4	☐ 5
I get useful advice about important things in life	☐ 1	☐ 2	☐ 3	☐ 4	☐ 5

PROMIS Item Bank v2.0 - Instrumental Support (Short Form 4a)

**Please respond to each item by marking one box per row.**	**Rarely**	**Sometimes**	**Usually**	**Always**	**Never**
Do you have	☐	☐	☐	☐	☐

someone to help you if you are confined to bed?	1	2	3	4	5
Do you have someone to take you to the doctor if you need it?	☐ 1	☐ 2	☐ 3	☐ 4	☐ 5
Do you have someone to help with your daily chores if you are sick?	☐ 1	☐ 2	☐ 3	☐ 4	☐ 5
Do you have someone to run errands if you need it?	☐ 1	☐ 2	☐ 3	☐ 4	☐ 5

PROMIS Item Bank v2.0 - Social Isolation (Short Form 4a)

Please respond to each item by marking one box per row. Never	Rarely	Sometimes	Usually	Always	Never
I feel left out	☐ 1	☐ 2	☐ 3	☐ 4	☐ 5
I feel that people barely know me	☐ 1	☐ 2	☐ 3	☐ 4	☐ 5
I feel isolated from others	☐ 1	☐ 2	☐ 3	☐ 4	☐ 5
I feel that people are around me but not with me	☐ 1	☐ 2	☐ 3	☐ 4	☐ 5

Social Norms for Alcohol

- How many friends drink alcohol ≥3 times a week ("How many of your friends drink three or more times a week?")
- How many friends drink ≥5 drinks in one sitting ("How many of your friends have five or more drinks in one sitting?")
- How many would disapprove if you drank alcohol ≥3 times a week? ("How many of your friends would disapprove if you were to drink three or more times a week?")
- How many would disapprove if you drank ≥5 drinks in one sitting? ("How many of your friends would disapprove if you had five or more drinks in one sitting?")

STANDARD DRINK CONVERSION

When asked how much you drink in the following questions use this chart.

ONE STANDARD DRINK IS EQUAL TO:

Standard American BEER 12 oz. Can, Bottle or Glass
(3-5% alcohol)

Microbrew or European BEER 1/2 of a 12 oz. Can or Bottle (8%-12% alcohol)

WINE (12 – 17% alcohol) 4 oz. Glass

WINE Cooler 10 oz. Bottle

HARD LIQUOR 1-1/2 oz. or One Standard Shot
(80-proof, 40% alcohol)

HARD LIQUOR 1 oz.
(100-proof, 50% alcohol)
WINE: 1 Bottle
25 oz. (12 – 17% alcohol) = 5 standard drinks
40 oz. (12 – 17% alcohol) = 8 standard drinks

HARD LIQUOR: 1 Bottle
12 oz. = 8 standard drinks
25 oz. = 17 standard drinks
40 oz. = 27 standard drinks

<u>Daily Drinking Questionnaire-Revised</u>

Gender: Male_____ Female_____ **Height** _____' _____ " **Weight**________ lbs.
(Feet) (Inches)

INSTRUCTIONS FOR RECORDING DRINKING DURING A TYPICAL WEEK

IN THE CALENDAR BELOW, PLEASE FILL-IN YOUR DRINKING RATE AND TIME DRINKING DURING A **TYPICAL WEEK** IN THE LAST **30 DAYS.**

First, think of a *typical week* in the last *30 days you*. (Where did you live? What were your regular weekly activities? Where you working or going to school? Etc.) Try to remember as accurately as you can, *how much* and for *how long* you *typically drank* in a week during that one month period?

For each day of the week in the calendar below, fill in the **number of standard drinks typically consumed on that day** in the upper box and the **typical number of hours you drank** that day in the lower box. **Day of Week**	**Monday**	**Tuesday**	**Wednesday**	**Thursday**	**Friday**	**Saturday**	**Sunday**
**Number of *Drinks***							
**Number of *Hours Drinking***							

Stigma

I am going to read a list of statements that you may or may not agree with. For each statement, please tell me whether you **Strongly Agree, Agree, Disagree, or Strongly Disagree.**

1. You feel you are not as good a person as others because you have HIV.
   - Strongly Disagree
   - Disagree
   - Agree
   - Strongly Agree

2. Having HIV makes you feel unclean.
   - Strongly Disagree
   - Disagree
   - Agree
   - Strongly Agree

3. Most people think that a person with HIV is disgusting.
   - Strongly Disagree
   - Disagree
   - Agree
   - Strongly Agree

4. Having HIV makes you feel that you are a bad person.
   - Strongly Disagree
   - Disagree
   - Agree
   - Strongly Agree

5. Most people with HIV are rejected when others find out.
   - Strongly Disagree
   - Disagree
   - Agree
   - Strongly Agree

6. You are very careful who you tell that you have HIV.
   - Strongly Disagree
   - Disagree
   - Agree
   - Strongly Agree

7. You have been hurt by how people reacted to learning you have HIV.
   - Strongly Disagree
   - Disagree
   - Agree
   - Strongly Agree

8. You worry that people who know you have HIV will tell others.

Strongly Disagree
Disagree
Agree
Strongly Agree

9. You have stopped socializing with some people because of their reactions to your having HIV.

Strongly Disagree
Disagree
Agree
Strongly Agree

10. You have lost friends by telling them you have HIV.

Strongly Disagree
Disagree
Agree
Strongly Agree

NIH Toolbox Neurocognitive Measures

ONLY GIVEN at BASELINE AND 28 WEEK (7 MONTH) DATA COLLECTIONS

Flanker Task: NIH Toolbox Flanker Inhibitory Control and Attention Test

The Flanker task measures both a participant's attention and inhibitory control. The test requires the participant to focus on a given stimulus while inhibiting attention to stimuli (fish for ages 3-7 or arrows for ages 8-85) flanking it. Sometimes the middle stimulus is pointing in the same direction as the "flankers" (congruent) and sometimes in the opposite direction (incongruent). Scoring is based on a combination of accuracy and reaction time, and the test takes approximately 3 minutes to administer. This test is recommended for ages 3-85.

http://www.nihtoolbox.org/WhatAndWhy/Cognition/Attention/Pages/NIH-Toolbox-Flanker-Inhibitory-Control-and-Attention-Test.aspx

List Sorting Test: NIH Toolbox List Sorting Working Memory Test

This test requires immediate recall and sequencing of different visually and orally presented stimuli. Pictures of different foods and animals are displayed with accompanying audio recording and written text (e.g., "elephant"), and the participant is asked to say the items back in size order from smallest to largest, first within a single dimension (either animals or foods, called 1-List) and then on 2 dimensions (foods, then animals, called 2-List). The score is equal to the number of items recalled and sequenced correctly, and the test takes approximately 7 minutes to administer. This test is recommended for ages 7-85, but is available for use as young as age 3, if requested.

http://www.nihtoolbox.org/WhatAndWhy/Cognition/WorkingMemory/Pages/NIH-Toolbox-List-Sorting-Working-Memory-Test.aspx

Pattern Comparison Test: NIH Toolbox Pattern Comparison Processing Speed Test

This test measures speed of processing by asking participants to discern whether two side-by-side pictures are the same or not. Participants' raw score is the number of items correct in a 90-second period. The items are designed to be simple to most purely measure processing speed. The test overall takes approximately 3 minutes to administer. This test is recommended for ages 7-85, but is available for use as young as age 3, if requested.

http://www.nihtoolbox.org/WhatAndWhy/Cognition/ProcessingSpeed/Pages/NIH-Toolbox-Pattern-Comparison-Processing-Speed-Test.aspx

Hopkins Verbal Learning Test–Revised™ (HVLT-R™)

ONLY GIVEN at BASELINE AND 28 WEEK (7 MONTH) DATA COLLECTIONS

Purpose: Assess verbal learning and memory (immediate recall, delayed recall, delayed recognition)
Age range: 16 to 92 years
Admin: Individual
Admin time: 5-10 minutes with a 25-minute delay; 2 minutes to score
Qualification level: C
http://www4.parinc.com/Products/Product.aspx?ProductID=HVLT-R

PhenX Toolkit

ONLY GIVEN at BASELINE AND 28 WEEK (7 MONTH) DATA COLLECTIONS

Delayed Reward Discounting Task

The Monetary-Choice Questionnaire is a 27-item self-administered questionnaire. For each item, the participant chooses between a smaller, immediate monetary reward and a larger, delayed monetary reward. The protocol is scored by calculating where the respondent's answers place him/her amid reference discounting curves, where placement amid steeper curves indicates higher levels of impulsivity.

Monetary-Choice Questionnaire

For each of the next 27 choices, please indicate which reward you would prefer: the smaller reward today, or the larger reward in the specified number of days.

1. Would you prefer $54 today, or $55 in 117 days?

[ ] smaller reward today
[ ] larger reward in the specified number of days

2. Would you prefer $55 today, or $75 in 61 days?

[ ] smaller reward today
[ ] larger reward in the specified number of days

3. Would you prefer $19 today, or $25 in 53 days?

[ ] smaller reward today
[ ] larger reward in the specified number of days

4. Would you prefer $31 today, or $85 in 7 days?

[ ] smaller reward today
[ ] larger reward in the specified number of days

5. Would you prefer $14 today, or $25 in 19 days?

[ ] smaller reward today
[ ] larger reward in the specified number of days

6. Would you prefer $47 today, or $50 in 160 days?

[ ] smaller reward today
[ ] larger reward in the specified number of days

7. Would you prefer $15 today, or $35 in 13 days?

[ ] smaller reward today

[ ] larger reward in the specified number of days

8. Would you prefer $25 today, or $60 in 14 days?

[ ] smaller reward today
[ ] larger reward in the specified number of days

9. Would you prefer $78 today, or $80 in 162 days?

[ ] smaller reward today
[ ] larger reward in the specified number of days

10. Would you prefer $40 today, or $55 in 62 days?

[ ] smaller reward today
[ ] larger reward in the specified number of days

11. Would you prefer $11 today, or $30 in 7 days?

[ ] smaller reward today
[ ] larger reward in the specified number of days

12. Would you prefer $67 today, or $75 in 119 days?

[ ] smaller reward today
[ ] larger reward in the specified number of days

13. Would you prefer $34 today, or $35 in 186 days?

[ ] smaller reward today
[ ] larger reward in the specified number of days

14. Would you prefer $27 today, or $50 in 21 days?

[ ] smaller reward today
[ ] larger reward in the specified number of days

15. Would you prefer $69 today, or $85 in 91 days?

[ ] smaller reward today
[ ] larger reward in the specified number of days

16. Would you prefer $49 today, or $60 in 89 days?

[ ] smaller reward today
[ ] larger reward in the specified number of days

17. Would you prefer $80 today, or $85 in 157 days?

[ ] smaller reward today
[ ] larger reward in the specified number of days

18. Would you prefer $24 today, or $35 in 29 days?

[ ] smaller reward today
[ ] larger reward in the specified number of days

19. Would you prefer $33 today, or $80 in 14 days?

[ ] smaller reward today
[ ] larger reward in the specified number of days

20. Would you prefer $28 today, or $30 in 179 days?

[ ] smaller reward today
[ ] larger reward in the specified number of days

21. Would you prefer $34 today, or $50 in 30 days?

[ ] smaller reward today
[ ] larger reward in the specified number of days

22. Would you prefer $25 today, or $30 in 80 days?

[ ] smaller reward today
[ ] larger reward in the specified number of days

23. Would you prefer $41 today, or $75 in 20 days?

[ ] smaller reward today
[ ] larger reward in the specified number of days

24. Would you prefer $54 today, or $60 in 111 days?

[ ] smaller reward today
[ ] larger reward in the specified number of days

25. Would you prefer $54 today, or $80 in 30 days?

[ ] smaller reward today
[ ] larger reward in the specified number of days

26. Would you prefer $22 today, or $25 in 136 days?

[ ] smaller reward today
[ ] larger reward in the specified number of days

27. Would you prefer $20 today, or $55 in 7 days?

[ ] smaller reward today
[ ] larger reward in the specified number of days

https://www.phenxtoolkit.org/index.php?pageLink=browse.protocoldetails&id=530301

Balloon Analog Risk Task
The Balloon Analogue Risk Task (BART) is a computerized, laboratory-based test of risky behavior, which can be used with adolescents and adults. During the test, participants inflate, or pump up, a hypothetical balloon. Participants receive a certain amount of money for each pump of the balloon until it pops, at which point all money is lost. Participants can stop inflating the balloon at any point in the trial and collect the accrued money. Unlike other decision tasks that elicit choices between different discrete response options (with different scheduled penalty probabilities), the penalty probability in the Balloon Analogue Risk Task accelerates as a function of reward pursuit within the same behavioral option (such as alcohol drinking).

https://www.phenxtoolkit.org/index.php?pageLink=browse.protocoldetails&id=530501

NEURO-QOL

Neuro-QoL is a set of self-report measures that assesses the health-related quality of life (HRQOL) of adults and children with neurological disorders.

Neuro-QoL is comprised of item banks and scales that evaluate symptoms, concerns, and issues that are relevant across disorders - along with measures that assess areas most relevant for specific patient populations.

http://www.neuroqol.org/Pages/default.aspx

Prospective and Retrospective Memory Questionnaire (PRMQ)

REMEMBERING TO DO THINGS

In order to understand why people make memory mistakes, we need to find out about the kinds of mistakes people make, and how often they are made in normal everyday life. We would like you to tell us how often these kind of things happen to you. Please indicate by checking the appropriate box.

Please provide the following details about yourself.	Age_______	Male/Female_________
How many year of formal education have you had?		
Have you suffered from brain or head injury resulting in hospitalization (Y/N)		
Please give brief details		

Please answer all of the questions as accurately as possible.

	Very Often	Quite Often	Sometimes	Rarely	Never
Do you decide to do something in a few minutes' time and then forget to do it?	☐	☐	☐	☐	☐
Do you fail to recognize a place you have visited before?	☐	☐	☐	☐	☐
Do you fail to do something you were supposed to do a few minutes later even though it's there in front of you, like take a pill or turn off the kettle?	☐	☐	☐	☐	☐

	Very Often	Quite Often	Sometimes	Rarely	Never
Do you forget appointments if you are not prompted by someone else or by a reminder such as a calendar or diary?	☐	☐	☐	☐	☐
Do you fail to recognize a character in a radio or television show from scene to scene?	☐	☐	☐	☐	☐
Do you forget to buy something you planned to buy, like a birthday card, even when you see the shop?	☐	☐	☐	☐	☐

Do you fail to recall things that have happened to you in the last few days?	☐	☐	☐	☐	☐
Do you repeat the same story to the same person on different occasions?	☐	☐	☐	☐	☐
Do you intend to take something with you, before leaving a room or going out, but minutes later leave it behind, even though it's there in front of you?	☐	☐	☐	☐	☐
Do you mislay something that you have just put down, like a magazine or glasses?	☐	☐	☐	☐	☐
Do you fail to mention or give something to a visitor that you were asked to pass on?	☐	☐	☐	☐	☐
Do you look at something without realizing you have seen it moments before?	☐	☐	☐	☐	☐
If you tried to contact a friend or relative who was out, would you forget to try again later?	☐	☐	☐	☐	☐
Do you forget what you watched on television the previous day?	☐	☐	☐	☐	☐
Do you forget to tell someone something you had meant to mention a few minutes ago?	☐	☐	☐	☐	☐

Edited for American English, K. MacDonell (3/13/14)

Demographic Questionnaire

The following questions are about your background, education, living situation and health behaviors. Some of the questions are very personal, but remember everything you answer is confidential. Your name will not be associated with any of the information you give. There are no right or wrong answers, so please respond as honestly as possible.

1. How old are you?
   I want to confirm your current age. The number you entered is displayed on the screen. Is that correct?
   - Yes
   - No
2. What is your current gender identity?
   - Male
   - Female
   - Trans male/Trans man
   - Trans female/Trans woman
   - Genderqueer/Gender non-conforming
   - Different identity (please state): _____
   - Decline to State
3. What sex were you assigned at birth, meaning on your original birth certificate?
   - Male
   - Female
4. Are you of Hispanic (Spanish) or Latino heritage?
   - Yes
   - No
     - If yes: Central America
       - Cuban
       - Dominican
       - Mexican, Mexican American or Chicano
       - Puerto Rican
       - Mixed Hispanic Background
       - South American
       - Other: _________________
5. In addition to knowing whether or not you are of Hispanic/Latino ethnic heritage, what is your racial background?
   - Asian/Pacific Islander
   - Black/African American
   - Native American/Alaskan Native
   - White
   - Mixed Race
   - Other: __________
6. What is your current zip code?
7. What language do you usually speak at home?
   - English
   - Spanish
   - Other:___________
8. What is your current marital status?
   - Single
   - Living with steady partner
   - Married

Separated
Divorced
Widowed
Other:____________

9. Are you in school these days? And "school" could mean a school or program where you are working toward a high school diploma, GED, or college/technical degree.
   - Yes
   - No
   - No, I have graduated
   - Yes, but I am on summer/winter/spring break now
10. What is the highest level of education or grade you have completed?
   - Eighth grade or less
   - More than eighth grade but not complete high school
   - High school graduate
   - GED
   - Some college/technical education
   - Technical school graduate
   - College graduate
   - Some graduate school
   - Master's degree or above
   - None, no formal schooling
11. Are you currently receiving or have you ever received special education services?
   - Yes
   - No
12. Did you repeat any grades in school?
   - Yes
   - No

   If yes, what grade(s) did you repeat: _______________
13. Are you currently employed?
   - Yes
   - No

   If yes, are you employed full-time or part-time?
   - Full-time
   - Part-time
14. How much money did you make altogether during the past 30 days?
   - None or Less than $50 (e.g., less than $600 a year)
   - $51-$249 (e.g., $600 - $2,999 a year)
   - $250-$499 (e.g., $3,000 - $5,999 a year)
   - $500-$999 (e.g., $6,000 - $11,999 a year)
   - $1,000 - $2,999 (e.g., $12,000 - $35,999 a year)
   - $3,000 - $4,999 (e.g., $36,000 - $59,999 a year
   - $5,000 or more (e.g., $60,000 or more a year)
   - Rather not answer
   - Don't know
15. Do you own or have access to any of the following? By having access, we mean that you can use these to communicate with your health care team. Choose all that apply.
   - Cell phone
   - Pager
   - Computer with internet access
   - Email
   - None of these

16. Where are you currently living or staying most of the time?
    - Your own house or apartment
    - At your parents' house or apartment
    - At another family member(s) house or apartment
    - At a non-family member's house or apartment
    - Foster home or group home
    - In a rooming, boarding, hallway house, or a shelter/welfare hotel
    - On the street(s) (vacant lot, abandoned building, park, etc.)
    - Some other place not mentioned: __________________
17. How do you identify?
    - Straight
    - Gay
    - Lesbian
    - Queer
    - Bisexual
    - Questioning
    - Other: _______________________
18. Would you use any of the following terms to describe yourself? Choose all that apply.
    - Down low (DL)
    - Same-gender loving (SGL)
    - Dyke
    - Queer
    - Trade
    - Two-spirited
    - Homothug
    - Other:___________________________
    - None of these
19. How old were you when you found out you were HIV positive?
    - I want to confirm how old you were when you found out you were HIV positive. The number you entered is displayed on the screen. Is that correct?
        - Yes
        - No
20. How do you think you got HIV?
    - I was born with it.
    - Use of blood products (blood, factor, etc.)
    - Sex with a man
    - Sex with a woman
    - Injection drug use (e.g., injecting into a vein, muscle, or under your skin (skin-popping) not including drugs prescribed by your doctor to treat a medical condition
    - Sex with a man and injection drug use
    - Other:_____________________________
    - Don't know

21. Do you have biological children?
    - Yes
    - No

    How many biological children do you have?
22. Do you have adopted children?
    - Yes
    - No

    How many adopted children do you have?

Geocoding

Geocoding is used to collect data on environment (along with demographic questionnaire). The current ATN geocoding consultants at Columbia University will develop the following variables: alcohol outlets, neighborhood disadvantage, and public transportation. Location of alcohol consumption is collected through the TLFB procedure described above. Zip code information will also be collected from the Demographics.

2. Process and Cost Effectiveness Measures

The study includes a cost effectiveness analysis via two components: 1) cost analysis of the intervention, and 2) an incremental cost effectiveness analysis that compares the value of the home vs. clinical based treatment conditions.

A modification of The Drug Abuse Treatment Cost Analysis Program (DATCAP), combined with study contact and expenditure records, is used to estimate the cost of the each treatment condition. The DATCAP is a standardized data collection instrument that estimates the economic cost of alcohol treatment programs. Administration of the DATCAP is generally a collaborative effort involving an economist and various members of the intervention staff (administrators, therapist and accounting/finance personnel). The DATCAP organizes program resources into the following categories: personnel, buildings and facilities, supplies and materials, and miscellaneous resources including the value of donated or subsidized items. Client case flow data is incorporated to determine the average annual cost per client for each service type. Other useful computations include weekly cost per client, average cost per intervention episode (based on length of stay in the program), and marginal cost per contact.

CHWs maintain the Implementation Log and Contact Logs for all time spent on the patient-related activities including (calls, contacts, and missed appointments). Via chart review, we obtain information about frequency of clinic visits, appointment history, and contact with CHW during those visits throughout the study to determine potential "dose" of MI beyond the 4-session MET intervention. Both CHWs and supervisors maintain training logs to document all time spent on MI implementation activities.

Implementation Log Sample:

Date	Category	Problem/Issue	Process Notes	Resolution	Best Practices
2/13/13	Equipment	All devices must be re-encrypted with more secure software. Thus, all PATH devices in the field need to be brought into IT. This could potentially disrupt assessment schedules.	As of 4/8/13, we are still waiting for notice to bring in laptops for encryption	We will bring devices in on a set schedule, always leaving one assessment laptop so that assessments do not need to be disrupted.	Continue to move forward with assessments until IT indicates that we need to bring computers in for re-assessment.

Motivational Interviewing Treatment Integrity ("MITI") Coding

MITI coding form:

Project: ____________________ **Site**: ____________________ **Date Coded**: __________

MITI 3.1
Coding Sheet

Tape: ____________ **Therapist:** __________ **Session Date:** ______ **Coder:** __________

Segment Start: ______________ **Segment End:** ______

Global Ratings	1	2	3	4	5	SCORE
Empathy						
Direction						
Collaboration						
Evocation						
Autonomy/Support						

Behavior Counts			
**Giving Information**			0
**MI Adherent (MiA)**	Asking permission, affirming client, emphasizing control, offering support		0
**MI Non-adherent (MiNa)**	Advise, confront, direct		0
**Questions**	**Closed Question (CQ)**		0
	**Open Question (OQ)**		0
		TOTAL QUESTIONS (Q)	**0**
**Reflections**	**Simple (Rs)**		0
	**Complex (Rc)**		0
		TOTAL REFLECTIONS (RF)	**0**

First sentence:
Last sentence:
(Please indicate client or therapist)

**% Complex Reflections (%CR)**	0.00%	**% Open Question (%OC)**	0.00%
**Reflection-Question Ratio (R:Q)**	0.00	**Average MI Spirit**	
**% MI Adherent (%MiA)**	0.00%		

Project: 0 **Site**: 0 **Date Coded**: 1/0/1900

MITI Competence Summary
(*Please use information from page 1*)

	Solid Competence	Beginning Proficiency	Below Proficiency	Result
Global Spirit	☐ ≥ 4	☐ ≥ 3.5	☐ < 3.5	
Global Empathy	☐ ≥ 4	☐ ≥ 3.5	☐ < 3.5	
Global Direction	☐ ≥ 4	☐ ≥ 3.5	☐ < 3.5	
Reflection/Question Ratio	☐ ≥ 2	☐ ≥ 1	☐ < 1	0.00
% Open Question	☐ ≥ 70%	☐ ≥ 50%	☐ < 50%	0.00%
% Complex Reflections	☐ ≥ 50%	☐ ≥ 40%	☐ < 40%	0.00%
% MI Adherent	☐ ≥ 90%	☐ ≥ 80%	☐ < 80%	0.00%
Client/therapist talk time	☐ Majority client speech	☐ Approx. equal	☐ Majority therapist	☐ N/A

Coder Notes:

Evidence Based Practice Attitudes Scale (EBPAS)

The following questions ask about your feelings about using new types of therapy, interventions, or treatments. Manualized therapy refers to any intervention that has specific guidelines and/or components that are outlined in a manual and/or that are to be followed in a structured/predetermined way.

Fill in the circle indicating the extent to which you agree with each item using the following scale:
0 Not at All
1 To a Slight Extent
2 To a Moderate Extent
3 To a Great Extent
4 To a Very Great Extent

1. I like to use new types of therapy/interventions to help my clients………………………..
2. I am willing to try new types of therapy/interventions even if I have to follow a treatment manual…………………………………………………………………………
3. I know better than academic researchers how to care for my clients.……………….……..
4. I am willing to use new and different types of therapy/interventions developed by researchers……………………………………………………………………………….
5. Research based treatments/interventions are not clinically useful…………………….………
6. Clinical experience is more important than using manualized therapy/treatment…………..
7. I would not use manualized therapy/interventions……………………………………….
8. I would try a new therapy/intervention even if it were very different from what I am used to doing……………………………………………………………………………......

For questions 9-15: If you received training in a therapy or intervention that was new to you, how likely would you be to adopt it if:

9. it was intuitively appealing?...…………………………………………….…………………
10. it "made sense" to you?…………………………………………………………………
11. it was required by your supervisor?…………………………………………………….
12. it was required by your agency?……………….……………………………………….
13. it was required by your state?…………………….…………………………………….
14. it was being used by colleagues who were happy with it?……………………………..
15. you felt you had enough training to use it correctly?……………………………………..

SID:
Site Number:
Session Date:
Coding Date:
Rater:

Coaching Tool

Version 1/29/14

<u>Motivational Interviewing Items</u>

1. The counselor cultivates empathy and compassion with client(s).	1	2	3	4
2. The counselor fosters collaboration with client(s).	1	2	3	4
3. The counselor supports autonomy of client(s).	1	2	3	4
4. The counselor works to evoke client(s)'ideas and motivations for change	1	2	3	4
5. The counselor balances the client's agenda with focusing on the target behaviors.	1	2	3	4
6. The counselor demonstrates reflective listening skills.	1	2	3	4
7. The counselor uses reflections strategically.	1	2	3	4
8. The counselor reinforces strengths and positive behavior change	1	2	3	4
9. The counselor uses summaries effectively.	1	2	3	4
10. The counselor asks questions in an open-ended way.	1	2	3	4
11. The counselor solicits feedback from client(s)	1	2	3	4
12. Is resistance or ambivalence present in the session? If yes - The counselor addresses client(s)' resistance/ ambivalence	1	2	3	4

Target Behavior(s) of Session:

Notes:

3. Intervention and Study Evaluations

Patient Satisfaction Questionnaire (PSQ)-18

The Patient Satisfaction Questionnaire (PSQ), consisting of 80 items, was originally developed by Ware and his colleagues (Ware, Snyder, and Wright, 1976 a, b; see “Related Reading” below). A more recent version of the questionnaire is the PSQ-III, available below. The PSQ-III is a 50-item survey that taps global satisfaction with medical care as well as satisfaction with six aspects of care: technical quality, interpersonal manner, communication, financial aspects of care, time spent with doctor, and accessibility of care. A memo on scoring the PSQ-III is also available below. It provides background information, results of psychometric analyses, and scoring rules for measures constructed from the periodic satisfaction surveys.

The PSQ-18 is a short form version that retains many characteristics of its full-length counterpart. The PSQ sub-scales show acceptable internal consistency reliability. Furthermore, corresponding PSQ-18 and PSQ-III subscales are substantially correlate with one another. The PSQ-18 may be appropriate for use in situations where the need for brevity precludes administration of the full-length PSQ-III. The PSQ-18 takes approximately 3-4 minutes to complete.

http://www.rand.org/content/dam/rand/www/external/health/surveys_tools/psq/psq18_survey.pdf

Client Experience of Motivational Interviewing scale (CEMI)

Client Evaluation of Counseling

Please rate each response on the scale below relating to your **most recent session** with your counselor.

1	2	3	4
**Not At All**	**Only a Little**	**Some**	**A Great Deal**

In your session, how much did your counselor:

	Not at All	Only a Little	Some	A Great Deal
1. Focus only on your weaknesses.	1	2	3	4
2. Help you to talk about changing your behavior.	1	2	3	4
3. Act as a partner in your behavior change.	1	2	3	4
4. Helped you to discuss your need to change your behavior.	1	2	3	4
5. Make you feel distrustful of him/her	1	2	3	4
6. Help you examine the pros and cons of changing your behavior.	1	2	3	4
7. Help you to feel hopeful about changing your behavior.	1	2	3	4
8. Argue with you to change your behavior.	1	2	3	4
9. Change the topic when you became upset about changing your behavior.	1	2	3	4
10. Push you forward when you became unwilling to talk about an issue further.	1	2	3	4
11. Act as an authority on your life.	1	2	3	4
12. Tell you what to do.	1	2	3	4
13. Argue with you about needing to be 100% ready to change your behavior.	1	2	3	4
14. Show you that she/he believes in your ability to change your behavior.	1	2	3	4
15. Help you feel confident in your ability to change your behavior	1	2	3	4
16. Help you recognize the need to change your behavior.	1	2	3	4

Thank you for your time. Your feedback is valuable. Please return this form in the folder provided to your counselor.

APPENDIX IV: CASE REPORT FORMS (“CRFs”)

1. Study Screening Log
2. Screening and Eligibility Form
3. Locator Form
4. Virology Results Form
5. Nail Sample Form
6. Off Study Form
7. Premature Discontinuation From Study Intervention Form
8. Visit Report Form
9. Monitoring Untoward Event Form

ATN 129
STUDY SCREENING LOG

SITE CODE: |___|___|

VISIT: SCREENING

INSTRUCTIONS: Use this Screening Log to record all individuals approached to consider consenting for ATN 129 participation, whether or not consent was given. Initial and date each entry. Enter new data since the Screening Log was previously transmitted in Remote Data Capture (RDC) by the first Friday of each month for those not enrolled only. **When study accrual ends, obliterate the names/initials and dates of birth belonging to individuals who did not give consent.** Store the Screening Log in a double-locked area accessible to study staff only.

[1]**AGE** ***(Record age at time individual was first considered for participation in the study, i.e., when approached to consent. If age is unknown, record 'UNK'. If age is not reported or not asked, record 'NR'.)***

[2]**BIRTH GENDER** ***(Record only one. If birth gender is unknown, record 'UNK'. If birth gender is not reported or not asked, record 'NR'.)***

1= Male	2= Female

[3]**HISPANIC/LATINO ORIGIN** ***(Record only one)***

0=Non-Hispanic or Non-Latino	1=Hispanic or Latino	8=Subject does not know	9=Subject does not want to report	10=Information not available to clinic or not asked

[4]**RACE** ***(Enter all that apply)***

1= American Indian<br>2= Alaska Native<br>3= Asian<br>4= Native Hawaiian	5= Pacific Islander (other than Native Hawaiian)<br>6= Black or African American<br>7= White	8= Subject does not know<br>9= Subject does not want to report<br>10= Information not available to clinic or not asked

[5]**REASONS NOT CONSENTED** ***(Record all that apply)***

1=Not interested in the study<br>2=Too time consuming/demanding<br>3=Afraid of loss of confidentiality<br>4=No direct benefit from participating<br>9=Does not want to report reason	10= Information not available to clinic or not asked<br>11=Does not want to undergo intervention 1<br>12=Does not want to undergo intervention 2<br>13=Does not want to undergo intervention 3<br>14=Does not want to undergo intervention 4	99=Other, specify:

[6]**REASONS INELIGIBLE** ***(Record all that apply)***

1=Ineligible age<br>2=Ineligible gender<br>3=Ineligible race/ethnicity<br>4=No documentation of HIV infection<br>5=ART requirement not met<br>6=HIV VL requirement not met<br>7=CD4 requirement not met<br>8=Presence of Grade 3 or higher clinical/laboratory toxicity<br>9=Presence of serious/acute/chronic condition that would interfere with study intervention/outcomes<br>10=Pregnancy (currently or within the past X weeks)	11=Breastfeeding<br>12=Lactating (currently or within the past X weeks)<br>13=Received/needs to receive disallowed medications, specify medication(s) name<br>14=Presence of serious psychiatric symptoms or thought disorder<br>15=Visibly distraught, suicidal, homicidal, etc.<br>16=Intoxicated or under the influence of alcohol or other substances<br>50= Not infected with HIV by behavioral means *or* infected before age 9 years old<br>For other protocol-specific reasons, use higher #s except #s 81, 82, 83 and 99<br>99=Other, specify

[7]**REASONS NOT ENROLLED WHEN ELIGIBLE** ***(Record all that apply)***

81=Unable to locate or contact	82=Withdrew consent	83=Unwilling to comply with all study requirements	99=Other, specify

SITE CODE: |___|___|

#	*DO NOT ENTER IN QUALTRICS* NAME OR INITIALS OF INDIVIDUAL	*DO NOT ENTER IN QUALTRICS* DATE OF BIRTH (mm-dd-yyyy)	AGE (YR)[1]	BIRTH GENDER[2]	HISP/ LAT ORIGIN[3]	RACE[4]	IF APPROACHED, BUT NOT CONSENTED, PROVIDE REASON(S)[5] STOP *RECORD 'NA' IF CONSENTED*	DATE CONSENTED (mm-dd-yyyy)	PID	DATE SCREENED (mm-dd-yyyy)	REASON(S) INELIGIBLE[6] STOP *RECORD 'NA' IF ELIGIBLE*	REASON(S) NOT ENROLLED WHEN ELIGIBLE[7] *RECORD 'NA' IF ENROLLED*	*DO NOT ENTER IN QUALTRICS* *STAFF INITIALS AND DATE*	*DO NOT ENTER IN QUALTRICS* DATE DATA TRANSMITTED *RECORD 'NA' IF ENROLLED*
1.														
2.														
3.														
4.														
5.														
6.														
7.														
8.														
9.														
10.														

SITE CODE: |___|___|

#	*DO NOT ENTER IN QUALTRICS* NAME OR INITIALS OF INDIVIDUAL	*DO NOT ENTER IN QUALTRICS* DATE OF BIRTH (mm-dd-yyyy)	AGE (YR)[1]	BIRTH GENDER[2]	HISP/ LAT ORIGIN[3]	RACE[4]	IF APPROACHED, BUT NOT CONSENTED, PROVIDE REASON(S)[6] STOP *RECORD 'NA' IF CONSENTED*	DATE CONSENTED (mm-dd-yyyy)	PID	DATE SCREENED (mm-dd-yyyy)	REASON(S) INELIGIBLE[5] STOP *RECORD 'NA' IF ELIGIBLE*	REASON(S) NOT ENROLLED WHEN ELIGIBLE[7] *RECORD 'NA' IF ENROLLED*	*DO NOT ENTER IN QUALTRICS*	
													STAFF INITIALS AND DATE	DATE DATA TRANSMITTED *RECORD 'NA' IF ENROLLED*
__.														
__.														
__.														
__.														
__.														
__.														
__.														
__.														
__.														
__.														
__.														

SITE CODE: |___|___|

#	*DO NOT ENTER IN QUALTRICS* NAME OR INITIALS OF INDIVIDUAL	*DO NOT ENTER IN QUALTRICS* DATE OF BIRTH (mm-dd-yyyy)	AGE (YR)[1]	BIRTH GENDER[2]	HISP/ LAT ORIGIN[3]	RACE[4]	IF APPROACHED, BUT NOT CONSENTED, PROVIDE REASON(S)[6] STOP *RECORD 'NA' IF CONSENTED*	DATE CONSENTED (mm-dd-yyyy)	PID	DATE SCREENED (mm-dd-yyyy)	REASON(S) INELIGIBLE[5] STOP *RECORD 'NA' IF ELIGIBLE*	REASON(S) NOT ENROLLED WHEN ELIGIBLE[7] *RECORD 'NA' IF ENROLLED*	*DO NOT ENTER IN QUALTRICS* *STAFF INITIALS AND DATE*	*DO NOT ENTER IN QUALTRICS* DATE DATA TRANSMITTED *RECORD 'NA' IF ENROLLED*
__.														
__.														
__.														
__.														
__.														
__.														
__.														
__.														
__.														
__.														
__.														

Use copies of this page if necessary and number each copy in sequence.

Seq. No. I_____I ***(Record "1" if this is the original page of this form. Designate subsequent copies of this page with a 2, 3, etc.)***

ATN 129

SCREENER & ELIGIBILITY FORM

RECRUITMENT ID: |___|___|___|___|___|___|___| **INTERVIEWER ID:** |___|___|___|___|

SITE CODE: |___|___| **VISIT DATE:** |___|___|-|___|___|-|___|___|___|___|
Month Day Year

VISIT: Pre-entry

Completed by (please circle): In-Person By Telephone

INTERVIEW INTRODUCTION [INTERVIEWER READ]

Hi, my name is ____________________. I would like to ask you a few questions to find out a little bit about you. This information will only be used to determine if you qualify for our study. Please remember that there are no right or wrong answers and that all of your responses are confidential - meaning we will not tell anyone (parents, teachers, etc.) your answers. We will use a study number instead of your name to protect your information and only authorized research staff will have access to your information.

Your participation in this research study, including answering any of the following questions, is completely voluntary. Some of the questions may make you feel uncomfortable; you can refuse to answer any of the following questions or decide that you do not want to take part in the study at any time. If you have questions about this research study, you can contact (insert name and contact for site PI and contact information for IRB). I have this information here on a card, in case you have any questions.

Please take as much time as you need to answer these questions so that I can collect information that is as accurate as possible. It should take about 5 to 10 minutes to answer the questions. Now, we would like to ask for your verbal permission to ask you some questions about yourself. Are you willing to answer some questions to find out if you qualify for the study?

[INTERVIEWER]: If response is yes, continue with screening interview. If no, ask for reasons why:

- ☐ No interest 01
- ☐ Worried about anonymity 02
- ☐ Study takes too long 03
- ☐ Afraid of research/guinea-pig 04
- ☐ Rather not say 05
- ☐ Other 06

If other, specify ________________________________

Thank the participant for his/her time.

ATN 129

SCREENER AND ELIGIBILITY FORM

RECRUITMENT ID: |___|___|___|___|___|___|___| **INTERVIEWER INITIALS:** |___|___|___|___|

SITE CODE: |___|___| **VISIT DATE:** |___|___|-|___|___|-|___|___|___|___|
Month Day Year

VISIT: Pre-entry

Completed by (please circle): In-Person By Telephone

INSTRUCTIONS: Obtain verbal consent PRIOR to completing the Screener and Eligibility Form. This form is entered into Qualtrics. A hard copy of the study Screener Survey form for each participant must be kept on file at the site but separate from participant research records and also uploaded into Dropbox within 24 hours of completion.

1. **Date of Verbal Consent:** |____|____|-|____|____|-|____|____|____|____|

2. What is your age? |____|____|

   Birthdate |____|____|-|____|____|-|____|____|____|____|
   Month Day Year

   (Confirm with birthdate)
   If between 16.0 to 24.11 years, continue to Question 3,
   If 15 years or under OR 25 years or older, then Not eligible, discontinue screening interview

3. Does patient receive services at one of the study ATN clinics?

   ☐ YES
   ☐ NO (Not eligible, discontinue screening interview)

4. Has HIV-1 infection been documented in the participant's medical record*?

   ☐ Positive ***(Confirm with documented test results)***

   *by at least one of the following criteria:

   - Reactive HIV screening test result with an antibody-based, Food and Drug Administration (FDA)-licensed assay followed by a positive supplemental assay (e.g., HIV-1 Western Blot, HIV-1 indirect immunofluorescence);
   - Positive HIV-1 deoxyribonucleic acid (DNA) polymerase chain reaction (PCR) assay;
   - Plasma HIV-1 quantitative ribonucleic acid (RNA) assay >1,000 copies/mL; or
   - Positive plasma HIV-1 RNA qualitative assay

   ☐ Negative ***(Not eligible, discontinue screening interview)***
   ☐ Unknown ***(Not eligible, discontinue screening interview)***

5. Do you plan on staying in this geographical area over the next 12 months?

☐ Yes
☐ No ***(Not eligible, discontinue screening interview if there are solid plans for moving within study period)***

6. Are you currently involved in any other behavioral research (assessment or intervention) studies targeting adherence or alcohol use or substance abuse treatment programs or neurocognitive research studies?

☐ Yes ***(Please submit a Query for ATN 129)***
If Yes, which Protocol(s)? ____

☐ No

7. Have you been prescribed HIV medications or been told by your physician you should be on HIV medications (whether you take them or not)?
☐ Yes
☐ No

8. In the last 12 weeks (three months), have you used any alcohol?

☐ Yes
☐ No ***(Not eligible, discontinue screening interview)***

9. Do you have a detectable HIV viral load, within the past 4 weeks?

☐ Yes (Please confirm with documented test results from medical chart)
☐ No ***(Not eligible, discontinue screening interview)***

What is your last HIV viral load? _____________________ (has to be in the detectable range)

Date of your last HIV viral load? _____________________ (has to be within the past 4 weeks)

INTERVIEWER ONLY: ***[To be completed immediately after you have asked the participant all of the questions in the Screener Form].*** *To be considered eligible for enrollment, an individual must NOT meet any of the criteria listed below (no response to each). Please circle one response for each item.*

10. Does the patient have a thought disorder (psychosis) or intellectual disability that would impair their ability to provide true informed consent/assent and/or interfere with completing the study (**NOTE:** NO exclusions will be made due to co-morbid mental health problems, i.e., ADHD, conduct disorder, depression, anxiety disorder)?

11. Based on the participant's ability to understand and answer the questions read to them in English, do you think that this participant will be able to provide informed consent/assent to participate in this study and take part in English (data collection and intervention)?

☐ Yes
☐ No ***(Not eligible)***

12. Is the patient intoxicated or under the influence of alcohol or other substances at the time of consent/assent that would impair the individual's ability to provide true informed consent/assent and/or interfere with the study's objectives?

☐ Yes ***(Not eligible)***
☐ No

CONTINUE WITH NEXT SECTION

INSTRUCTIONS: *If any of the above ineligible responses are chosen, read the INELIGIBLE section. If no ineligible responses are chosen, read the ELIGIBLE section below.*

FOR INELIGIBLE PARTICIPANTS:

Participants for this research project are selected based on the questions you were just asked. Based on your answers, it turns out you're not eligible to participate at this time, but you can complete this screener again the next time I see you. Thank you for taking the time to speak with me about our study.

FOR ELIGIBLE PARTICIPANTS:

Thank you very much for the information you provided. Based on your answers to these questions, you are eligible to participate in this research study. That means that if you want to participate in the research, you may. Do you think you might be interested in taking part in this research study?

RESPONSE:

If no; thank them for their time.

If yes; say**, before we go any further, I need to review an informed consent document with you.**

CONDUCT THE INFORMED CONSENT PROCESS

Investigator/Designee:

________________________ ________________________ ____________

Signature Print Name Date

If participant enrolls:

Participant Name: ________________________ Date of Birth: |___|___|-|___|___|-|___|___|___|___|

Month Day Year

Place this form in the participant's individual research file and upload into Dropbox within 24 hours of completion.

ATN 129
LOCATOR FORM

INSTRUCTIONS: This form should be completed by a research staff member with the help of the participant after all contacts (i.e., Study Screening, Baseline Data Collection, MET Sessions, all Follow-Up Data Collections)

Tell the participant:

If first time completing Locator Form: "Thank you for agreeing to participate in this study. If it is ok with you, I would like you to provide me with information that will help us get in touch with you later. This information is only to help us contact you so that we can schedule an appointment and/or remind you about your appointment. If possible, could you also give me the names of other people who I may contact in case I cannot reach you? These should be people who usually know how to reach you. We will keep this form under double locks and separate from any other research records so that your contact information stays private. Only the study staff at our site can access this information."

Not the first time completing Locator Form: "Thank you for your time, if it is ok with you, I would like you to provide me with your most up-to-date information that will help us stay in touch with you during this study. This information is only to help us contact you so that we can schedule an appointment and/or remind you about your appointment. If possible, could you also give me the names of other people who I may contact in case I cannot reach you? These should be people who usually know how to reach you. We will keep this form under double locks and separate from any other research records so that your contact information stays private. Only the study staff at our site can access this information."

1) Full Name:

____

First Middle Last

2) What are your nicknames? (What do your friends call you?)

(1) ________________

(2) _________________

3) Where do you currently live?

_______________________________ __________

Street Apt. #

__________________ __________ __________

City State Zip Code

3a) Can we send mailings (e.g., reminder letters) to this address? ☐ Yes ☐ No

3b) Do you live anywhere else or spend several nights a week at another place? ☐ Yes ☐ No

If yes, can we contact you there? ☐ Yes ☐ No

ATN 129
LOCATOR FORM

Street address of Secondary contact location:

____ ____________
Street Apt. #

____________________________________ ____________
City State Zip Code

Home Phone Number: (____)__________________<br>☐ Refused / NA / Unknown<br>Best days/times to call: ***(Circle all days that apply)***<br>Weekends: Sa Su<br>Btw ____:____ AM/PM & ____:____ AM/PM<br>Weekdays: M Tu W Th F<br>Btw ____:____ AM/PM & ____:____ AM/PM	Can we call you at **home**?	☐ Yes ☐ No
	If yes, can we leave a message?	☐ Yes ☐ No
	If yes, what message should we leave?	__________<br>__________<br>__________<br>__________
	How should we identify ourselves?	__________
Work Phone Number: (____)__________________<br>☐ Refused / NA / Unknown<br>Best days/times to call: ***(Circle all days that apply)***<br>Weekends: Sa Su<br>Btw ____:____ AM/PM & ____:____ AM/PM<br>Weekdays: M Tu W Th F<br>Btw ____:____ AM/PM & ____:____ AM/PM	Can we call you at **work**?	☐ Yes ☐ No
	If yes, can we leave a message?	☐ Yes ☐ No
	If yes, what message should we leave?	__________<br>__________<br>__________<br>__________
	How should we identify ourselves?	__________
Cell Phone Number: (____)__________________<br>☐ Refused / NA / Unknown	Can we call your cell phone and leave a voice message?	☐ Yes ☐ No
	If yes, what message should we leave?	__________<br>__________<br>__________<br>__________

	If no, can we send a text message? ☐ Yes ☐ No
	If yes, what message should we send? ________________ ________________ ________________ ________________
E-mail address: ____________________________<br>☐ Refused / NA / Unknown	Can we email you? ☐ Yes ☐ No
	If yes, what message should we send? ________________ ________________ ________________ ________________

Additional comments or updates and/or order of preference of contact method, if necessary:

____

____

CONTACT #1

☐ Refused / NA / Unknown

Name:

____
First Middle Last

Relationship to you? ________________________________

Home Phone Number: (____)__________________<br>☐ Refused / NA / Unknown<br>Best days/times to call: ***(Circle all days that apply)***<br>Weekends: Sa Su<br>Btw |___|___|:|___|___|AM/PM & |___|___|:|___|___| AM/PM<br>Weekdays: M Tu W Th F<br>Btw |___|___|:|___|___|AM/PM & |___|___|:|___|___| AM/PM	Can we call this person at home? ☐ Yes ☐ No
	If yes, can we leave a message? ☐ Yes ☐ No
	If yes, what message should we leave? ________________ ________________ ________________ ________________
	How should we identify ourselves? ________________

Work Phone Number: (____)________________<br>☐ Refused / NA / Unknown<br>Best days/times to call: ***(Circle all days that apply)***<br>Weekends: Sa Su<br>Btw ___ ___ : ___ ___ AM/PM & ___ ___ : ___ ___ AM/PM<br>Weekdays: M Tu W Th F<br>Btw ___ ___ : ___ ___ AM/PM & ___ ___ : ___ ___ AM/PM	Can we call this person at work? ☐ Yes ☐ No<br>If yes, can we leave a message? ☐ Yes ☐ No<br>If yes, what message should we leave? ________________<br>How should we identify ourselves? ________________

Cell Phone Number: (____)________________<br>☐ Refused / NA / Unknown	Can we call this person's cell phone and leave a voice message? ☐ Yes ☐ No<br>If yes, what message should we leave? ________________<br>If no, can we send a text message? ☐ Yes ☐ No<br>If yes, what message should we send? ________________
E-mail address: ____________________________<br>☐ Refused / NA / Unknown	Can we email this person? ☐ Yes ☐ No<br>If yes, what message should we send? ________________

CONTACT #2

☐ Refused / NA / Unknown

Name:

____

First Middle Last

Relationship to you? ____________________________

Home Phone Number: (____)________________<br>☐ Refused / NA / Unknown<br>Best days/times to call: ***(Circle all days that apply)***<br>Weekends: Sa Su<br>Btw __|__ : __|__ AM/PM & __|__ : __|__ AM/PM<br>Weekdays: M Tu W Th F<br>Btw __|__ : __|__ AM/PM & __|__ : __|__ AM/PM	Can we call this person at home? ☐ Yes ☐ No<br>If yes, can we leave a message? ☐ Yes ☐ No<br>If yes, what message should we leave? __________<br>How should we identify ourselves? __________
Work Phone Number: (____)________________<br>☐ Refused / NA / Unknown<br>Best days/times to call: ***(Circle all days that apply)***<br>Weekends: Sa Su<br>Btw __|__ : __|__ AM/PM & __|__ : __|__ AM/PM<br>Weekdays: M Tu W Th F<br>Btw __|__ : __|__ AM/PM & __|__ : __|__ AM/PM	Can we call this person at work? ☐ Yes ☐ No<br>If yes, can we leave a message? ☐ Yes ☐ No<br>If yes, what message should we leave? __________<br>How should we identify ourselves? __________
Cell Phone Number: (____)________________<br>☐ Refused / NA / Unknown	Can we call this person's cell phone and leave a voice message? ☐ Yes ☐ No<br>If yes, what message should we leave? __________

	If no, can we send a text message?	☐ Yes ☐ No
	If yes, what message should we send?	________________<br>________________<br>________________<br>________________
E-mail address: ___________________________<br>☐ Refused / NA / Unknown	Can we email this person?	☐ Yes ☐ No
	If yes, what message should we send?	________________<br>________________<br>________________<br>________________

ATN 129
VIROLOGY RESULTS FORM

SID: 1 2 9 |__|__|__|__|__|__| **SITE CODE:** |__|__|

PID: |__|__|__|__|__|__|__| **SPECIMEN COLL. DATE:** |__|__|-|__|__|-|__|__|__|__|
m m d d y y y y

Visit: ☐ Screening ☐ Baseline ☐ Week 16 ☐ Week 28 ☐ Week 52

INSTRUCTIONS: Complete this form at each study visit requiring HIV-1RNA plasma viral load testing results. In the header, record the date the specimen was collected. If a required specimen, whether initial or repeat, was not collected, record the date the specimen was expected to be collected. Complete a separate form for each collection date if testing needs to be repeated for any reason.

If specimen collection took place prior to the study visit (i.e., historical data), enter this form into Remote Data Capture (RDC) within 14 calendar days after the study visit date. For all other specimen collections, enter this form into RDC within 14 calendar days after the specimen collection date.

[1] **Reason sample not collected or results not available:**

2 = Collected; hemolyzed or clotted
3 = Collected; container lost, damaged or undeliverable
4 = Collected; quantity not sufficient
5 = Collected; results pending
21 = Not collected; poor venous access
22 = Not collected; inadequate time
23 = Not collected; subject/legal guardian refused
27 = Not collected; subject did not show
99 = Other

[2] **Viral load result:** Record the numerical value of the result in the spaces provided and check the symbol that describes what the numerical value represents, whether equal to, less than or greater than the recorded value.

For a result that is reported as "below the lower limit of detection," record the assay's lower limit of detection as the numerical value and check the "less than (<)" symbol. For a result that is reported as "above the upper limit of quantitation," record the assay's upper limit of quantitation as the numerical value and check the "greater than (>)" symbol.

[3] **Assay Type Code:** Refer to "HIV-1 RNA Assay Type Codes" under Miscellaneous Forms on the ATN General Forms page in the Protocols and Forms category on the ATN website.

1. Study visit type: ☐ Screening
   (Check only one) ☐ Baseline ☐ Week 16 ☐ Week 28
   ☐ Week 52

2. Data obtained from: ☐ Chart Review
   ☐ Viral Load Collected Specifically for Study

3. Is there a result to report? ☐1 Yes ***(Go to Q3)***
   ☐0 No

   3a. If a sample was not collected or result is not available, indicate reason[1]: |__|__|[1] If '99', specify ______________________
   (End of form)

SID: 1 2 9 ___ ___ ___ ___ ___

PID: ___ ___ ___ ___ ___ ___ **SPECIMEN COLL. DATE:** ___ ___ / ___ ___ / ___ ___ ___ ___ (m m d d y y y y)

4. Viral load result[2] ___ ___ , ___ ___ ___ , ___ ___ ___ copies/ml
   - ☐1 Equal to (=)
   - ☐2 Less than (<)
   - ☐3 Greater than (>)

   4a. Based on this result, does the protocol require a repeat HIV-1 RNA assay?
   - ☐1 Yes
   - ☐0 No

5. Assay Type Code[3]: ___ ___ ___

   5a. If Assay Type '99', specify: ____________________________

   5b. Assay's lower limit of detection: ___ ___ ___ copies/ml
   - ☐ Unknown

   5c. Assay's upper limit of quantitation: ___ ___ , ___ ___ ___ , ___ ___ ___ copies/ml
   - ☐ Unknown

ATN 129
NAIL SPECIMEN TRACKING FORM

SID: 1 2 9 |__|__|__|__|__|__|__|__| **SITE CODE:** |__|__|

PID: |__|__|__|__|__|__|__| **SPECIMEN COLL. DATE:** |__|__|-|__|__|-|__|__|__|__|
m m d d y y y y

VISIT: Specimen Tracking

INSTRUCTIONS: Complete this form to document the collection of nail specimen for testing. ATN 129 site research staff must complete their designated sections, enter into Qualtrics and upload a copy of this form into Dropbox. You must enter the form into Qualtrics and upload into Dropbox within 24 hours from the specimen collection date.

Complete a separate Specimen Tracking Form for each collection date if specimens are collected on different days or if a specimen collection needs to be repeated for any reason.

ENTER THE APPROPRIATE CODE FOR EACH SPECIMEN IN Q6 – Q7

Reasons Specimens Not Collected[1] ***(Record all that apply)***:
21 = Not collected; inadequate time
22 = Not collected; subject/legal guardian refused
23 = Not collected; subject unable to provide specimen
24 = Not collected; previously collected
99 = Other ***(Specify)***

Specimen Collection Codes[2]**:**
1 = Collected as required
2 = Collected; quality questionable
3 = Collected; quantity not sufficient
99 = Other ***(Specify on Data Explanation Form)***

Specimen Condition Codes[3]**:**
1 = Good
2 = Good, but received outside required timeframe
3 = Inadequate weight
99 = Other ***(Specify on Data Explanation Form)***

Q1 - 5: To be completed by Site Staff

1. Study visit type:

   (Check only one)

   ☐ Baseline
   ☐ Week 16
   ☐ Week 28
   ☐ Week 52

2. Visit Identification (VID): ____________________

3. Indicate specimen collection type:

   ☐1 Initial
   ☐2 Repeat

Assigning Visit Identification Codes:

**Visit Week**	**VID**
**Baseline**	**0.0 BL**
**Week 16**	**16.0 WK**
**Week 28**	**28.0 WK**
**Week 52**	**52.0 WK**

SID: 1 2 9 |___|___|___|___|___|___|

PID: |___|___|___|___|___|___|

SPECIMEN COLL. DATE: |___|___|-|___|___|-|___|___|___|___| (m m d d y y y y)

4. Were required nail specimen collected?
   (Check only one)
   - ☐ 1 Yes, all
   - ☐ 2 Yes, some
   - ☐ 0 No, specify:______________________

   4a. Reason(s) specimens not collected: |___|___|[1] |___|___|[1] |___|___|[1] |___|___|[1] |___|___|[1]
   If '99', specify ______________________________

5. Type(s) of specimens collected:
   (Check all that apply, then complete only those questions indicated in the parenthesis on your selections.)
   - ☐ 1 Fingernail
   - ☐ 2 Toenail

6. **Fingernail**

To be completed by Site Staff

a. Collection time ***(Use 24-hr clock)*** |___|___|:|___|___| (h h m m)

b. Approximate weight collected |___|___|.|___| mg

c. Specimen Collection Code |___|___|[2]

d. Specimen Condition Code |___|___|[3]

Protocol Requirements:

100 mg of fingernail
or
100 mg of toenail

7. **Toenail**

To be completed by Site Staff

a. Collection time ***(Use 24-hr clock)*** |___|___|:|___|___| (h h m m)

b. Approximate weight collected |___|___|.|___| mg

c. Specimen Collection Code |___|___|[2]

d. Specimen Condition Code |___|___|[3]

Protocol Requirements:

100 mg of fingernail
or
100 mg of toenail

ATN 129
OFF STUDY FORM

SID: |1|2|9|___|___|___|___|___|___| **SITE CODE:** |___|___|

PID: |___|___|___|___|___|___|___| **OFF STUDY DATE:** |___|___|-|___|___|-|___|___|___|___|
m m d d y y y y

VISIT: ☐ Week 4 ☐ Week 16 ☐ Week 28 ☐ Week 52 ☐ Prem D/C

INSTRUCTIONS: Complete this form when the participant discontinues participation in the study for any reason.

- If the participant attends a designated premature discontinuation visit, check Prem D/C for the visit type and record the date of the visit as the off study date.
- If the participant does not attend a required premature discontinuation visit, check the last study visit accounted for, whether kept or missed, for the visit type. Record the date that the decision was made to discontinue collecting any further study data on the participant as the off study date (i.e., date site decides to stop attempts to get participant in to conduct the Prem DC visit).
- If the participant comes off study due to death, the off study date is the date of site awareness of the death.

1. Indicate the reason for study discontinuation: ***(Check only one)***

☐1 Completed study

☐2 Participant withdraws consent/assent or parent/legal guardian withdraws permission.

☐3 Moved out of area

☐4 Lost to follow-up

☐8 Participant becomes incarcerated or detained.

☐9 Development of an untoward event that warrants discontinuation from study ***(Complete Monitoring Untoward Event Form, if warranted)***

☐10 Subject fails to comply with the study requirements so as to cause harm to himself/herself or seriously interfere with the validity of the study results.

☐11 Investigator determines that further participation would be detrimental to the health or well-being of the subject.

☐12 Participant develops a health problem and needs treatment that would affect the results of this study.

☐13 Study was stopped for other administrative reasons

☐78 Study is stopped by a government agency such as the National Institutes of Health (NIH).

☐75 The investigator determined that receipt of additional study intervention is no longer in the participant's best interest.

☐79 Death ***(Complete Monitoring Untoward Event Form)***

Date of Death: |___|___|-|___|___|-|___|___|___|___|
m m d d y y y y

☐99 Other, specify: ____

SID: 1 2 9 __ __ __ __ __ __

PID: __ __ __ __ __ __ **OFF STUDY DATE:** __ __ - __ __ - __ __ __ __
m m d d y y y y

Investigator/Designee: ______________________ ______________________ __ __/__ __/__ __ __ __
Signature Print Name m m d d y y y y

ATN 129
PREMATURE DISCONTINUATION FROM STUDY INTERVENTION FORM

SID: 1 2 9 |__|__|__|__|__|__|__|__| **SITE CODE:** |__|__|

PID: |__|__|__|__|__|__| **DATE:** |__|__|-|__|__|-|__|__|__|__| (m m d d y y y y)

VISIT: Prem Study Intervention D/C

INSTRUCTIONS: Complete this form if the participant is prematurely discontinued from the study intervention(s) for any reason (refer to ATN 129 protocol, section 10.5.1 for criteria). **In the header, record the date the decision was made to permanently discontinue the study intervention(s).**

1. Date of last known study intervention |__|__|-|__|__|-|__|__|__|__| (m m d d y y y y)

2. Indicate the primary reason for premature discontinuation from study intervention: ***(Check only one)***
   - ☐ 1 Subject fails to comply with the study requirements so as to cause harm to himself/herself or seriously interfere with the validity of the study results.
   - ☐ 2 Investigator determines that further participation would be detrimental to the health or well-being of the subject.
   - ☐ 3 Participant develops a health problem and needs treatment that would affect the results of this study.
   - ☐ 4 Participant withdraws consent/assent or parent/legal guardian withdraws permission.
   - ☐ 5 Participant becomes incarcerated or detained.
   - ☐ 6 Study is stopped by a government agency such as the National Institutes of Health (NIH).
   - ☐ 7 Study is stopped for other administrative reasons.
   - ☐ 8 Other, specify:________________________
   - ☐ 9 Subject prematurely discontinued from study. ***(Select this option only if subject is also terminating study participation and other reasons listed on this form do not apply.)***
   - ☐ 75 The investigator determined that receipt of additional study intervention is no longer in the participant's best interest.
   - ☐ 99 Other, specify: ____

3. Are there any other contributing factors for premature discontinuation from study intervention?

ATN 129
PREMATURE DISCONTINUATION FROM STUDY INTERVENTION FORM

SID: 1 2 9 ___ ___ ___ ___ ___ ___ **SITE CODE:** ___ ___

PID: ___ ___ ___ ___ ___ ___ **DATE:** ___ ___ / ___ ___ / ___ ___ ___ ___ (m m d d y y y y)

VISIT: Prem Study Intervention D/C

☐1 Yes

☐0 No ***(End of form)***

Specify: ____

____

____

ATN 129
VISIT REPORT FORM

SID: 1 2 9 ___ ___ ___ ___ ___ ___ **SITE CODE:** ___ ___

PID: ___ ___ ___ ___ ___ ___ **VISIT DATE:** ___ ___-___ ___-___ ___ ___ ___ (m m d d y y y y)

VISIT: ☐ Pre-entry
☐ Baseline ☐ Week 16 ☐ Week 28 ☐ Week 52

INSTRUCTIONS: Complete this form for each study visit, whether it is kept or missed. In the header, record the study visit date. If the study visit was missed, record the last date of the study visit window.

1. Was the study visit conducted?
   - ☐1 Yes; on target date ***(Form Completed, FINISHED)***
   - ☐2 Yes; not on target date, but within the study visit window ***(Complete Q2, then FINISHED)***
   - ☐3 Yes, outside the study visit window ***(Answer ALL Questions-2, 3, & 4)***
   - ☐0 No ***(Go to Q4)***

2. Study Visit Window:

   Early Date: ___ ___-___ ___-___ ___ ___ ___ (m m d d y y y y)

   Late Date: ___ ___-___ ___-___ ___ ___ ___ (m m d d y y y y)

3. Was prior permission obtained from the Protocol Team or Chair to conduct this visit?
   - ☐1 Yes
   - ☐0 No ***(Notify protocol team via the ATN Query & Notification System)***

REMINDER: Complete Q4 only if the study visit was conducted outside the study visit window or was missed.

4. Reason visit was missed or conducted outside the study visit window: ***(Check only one)***
   - ☐1 Unable to locate or contact
   - ☐2 Consent or assent withdrawn
   - ☐3 Moved out of area
   - ☐4 Illness
   - ☐8 Incarceration or detention
   - ☐9 Transportation Issues
   - ☐10 Child care issues

SID: X X X ___ ___ ___ ___ ___

PID: ___ ___ ___ ___ ___ ___

VISIT DATE: ___ ___ / ___ ___ / ___ ___ ___ ___
m m d d y y y y

- ☐ 11 Conflicts with work or school schedule
- ☐ 12 Other competing appointments
- ☐ 13 Out of town
- ☐ 14 Unavailable during study visit window, reason unknown
- ☐ 15 Refused participation
- ☐ 16 Parent or legal guardian withdrew permission
- ☐ 17 Hospitalization
- ☐ 99 Other, specify:_______________________

OTHER COMMENTS:____

____

ATN 129
MONITORING UNTOWARD EVENT FORM

SITE CODE: |_____|_____|

SID : |_____|_____|_____|_____|_____|_____|_____|_____|

PID: |_____|_____|_____|_____|_____|_____|

DATE OF SITE AWARENESS: |____|____|-|____|____|-|____|____|____|____|
m m d d y y y y

VISIT: Untoward Event

INSTRUCTIONS: Complete a separate form for each untoward event – whether it involves a participant, study staff, or community. In the header, record the participant's SID and PID if the event is being reported for the participant.

Notify the ATN 129 protocol team within 48 hours of site awareness of the untoward event using the ATN Query and Notification System accessible through the ATN website (www.atnonline.org).

Enter this form into the ATN 129 database via Qualtrics within 3 working days after the date of site awareness of the reported event. Refer to the ATN MOGO, Chapter 8 for further instructions.

[1]**Untoward event (Key word):** Record a term that best describes the nature of the untoward event being reported. If the untoward event resulted in hospitalization, do not report hospitalization. Instead, report the cause of the hospitalization, e.g., suicidal ideation.

1. Date of untoward event: |____|____|--|____|____|--|____|____|____|____|
m m d d y y y y

2. Untoward event reported for:
- ☐ 1 Participant
- ☐ 2 Staff
- ☐ 3 Community

3. Untoward event (Keyword)[1]: ______________________________________

4. Severity Grade: ☐1 Minimal impact, managed at the time of event with no consequences

☐2 Moderate impact, managed by referral for supplemental care or counseling

☐3 Major impact, needed immediate professional intervention with or without hospitalization

☐9 Community untoward event, grading not required

5. Relationship to study: ☐1 Related (Go to Q6)

☐2 Not related

5a. Provide alternate etiology: ____

____

6. Action taken: (Check all that apply) ☐1 None

☐2 Referral to Mental Health Professional

☐3 Hospitalization

☐4 Staff Training

☐99 Other, specify: ______________________

7. Untoward event summary (Include a description of the untoward event, relevant history, and management of the event):

____

____

____

____

8. Outcome:

____

____

____

____

9. Was participant discontinued from the study as a result of the event?

☐1 Yes (Complete Off Study Form)

☐0 No

☐3 Not Applicable (Reported staff or community event)

10. Date event reported to the protocol team via the ATN Query & Notification System

|_____|_____|--|_____|_____|--|_____|_____|_____|_____|
m m d d y y y y

APPENDIX V: IRB FORMS

RECRUITMENT SCRIPT
IRB COVER LETTER
SAMPLE ASSENT AND INFORMED CONSENT
SAMPLE AUTHORIZATION FOR THE USE AND DISCLOSURE OF PROTECTED HEALTH INFORMATION YOUTH

RECRUITMENT SCRIPT

REMINDER TO CLINICAL SITES: DO NOT USE THE PREAMBLE IN LOCAL CONSENTS:

NOTE FROM OHRP (OFFICE OF HUMAN RESEARCH PROTECTION) TO SITES ENROLLING PARTICIPANT IN THIS STUDY:

Please note that this sample language does not preempt or replace local IRB review and approval. Investigators are required to provide the local IRB with a copy of this sample language along with the language intended for local use. Local IRBs are required to weigh the unique risks, constraints, and population considerations as a condition of any approval. Any deletion or substantive change of information concerning risks or alternative treatment must be justified by the investigator, approved by the local IRB, and noted in the IRB minutes. Justification and IRB approval of such changes must be forwarded to the Westat Site Registration desk for any NICHD-sponsored trial/study, or as may be otherwise specified. Sponsor-approved changes in a protocol must be approved by the local IRB before use, unless intended for the elimination of apparent immediate hazard. New information shall be shared with existing participant at next encounter, with all new participant prior to involvement, or as the local IRB may otherwise additionally require.

NOTE FROM NICHD: Authorization for the Use and Disclosure of Protected Health Information (Privacy Rule- HIPAA 45 CFR Parts160, 164)

This is a sample authorization text as part of this consent form that individual sites may revise as they see fit. Individual sites are advised to have this document reviewed by their legal counsel because the liability pursuant to HIPAA falls on the individual institutions and providers. However, whatever format is adopted by an institution to obtain authorization must include these conditions in order to maintain the integrity of research confidentiality: (1) if incorporated into the consent form, the authorization should constitute the last page of the research consent form and be self-contained with its own signature line so that if the institution designates a non-research entity to track compliance, the authorization can be detached without disclosing any information about the nature of the research participation; and (2) the title of the research project in the authorization form should not disclose sensitive or health-related information about the individual providing the authorization.

TITLE OF STUDY:

COMPARING THE EFFECTIVENESS OF TWO ALCOHOL+ADHERENCE INTERVENTIONS FOR HIV+ YOUTH (Healthy Choices 2; HC2)

Protocol Co-Chair: Sylvie Naar-King, PhD **PHONE:** (248) 207-2903

Protocol Co-Chair: Phebe K. Lam, PhD **PHONE:** (519) 817-8871

IRB COVER LETTER

TO: [IRB Chair]

FROM:

RE: ATN 129 Protocol Version 1.0
Title: *Comparing the Effectiveness of Two Alcohol+Adherence Interventions for HIV+ Youth (Healthy Choices 2; HC2)*

DATE:

Our program is a clinical site of the Adolescent Medicine Trials Network for HIV/AIDS Interventions (ATN). The ATN is a cooperative agreement among 14 clinical sites, investigators, and the National Institutes of Health. The ATN was established in March of 2001 to develop and implement a wide array of interventions aimed at improving the health and reducing the HIV risk of American adolescents. Its mission includes community-based primary prevention as well as clinical management of HIV-infected adolescents.

The proposal before you is a NIAAA funded 5 year study that will use a Type 1 Effectiveness-Implementation Hybrid Design to pilot a sustainable model of Motivational interviewing (MI) implementation in real-world youth care settings towards the goals of: 1) examining the effectiveness, cost-effectiveness, and scalability of an efficacious behavioral intervention when delivered by Community Health Workers (CHWs) in real-world youth HIV care settings; 2) gathering information about who responds to the intervention and under what contexts; and 3) increasing understanding of the study intervention related barriers and facilitators for future implementation.

We appreciate your careful review of this proposed research and your consideration of our requests to make its implementation practicable. Please do not hesitate to contact xxxxx, if additional information is required or desirable.

SAMPLE ASSENT AND INFORMED CONSENT

ASSENT AND INFORMED CONSENT

COMPARING THE EFFECTIVENESS OF TWO ALCOHOL+ADHERENCE INTERVENTIONS FOR HIV+ YOUTH
(Healthy Choices 2; HC2)

Protocol Co-Chair: Sylvie Naar-King, PhD
Protocol Co-Chair: Phebe K. Lam, PhD

WHAT IS THIS STUDY ABOUT?

Our sample in here

You are being asked to join a research study with HIV-positive youth through *(insert site name)* because you have the HIV virus and may be able to do better by making healthier choices. The research study is funded by the National Institute on Alcohol Abuse and Alcoholism (NIAAA), and are conducting it at Adolescent Trials Network (ATN) sites. About 500 youth between the ages of 16 and 24 years are being asked to take part in this research study. This study is being done at research sites in Detroit, MI; Philadelphia, PA; Chicago IL; Los Angeles, CA; and Memphis TN.

For you to decide whether or not you want to be in the study, you should know:

- Why the study is being done,
- What you will have to do during the study, and
- Any possible risks and benefits.

This is called informed consent/assent. This consent/assent form tells you about the research study. We will read the form to you and discuss it with you. Once you understand the study, you will be asked to sign this form if you want to take part in the study. You will be given a copy of this form to keep.

WHY IS THIS STUDY BEING DONE?

This research study is being done to learn more about healthy choices in youth living HIV. The researchers who are doing the study want to see if a type of counseling called Motivational Enhancement Therapy (MET) can help you do better with decreasing your alcohol use, and other things like seeing the doctor, and taking medicine. Also, the researchers would like to find out if doing better with these things reduces the amount of HIV virus in your blood and helps you feel better.

WHAT WILL I HAVE TO DO IN THE STUDY?

If you decide to join the study and sign the assent/consent form, you will complete several surveys. The interviewer will ask you about your physical and mental health, clinic visits, HIV medications, drug and alcohol use, and sex. Other questions will be asked about your relationships with family and friends. Then you will be assigned at random, like the flip of a coin, to have clinic-based visits or home-based (or a mutually agreed upon community setting visits) MET Healthy Choices sessions with a Community Health Worker. You will meet with the Community Health Worker for four 60-minute sessions over a period of 12 weeks. There will be 2 sessions the first month, 1 session the second month, and 1 session the third month. This is a total of 4 sessions

over 3 months (12 weeks). After your MET Healthy Choices sessions are complete, you will see the interviewer again to complete the same surveys at 16 weeks, 28 weeks, and 52 weeks.

We will also look at your medical records for information that is needed for the study. We will draw about 4 teaspoons of blood (similar to your usual blood draws at your regular clinic visit) from you to measure your HIV viral load if you have not had this done at *(insert site name)* within the last 4 weeks. We will also collect some nail samples (finger or toe nails) to measure any alcohol use. We will also ask you how we can find you for the rest of time during the research study. We will ask you about where you hang out and for names of friends and family who know how to find you. The visit will take about 1.5 to 2 hours. This is called a research visit. You will have a total of four of these research visits over 52 weeks.

WHAT ARE SOME POSSIBLE RISKS?
There are very few risks to you for being in the study. You may feel uncomfortable thinking about and answering questions about having HIV. If you do, there are people at *(insert site name)* for you to talk to. There can also be discomfort from drawing blood such as pain, swelling, bruising at the site, or feeling faint. These risks will be small since the person is trained in drawing blood. Another possible risk is having others find out how you answered questions. This is explained in the confidentiality section.

In order to protect your privacy, we will only try to reach you through phone calls or mailings to remind you about study visits if you have given your permission. If you give this permission, the only information that we will give out about why we are trying to contact you is what you agreed that it is okay for us to give out. This permission may be taken away by you at any time. If you have not told your parents/guardians or friends that you are in the study, there is a risk that they will find out during these contacts. Also, your parents/guardians/friends may find things (such as this consent form, business card, or compensation) that lead them to believe you are part of an HIV study.

HOW CAN THIS STUDY HELP ME?
Taking part in this study may help you make healthier choices. This means you might have fewer problems from HIV, such as fewer illnesses and hospitalizations. However, there are no guarantees that any good things will happen directly to you. The information that is learned will be used to see if MET Healthy Choices can help youth living HIV make behavior changes.

ARE THERE ANY ALTERNATIVES TO TAKING PART IN THIS STUDY?
If you choose not to take part in this study, your doctors and other health care staff can help you get in touch with counseling and clinical services that may help you. There are also other counseling services available in the community.

WHAT WILL I GET FOR TAKING PART IN THE STUDY?
As a thank you for your time and for taking part in the study, you will be compensated $50.00 per research visit with no incentives for the intervention sessions (MET Healthy Choices Sessions).

WHAT HAPPENS IF I AM INJURED DURING THE STUDY?
It is not likely that you will be injured as a result of being in this study. But if you are, you will be given treatment right away. The cost for this treatment will be charged to you or your insurance company. There is no payment available through either *(insert site name)* or the study sponsors.

WHAT ARE THE COSTS TO ME?
There are no costs to you for being in the study. We will cover the cost of your HIV viral load test and transportation costs to come to the clinic as part of the research study.

WHAT IF I WANT TO STOP TAKING PART IN THE STUDY?
Your participation in this study is voluntary. You may decide to stop taking part in the study at any time. If you choose to not be in the study or withdraw from the study, you can still receive clinic care and all services from *(insert site name).*

WILL MY RECORDS BE KEPT PRIVATE?
Information that is gathered from this study will be kept confidential as permitted by law. A Certificate of Confidentiality has been obtained from the Federal Government for this study to help protect your privacy. This Certificate means that the researchers cannot be forced to tell people who are not connected with the study, including courts, about your participation, without your written consent. If we see [learn] something that would immediately put you or others in danger, we may discuss it with you, if possible, or seek help.

Numbers are used on the surveys and all study records rather than names. Only the research staff will know what names go with each number. Information about you is not shared with *(insert site name)* your doctors or put in your medical record. Information from the study may be published or given to other people doing research, but names are never used. The information we get from you about how to find you will be kept separate from the research record. The only information that we will give out when we are trying to contact you will be information that you have given permission for us to give out.

WHAT IF I HAVE QUESTIONS?
If you have questions about taking part in the study, now or at any time, (*insert name of site PI)* can be reached at *(insert telephone number of site PI).* If you have questions about your rights as a research participant, you can call the chairperson of *(insert name and telephone number of local IRB)*.

STATEMENT OF CONSENT
(NOTE: This is only a suggested signature format. Sites may use their own signature page.)

The purpose of the research study, what you will have to do during the study, and the risks and benefits of the study has been explained to you. You have been given time to ask any questions you wanted to about the study and you are satisfied with the answers you were given. You have been told that you are not giving up any of your legal rights by taking part in this study. You have been told that you may refuse to take part in the study and that you can stop participating at any time. You will be given a copy of this consent form. By signing this consent form, you are agreeing to take part in this study and giving your permission for the study investigators to collect and use the information and specimens needed for the purposes of this study. If you voluntarily agree to take part in this study, please sign your name below.

____
Participant Name (print) Participant Signature Date

____
Witness Name (print) Witness Signature Date

PI or Designee's statement:

I have reviewed this study and the consent/assent form with the participant. To the best of my knowledge, he or she understands the purpose, procedures, risks and benefits of the study.

____

PI or Designee Name (print)   PI or Designee Signature   Date

---

NOTE: This consent form with the original signatures MUST be retained on file by the principal investigator. A copy must be given to the volunteer. A copy should be placed in the volunteer's medical record, if applicable.

SAMPLE

AUTHORIZATION FOR THE USE AND DISCLOSURE OF PROTECTED HEALTH INFORMATION YOUTH

TITLE OF STUDY: COMPARING THE EFFECTIVENESS OF TWO ALCOHOL+ADHERENCE INTERVENTIONS FOR HIV+ YOUTH (Healthy Choices 2; HC2)

Principal Investigator: Sylvie Naar-King, PhD

This section is asking you to authorize the use and disclosure of your health information for the study named COMPARING THE EFFECTIVENESS OF TWO ALCOHOL+ADHERENCE INTERVENTIONS FOR HIV+ YOUTH (Healthy Choices 2; HC2). To do that you need to know:

►The kind of health information about you that the study will collect and use; this information includes (insert all that apply):

- ♦ interviews about your health, relationships, and behaviors
- ♦ biological specimens (ex., HIV-Viral Load)
- ♦ contact information

►The reasons that we are doing this study, which have been described to you earlier in the Informed Consent section named "Purpose of the Study");

►The persons who will collect and use your information for this study:

1. Dr. (insert site PI) (or whomever may replace this doctor) who is responsible for collecting the information here at (insert institution name).

►The persons who will receive and use your information for this study:

- ♦ The Adolescent Medicine Trials Network will receive information without your name from this and other sites to do this study.

►This authorization will end at the end of this study (approximately XX months).

►You can stop the use of your information in this research study by sending a written request to Dr. (insert name and address of PI) (or whoever may replace this doctor). If you decide to revoke your authorization:

- ♦ no more information will be collected from you or your records for the research study from the time the written request is received
- ♦ The study will only use the information it has already collected from you before you sent the written request if it will harm the study to stop using your information.

►When you sign this document and authorize the use and disclosure of your health information for this research, the information disclosed may no longer be protected by the federal privacy regulations found at 45 CFR Part 164. But, the researchers for this study can only use or disclose your health information for purposes that are approved by an Institutional Review Board or as required by law or regulation.

STATEMENT OF CONSENT

(NOTE: This is only a suggested signature format. Sites may use their own signature page.)

The details of this authorization have been explained to you and you have been given the chance to ask any questions you

wish.

If you agree to allow the researchers to use and disclose your health information for the purpose of this study, please sign your name below.

Participant Name (print)	Participant Signature	Date
Witness Name (print)	Witness Signature	Date

PI or Designee's Statement:

I have reviewed the authorization for the use and disclosure of protected health information with the participant. To the best of my knowledge, he or she understands the meaning of this authorization.

PI or Designee Name (print)	PI or Designee Signature	Date

NOTE: This authorization form with the original signatures MUST be retained on file by the principal investigator. A copy must be given to the volunteer. A copy should be placed in the volunteer's medical record, if applicable.
