# Supplementary material for: Comparative Effectiveness of Community-Based vs Clinic-Based Healthy Choices Motivational Intervention to Improve Health Behaviors Among Youth Living With HIV: A Randomized Clinical Trial
Source: JAMA Netw Open. 2020 Aug 26;3(8):e2014650. doi: 10.1001/jamanetworkopen.2020.14650 (PMC7450347; doi:10.1001/jamanetworkopen.2020.14650)
Supplement: Supplement 2. — eAppendix. Data Analysis eTable 1. Analyses of Attrition eTable 2. Comprehensive Model Coefficients [file jamanetwopen-3-e2014650-s002.pdf]

Supplementary Online Content

Naar S, Robles G, MacDonell KK, et al. Comparative effectiveness of community-based vs clinic-based healthy choices motivational intervention to improve health behaviors among youth living with HIV: a randomized clinical trial. *JAMA Netw Open*. 2020;3(8):e2014650. doi:10.1001/jamanetworkopen.2020.14650

eAppendix. Data Analysis

eTable 1. Analyses of Attrition

eTable 2. Comprehensive Model Coefficients

This supplementary material has been provided by the authors to give readers additional information about their work.

© 2020 Naar S et al. *JAMA Network Open.*

eAppendix. Data Analysis

Post Hoc Power Analysis

Because of slow recruitment, post hoc power to detect a between-condition difference in the outcome at was calculated using the Mixed Models Tests for Two Means at the end of a Follow-up in 2-Level Hierarchical Design module in PASS. At 52 weeks, the observed between group difference in log Viral Load was 0.60 (SD=1.40). Assuming a within-participant correlation (ρ) of 0.40, a sample with 183 youth would have power = 0.85 to reject the null hypothesis. In the current study, observed data on log VL were available from 102 youth, power under those circumstances is 0.66. At the final follow-up, the observed between group difference in alcohol use severity scores was 0.5 (SD=8.42). Assuming a within-participant correlation (ρ) of 0.45, a sample with 183 youth would have power = 0.60 to reject the null hypothesis. In the current study, observed data on alcohol severity were available from 122 youth, power under those circumstances is 0.5. Power for the alcohol frequency was calculated using the test for the ratio of two negative binomial rates module in PASS. The observed between group difference was 3.6 drinks and the dispersion parameter for the negative binomial distribution was approximately 1.7. A sample with 183 youth would have power = 0.60 to reject the null hypothesis. In the current study, observed data on alcohol severity were available from 120 youth, power under those circumstances is 0.45.

Model Fit Criteria

All models were estimated using full-information maximum likelihood (FIML) estimation. Good model fit was assumed when the $\chi 2$/df ratio was 3 or less, root-mean square error of approximation (RMSEA) ≤ 0.05, Tucker-Lewis fit index (TLI) > 0.95, and comparative fit index (CFI) > 0.95 [51-53]. We tested the effects of outliers in the alcohol frequency variable in a sensitivity analysis using scores Winsorized at 97.5%. This approach did not substantially change the estimates, but because it improved the model fit we use this results in the report.

Sequential Model Building Details

We utilized a stepwise model testing procedure in order to confirm hypotheses about the need for multiple slopes in piecewise latent growth curve analyses for viral load and alcohol frequency models. First, an initial model was estimated which included only an intercept and a slope that captured pre-to-post intervention change. Subsequently, we estimated a model which included the addition of a second slope element estimating the linear change over the post-intervention follows up periods. We then, identified the best fitting growth model for each outcome (one slope vs. two slopes). The best fitting model then was used to estimate between-condition differences by adding condition (i.e., home- vs. clinic-based intervention) as a predictor of the latent growth factors. In the final model, we added a fixed effect of site. For all outcomes, this stepwise procedure confirmed a priori assumptions that two slopes would be required to model trajectories.

**eTable 1. Analyses of Attrition**											
		**16wk**				**28wk**				**52wk**	
	Incomplete	Complete			Incomplete	Complete			Incomplete	Complete	
	n (%)	*n (%)*			*n (%)*	*n (%)*			*n (%)*	*n (%)*	
Total	47 (25.7)	136 (74.3)			65 (35.5)	118 (64.5)			61 (33.3)	122 (66.6)	
Condition			$\chi^2(1)=1.729$				$\chi^2(1)=.369$				$\chi^2(1)=.000$
Home	27 (57.4)	63 (46.3)			30 (46.2)	60 (50.8)			30 (49.2)	60 (49.2)	
Clinic	20 (42.6)	73 (53.7)			35 (53.8)	58 (49.2)			31 (50.8)	62 (50.8)	
Site			$\chi^2(4)=1.646$				$\chi^2(4)=3.656$				$\chi^2(4)=1.184$
LA	7(11.3)	17(14)			7(10.4)	17(14.7)			7(11.5)	17(14)	
Philly	15(24.2)	26(21.5)			13(19.4)	28(24.1)			15(24.6)	26(21.3)	
Chicago	18(29)	27(22.3)			16(23.9)	29(25)			17(27.9)	28(22.9)	
Memphis	4(6.5)	8(6.6)			7(10.4)	5(4.3)			4(6.5)	8(6.6)	
Detroit	18(29)	43(35.5)			24(35.8)	37(31.9)			18(29.5)	43(35.2)	
Race 4 Cat			$\chi^2(3)=7.762$				$\chi^2(3)=4.471$				$\chi^2(3)=2.640$
Black	39 (81.3)	112 (83)			54(80.6)	97(83.6)			49(80.3)	102(83.6)	
Latino	2 (4.2)	14 (10.4)			4(6)	12(10.3)			6 (9.8)	10(8.2)	
White	0	3 (2.2)			1(1.5)	2(1.7)			0	3(2.5)	
Other	7 (14.6(=)	6 (4.4)			8(11.9)	5 (4.3)			6(9.8)	7(5.7)	
Race			$\chi^2(5)=5.162$				$\chi^2(5) = 4.001$				$\chi^2(5)=.743$
Asian	0 (0)	1(.5)			1(1.5)	0(0)			1(1.6)	0(0)	
Black	38(80.9)	113(83.1)			52(80)	99(83.9)			49(80.3)	102(83.6)	
Native American	0(0)	1(.7)			0(0)	1(.0)			0(0)	1(.8)	
White	1(2.1)	7(5.1)			2(3.1)	6(5.1)			3(4.9)	5(4.1)	
Mixed Race	8(17)	11(8.1)			9(13.8)	10(8.5)			7(11.5)	12(9.8)	
Other	0(0)	3(2.2)			1(1.5)	2(1.7)			1(1.6)	2(1.6)	
Ethnicity			χ2(1)=.547				χ2(1)=.500				χ2(1)=2.179
Hispanic	4(8.5)	17(12.5)			6(9.2)	15(12.7)			10(16.4)	11(9)	
Non-Hispanic	43(91.5)	119(87.5)			59(90.8)	103(87.3)			51(83.6)	111(91)	
Education			$\chi^2(2)=.061$				$\chi^2(2)=.275$				$\chi^2(2)=1.491$

© 2020 Naar S et al. *JAMA Network Open.*

< High School	14(29.8)	38(27.9)			20(30.8)	32(27.1)			14(23)	38(31.1)	
High School or GED	17(36.2)	50(36.8)			23(35.4)	44(37.3)			23(37.7)	44(36.1)	
Some college	16(34)	48(35.3)			22(33.8)	42(35.6)			24(39.3)	40(32.8)	
Employed			$\chi^2(1)$=.142				$\chi^2(1)$=.089				$\chi^2(1)$=2.460
Yes	22(46.8)	68(50)			31(47.7)	59(50)			25(41)	65(53.3)	
No	25(53.2)	68(50)			34(52.3)	59(50)			36(59)	57(46.7)	
Sexual Identity			$\chi^2(5)$=6.864				$\chi^2(5)$=3.862				$\chi^2(5)$=4.762
Heterosexual	13(27.7)	25(18.4)			16(24.6)	22(18.6)			13(21.3)	25(20.5)	
Gay	26(55.3)	76(55.9)			35(53.8)	67(56.8)			37(60.7)	65(53.3)	
Lesbian	1(2.1)	0(0)			1(1.5)	0(0)			1(1.6)	0(0)	
Bisexual	6(12.8)	31(22.8)			12(18.5)	25(21.2)			9(14.8)	28(23)	
Questioning	0(0)	2(1.5)			0(0)	2(1.7)			0(0)	2(1.6)	
Other	1(2.1)	2(1.5)			1(1.5)	2(1.7)			1(1.6)	2(1.6)	
Gender Identity			$\chi^2(4)$=3.264				$\chi^2(4)$=4.906				$\chi^2(4)$=10.053*
Male	35(74.5)	110 (80.9)			48(73.8)	97(82.2)			47(77)	98(80.3)	
Female	7(14.9)	18 (13.2)			10(15.4)	15(12.7)			6(9.8)	19(15.6)	
Trans Male	2(4.3)	2(1.5)			1(1.5)	3(2.5)			1(1.6)	3(2.5)	
Trans Female	3(6.4)	4(2.9)			5(7.7)	2(1.7)			6(9.8)	1(.8)	
GNC	0(0)	2(1.5)			1(1.5)	1(.8)			1(1.6)	1(.8)	
	M (SD)	*M (SD)*			*M (SD)*	*M (SD)*			*M (SD)*	*M (SD)*	
Age	21.57 (1.64)	21.31 (1.88)	t (179) = 0.82		21.62 (1.76)	21.24 (1.85)	t (179) = 1.33		21.61 (1.71)	21.26 (1.88)	t (179) = 1.22
BL HIV VL	3.87 (1.18)	3.62 (1.25)	t (181) = 1.22		3.61 (1.15)	3.72 (1.28)	t (181) = -0.59		3.89 (1.17)	3.58 (1.26)	t (181) = 1.59
Alcohol Frequency	23.13 (42.09)	16.20 (33.73)	Wald χ^2 = 1.90		17.94 (36.81)	18.00 (35.91)	Wald χ^2 = 0.00		17.85 (37.00)	18.04 (35.75)	Wald χ^2 = 0.002
Alcohol ASSIST	11.43 (10.09)	11.83 (8.30)	t(181) = -0.27		10.72 (8.59)	12.28 (8.96)	t (181) = -1.14		10.67 (9.35)	12.25 (8.56)	t (181) = -1.14

© 2020 Naar S et al. *JAMA Network Open.*

eTable 2. Comprehensive Model Coefficients

	Viral Load				*ASSIST (Severity)††*				*Number of Drinks (Frequency)*		
	B	*95% CI*	*P value*		*B*	*95% CI*	*P value*		*B*	*95% CI*	*P value*
**Intercept**											
Threshold/Intercept	3.52	(3.12, 3.91)	<.001		1.74	(-0.66, 4.14)	.16		1.62	(1.21, 2.02)	<.001
Clinic	0.05	(-0.25, 0.37)	.725		1.76	(-0.59, 1.11)	.142		0.28	(-0.08, 0.64)	.121
Site†											
Los Angeles	-0.35	(-0.96, 0.26)	.266		-0,27	(-3.75, 3.21)	.878		0.00	(-0.64, 0.63)	.989
Chicago	1.17	(0.73, 1.60)	<.001		1.08	(-2.02, 4.18)	.496		0.29	(-0.24, 0.83)	.280
Memphis	-0.43	(-1.14, 0.28)	.241		0.07	(-2.83, 2.97)	.962		-0.43	(-0.34, 1.21)	.272
Detroit	-0.24	(-0.69, 0.20)	.292		3.56	(0.62, 6.50)	.017		-0.01	(-0.48, 0.45)	.948
**Slope 1**											
Intercept	-0.27	(-0.43, -0.10)	.001						-0.09	(-0.21, 2.03)	.086
Clinic	0.00	(-0.13, 0.12)	.984						0.05	(-0.06, 0.15)	.394
Site†											
Los Angeles	-0.01	(-0.86, 0.77)	.916						-0.07	(-0.23, 0.08)	.343
Chicago	-0.28	(-1.85, -0.44)	.007						-0.10	(-0.26, 0.06)	.232
Memphis	0.14	(-0.48, 1.70)	.261						-0.15	(-0.41, 0.10)	.234
Detroit	-0.01	(-0.68, 0.61)	.920						-0.02	(-0.12, 0.15)	.781
**Slope 2**											
Intercept	0.00	(-0.06, 0.06)	.983		0.35	(-0.09, 0.79)	.125		-0.03	(-0.14 0.08)	.601
Clinic	-0.07	(-0.14, -0.01)	.022		-0.44	(-0.81, -0.06)	.021		-0.02	(-0.09, 0.05)	.601
Sites†											
Los Angeles	0.04	(-0.03, 0.11)	.241		-0.24	(-0.83, 0.36)	.436		0.02	(-0.11, 0.15)	.751
Chicago	0.02	(-0.03, 0.09)	.398		-0.40	(-0.89, 0.09)	.113		0.02	(-0.10, 0.14)	.725
Memphis	0.02	(-0.05, 0.09)	.555		0.11	(-0.68, 0.91)	.779		0.09	(-0.06, 0.28)	.378
Detroit	0.04	(-0.03, 0.11)	.241		-0.41	(-0.89, 0.07)	.094		0.05	(-0.11, 0.16)	.394

†All models presented were analyzed with Philadelphia as the referent category.
†† Model adjusted for baseline ASSIST scores in the prediction of post-intervention follow-up values. The growth intercept is calculated at the 16-week follow-up and baseline scores are utilized as a fixed effect predictor of growth factors rather than an indicator.

© 2020 Naar S et al. *JAMA Network Open.*
