# Supplementary material for: Comparative Effectiveness of Community-Based vs Clinic-Based Healthy Choices Motivational Intervention to Improve Health Behaviors Among Youth Living With HIV: A Randomized Clinical Trial
Source: JAMA Netw Open. 2020 Aug 26;3(8):e2014650. doi: 10.1001/jamanetworkopen.2020.14650 (PMC7450347; doi:10.1001/jamanetworkopen.2020.14650)
Supplement: Supplement 3. — Data Sharing Statement [file jamanetwopen-3-e2014650-s003.pdf]

Data Sharing Statement

Naar. Comparative Effectiveness of Community-Based vs Clinic-Based Healthy Choices Motivational Intervention to Improve Health Behaviors Among Youth Living With HIV. *JAMA Netw Open*. Published August 26, 2020. 10.1001/jamanetworkopen.2020.14650

Data
Data available: Yes
Data types: Deidentified participant data
How to access data: These data can be requested through Sylvie.Naar@med.fsu.edu
When available: With publication

Supporting Documents
Document types: None

Additional Information
Who can access the data: Researchers whose proposed use of the data has been approved
Types of analyses: specified purpose
Mechanisms of data availability: after approval of a proposal
